# Bioactive Indolyl Diketopiperazines from the Marine Derived Endophytic *Aspergillus* *versicolor* DY180635

**DOI:** 10.3390/md18070338

**Published:** 2020-06-28

**Authors:** Yi Ding, Xiaojing Zhu, Liling Hao, Mengyao Zhao, Qiang Hua, Faliang An

**Affiliations:** State Key Laboratory of Bioreactor Engineering, East China University of Science and Technology, 130 Mei Long Road, Shanghai 200237, China; zjzsdy@163.com (Y.D.); 13761041863@163.com (X.Z.); holiday_hao1988@126.com (L.H.); myzhao@ecust.edu.cn (M.Z.); qhua@ecust.edu.cn (Q.H.)

**Keywords:** endophyte fungus, *Aspergillus versicolor*, diketopiperazines, ECD calculation, enantiomers

## Abstract

Four new indolyl diketopiperazines, aspamides A–E (**1**–**4**) and two new diketopiperazines, aspamides F–G (**5–6**), along with 11 known diketopiperazines and intermediates were isolated from the solid culture of *Aspergillus* *versicolor*, which is an endophyte with the sea crab (*Chiromantes haematocheir*). Further chiral high-performance liquid chromatography resolution gave enantiomers (+)- and (−)-**4**, respectively. The structures and absolute configurations of compounds **1**–**6** were determined by the comprehensive analyses of nuclear magnetic resonance (NMR), high-resolution mass spectrometry (HR-MS), and electronic circular dichroism (ECD) calculation. All isolated compounds were selected for the virtual screening on the coronavirus 3-chymoretpsin-like protease (Mpro) of Severe Acute Respiratory Syndrome Coronavirus 2 (SARS-CoV-2), and the docking scores of compounds **1**–**2**, **5**, **6**, **8** and **17** were top among all screened molecules, may be helpful in fighting with Corona Virus Disease-19 (COVID-19) after further studies.

## 1. Introduction

Endophytic fungi refer to harmless parasitic fungi that live in the internal organs of plants and animals without causing any adverse reactions. The host provides nutrients for endophytes, and endophytes produce bioactive substances, giving the host an advantage in survival competition [1]. Symbionts coexist with symptomless fish, sponges, algae, and soft corals that grow in a relatively harsh marine environment characterized by high salinity, scarce nutrients, and high osmotic and hydraulic pressures, which provides many environment-specific microorganisms that could coevolve with their hosts by undergoing the rapid and dynamic change of their genomes [2,3]. Thus, endophytic fungi are considered as a treasure trove of unique structural compounds and bioactive metabolites.

Indolyl diketopiperazines (IDKPs), cyclic dipeptides produced by the condensation of l-tryptophan and a second amino acid, were commonly isolated from fungi, especially from the genera *Aspergillus* and *Penicillium* [4,5]. IDKPs had drawn considerable attention from synthetic chemists, natural products researchers, and synthetic biologists for decades due to their significant biological activities, such as antiviral [6,7], anticancer [8,9,10], immunomodulatory [11,12], antioxidant [13], and α-glucosidase inhibitory activities [14]. Specifically, the vascular disrupting and tubulin-depolymerizing agent plinabulin, a synthetic analog based on the natural diketopiperazine (DKP) product halimide generated by the marine-derived *Aspergillus* sp. CNC-139, had entered the last stage of clinical study for the treatment of non-small-cell lung cancer [15,16]. Since the first IDKP alkaloid chaetomin isolated from the fungus *Chaetomium cochliodes* in the early 1940s, a series of DKPs and their biosynthesis clusters were reported [17,18,19,20,21,22]. In our continuous investigations of novel bioactive agents from the endophytic fungi [23,24], the endophytic strain *Aspergillus versicolor* DY180635 isolated from the sea crab was selected based on the bio-evaluation results. The ethyl acetate extracts of a rice solid culture of *A. versicolor* DY180635 showed 80% inhibition on the anti-inflammation model of the *Propionibacterium acnes*-induced THP-1 cells at the concentration of 0.1 mg/mL [25]. High-performance liquid chromatography (HPLC) analysis of the ethyl acetate extracts indicated the presence of IDKPs with a diode array detector (DAD) through ultraviolet characteristics at *λ*_max_ 236, 289, and 336 nm [26]. Thus, to discover structurally complex and/or bioactive DKPs, the spectroscopic-guided isolation was performed in this research.

**Figure 1:**
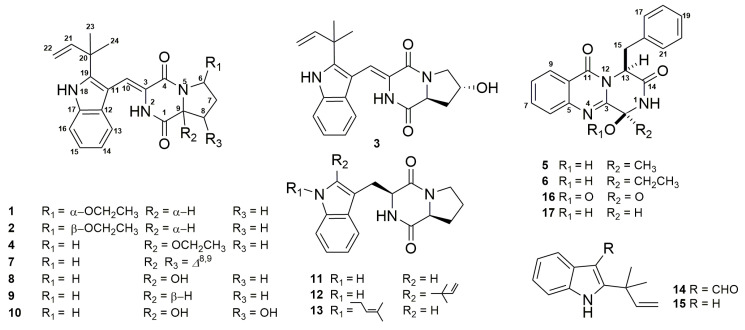
Structures of compounds **1–17**.

Spectroscopic-guided isolation resulted in the identification of four new IDPKs, aspamides A–E (**1–4**) and two new DPKs, aspamides F–G (**5–6**), along with 11 known diketopiperazines and intermediates from the ethyl acetate (EtOAc) extracts of the solid culture of *A. versicolor* (Figure 1). The couple of epimers **1–2** were the first samples of brevianamides with an oxygenated aza-acetal structure at the proline motif. All isolated compounds were tested for anti-inflammation in *P*. *acnes*-induced THP-1 cells. Unfortunately, none showed active effect. With the appearance and spread of SARS-CoV-2 at the end of 2019, compounds **1–17** were selected for the virtual screening on the 3CL hydrolase (Mpro) of SARS-CoV-2, which had been exploited as a potential drug target to fight COVID-19 [27]. The docking scores of compounds **1–2**, **5**, **6**, **8**, and **17** were top among all screened molecules (docking scores: −5.389, −4.772, −5.146, −4.962, −5.158), which may be helpful in fighting COVID-19 after further studies. Herein, we reported the isolation, structural identification, and bio-evaluation of isolated compounds.

## 2. Results and Discussion

The EtOAc extract of the rice solid culture of *A. versicolor* DY180635 was fractionated by column chromatography (CC) on macroporous adsorbent resin, silica gel, and octadecyl silane (ODS), as well as by preparative HPLC, to afford 15 DKPS and two intermediates. Six new DKPs, named as aspamides A**–**F (**1–6**), were determined by comprehensive spectroscopic analysis including ^1^H nuclear magnetic resonance (NMR), ^13^C NMR, HSQC, heteronuclear multiple bond correlation (HMBC), rotating frame Overhauser effect spectroscopy (ROESY), and high resolution electrospray mass spectrometry (HRESIMS) spectra. By comparing the NMR and ESIMS data to the reported literatures in detail, 11 known compounds were determined as brevianamides K, N, and M (**7**, **16**, **17**) [28], brevianamide Q (**8**) [29], brevianamides V, U (**9–10**) [20], brevianamide F (**11**) [30], deoxybrevianamide E (**12**) [31], *N*-Prenyl-*cyclo*-l-tryptophyl-l-proline (**13**) [32], 2-(2-methyl-3-en-2-yl)-1*H*-indole-3-carbaldehyde (**14**) [33], and 2-(1,1-Dimethyl-allyl)-1*H*-indol-3-ylmercuric acetate (**15**) [34]. Herein, the details of the isolation, structural elucidation of these new compounds, and their bioactivities are described.

Aspamide A (**1**) was isolated as a yellowish powder. The UV spectrum with *λ*_max_ (log*ε*) in methanol at 200 (6.13), 224 (6.13), 284 (5.46), and 341 (5.68) nm was indicative of indole functionality with an extended conjugation [26]. Its molecular formula was determined as C_23_H_27_N_3_O_3_ on the basis of high-resolution ESIMS (*m*/*z* 394.2117 [M + H]^+^, calcd. for C_23_H_28_N_3_O_3_, 394.2125) and ^13^C NMR data, requiring 12 degrees of unsaturation. The ^1^H NMR, ^13^C NMR, and heteronuclear multiple quantum correlation (HMQC) spectra (Table 1 and Figure S3 in Supporting Information) showed three methyl groups (*δ*_C_ 27.4, *δ*_H_ 1.45; *δ*_C_ 27.8, *δ*_H_ 1.49; *δ*_C_ 15.2, *δ*_H_ 1.13), three sp^3^ methylenes (including one oxygenated methylene) (*δ*_C_ 29.6, *δ*_H_ 1.75, *δ*_H_ 1.97; *δ*_C_ 25.9, *δ*_H_ 2.13, *δ*_H_ 2.27; *δ*_C_ 63.6, *δ*_H_ 3.65), two sp^3^ methine carbon signals (including one oxygen-bearing carbon) (*δ*_C_ 56.5, *δ*_H_ 4.57; *δ*_C_ 86.7, *δ*_H_ 5.59), six sp^2^ methines, one sp^2^ methylene, seven sp^2^, and one sp^3^ non-protonated carbon. The NMR data and UV absorptions were close to those of brevianamide V [20], with the exception that there was an additional oxygenated aza-acetal structure located at the proline motif (*δ*_C_ 86.7, *δ*_H_ 5.59; *δ*_C_ 63.6, *δ*_H_ 3.65; *δ*_C_ 15.2, and *δ*_H_ 1.13).

**Figure 2:**
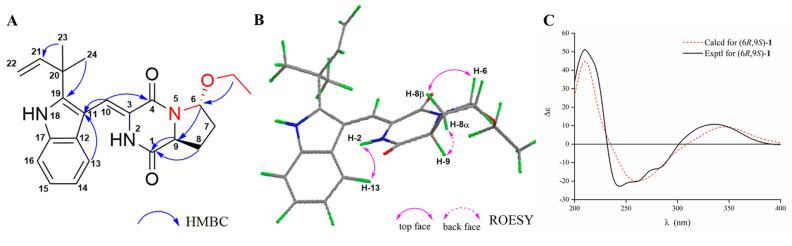
The key heteronuclear multiple bond correlation (HMBC) (**A**) and rotating frame Overhauser effect spectroscopy (ROESY) (**B**) correlations, and experimental and calculated electronic circular dichroism (ECD) spectra (**C**) of compound **1**.

Further information about the structure was derived from heteronuclear multiple bond correlation (HMBC) spectra analyses (Figure S4). The key HMBC correlations (Figure 2A) from OCH_2_CH_3_-6 (*δ*_H_ 3.65) to C-6 (*δ*_C_ 86.7), H-6 (*δ*_H_ 5.59) to C-9 (*δ*_C_ 56.5), H-9 (*δ*_H_ 4.57) to C-8 (*δ*_C_ 25.9) and C-1 (*δ*_C_ 165.9), and H-8α (*δ*_H_ 2.27) to C-9 were observed. These data suggested that an oxethyl group was located at C-6 and confirmed the oxygenated aza-acetal structure at the proline motif, which was previous unpresented in the brevianamide analogues. Thus, the planar structure of **1** was determined as shown in Figure 1.

In order to determine the relative configuration of **1**, the rotating frame Overhauser effect spectroscopy (ROESY, Figure S5) experiment was performed. The ROESY correlation (Figure 2B) between NH-2 (*δ*_H_ 9.01) and H-13 (*δ*_H_ 7.29) revealed the *Z* configuration about *Δ*^3,10^, and the ROESY signals between H-8β (*δ*_H_ 2.13) and H-6, and between H-8α (*δ*_H_ 2.27) and H-9 suggested that H-6 and H-9 were *trans* form. The absolute configuration of **1** was assigned as (6*R*,9*S*) by comparing the experimental and calculated electronic circular dichroism (ECD) values obtained using Time-dependent Density functional theory (TD-DFT) at the B3LYP/6–31+g (d, p) level (Figure 2C).

**Figure 3:**
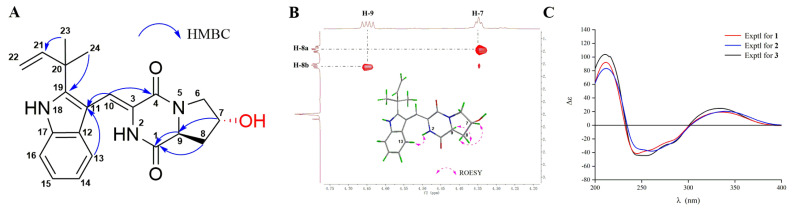
The key HMBC correlations (**A**) and partial enlarged view of ROESY spectra (**B**) of **3**, and experimental ECD spectra (**C**) of compounds **1–3**.

Aspamide B (**2**) was obtained as a yellowish powder. Its molecular formula was determined as C_23_H_27_N_3_O_3_ on the basis of HRESIMS (*m*/*z* 394.2120 [M + H]^+^, calcd. for C_23_H_28_N_3_O_3_, 394.2125) and ^13^C NMR data, corresponding to 12 degrees of unsaturation. By comparing the ^1^H, ^13^C, and HMQC data (Table 1, Figure S11) of **2** with those of **1**, it was discovered that **2** possessed the identical planar structure as that of **1**. Further analyses of the 2D NMR data of **2**, the key HMBC correlations from H-6 (*δ*_H_ 5.35) to OCH_2_CH_3_-6 (*δ*_C_ 64.3) and C-9 (*δ*_C_ 58.7), as well as H-9 (*δ*_H_ 4.44) to C-1 (*δ*_C_ 166.8) revealed that the oxethyl group was located at C-6. Furthermore, the difference between H-6 (*δ*_H_ 5.35, dd, *J* = 2.8, 1.7 Hz) in **2** and H-6 (*δ*_H_ 5.59, dd, *J* = 5.7, 1.7 Hz) in **1** indicated that **2** was the C-6 epimer of **1**. Furthermore, the same ECD cotton effects (Figure 3C) of **2** compared to **1** indicated that the absolute configuration of C-9 in **2** was consistent with that in **1**. This result suggested that within the used spectral window, the ECD cotton effects were mainly caused by the chiral center of C-9 in both compounds **1** and **2**, and it could also be confirmed by the experimental ECD data of (+)-brevianamide V and (−)-brevianamide V [35]. Thus, the absolute configuration of compound **2** was ascertained as (6*S*,9*S*).

Aspamide C (**3**) was obtained as a yellowish powder. The molecular formula was established as C_21_H_23_N_3_O_3_ by HRESIMS (*m*/*z* 366.1807 [M + H]^+^, calcd. for C_21_H_24_N_3_O_3_, 366.1812), indicating 12 degrees of unsaturation. The UV and NMR spectra were very similar to those of compound **1**. A comparison of the NMR data for **3** with **1**, together with characteristic HMBC signals (Figure 3A), suggested that the oxethyl group was replaced by a second OH in **3**. However, the OH group was not located at C-6, which was the same as the **1**, to form the aza-acetal structure for which the chemical shift of oxygenated methylene (*δ*_C_ 66.6) was far below the shift of C-6 (*δ*_C_ 86.7) in **1**. Thus, the second OH was distributed to C-7, and the planar structure of **3** was determined as shown in Figure 1. The ROESY correlation (Figure S20) between NH-2 (*δ*_H_ 8.93) and H-13 (*δ*_H_ 7.29) confirmed the *cis* form of the double bond between C-3 and C-10. Furthermore, the ROESY signals (Figure 3B) between H-8β (*δ*_H_ 1.98) and H-7 (*δ*_H_ 4.35), and between H-8α (*δ*_H_ 2.12) and H-9 (*δ*_H_ 4.65) revealed that H-7 and H-9 were *trans* form. Finally, the absolute configuration of **3** was determined as (7*R*,9*S*) by comparison of the experimental ECD curve of **3** with that of **1** (Figure 3C).

**Figure 4:**
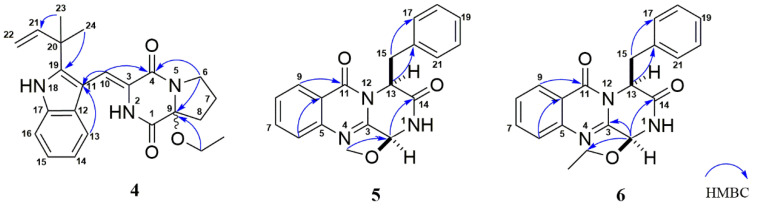
The key HMBC correlations of compounds **4**–**6**.

**Figure 5:**
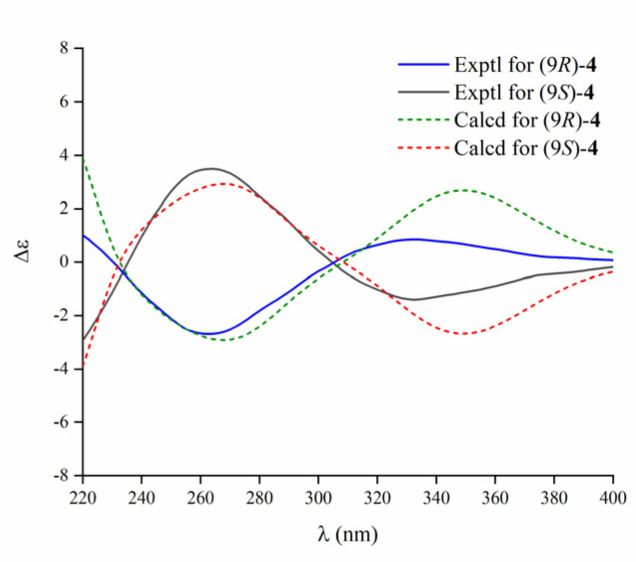
Experimental and calculated ECD spectra of **4**.

The racemic (±)-aspamide D (**4**) was isolated as colorless gum with the molecular formula of C_23_H_27_N_3_O_3_ from an HRESIMS peak at *m*/*z* 394.2120 [M + H]^+^ (calcd. for C_23_H_28_N_3_O_3_, 394.2125). Its NMR data and UV absorption were similar to compound **1**. Comparing to **1**, the major change was that two sp^3^ methines were replaced by one sp^3^ methylene and one sp^3^ non-protonated carbon, suggesting that the oxethyl group was connected to C-9 rather to the C-6. Additionally, the key HMBC signals (Figure 4) from OCH_2_CH_3_-9 (*δ*_H_ 3.53) to C-9 (*δ*_C_ 91.0), H-6 (*δ*_H_ 3.62) to C-4 (*δ*_C_ 159.4) and C-9, and H-8a (*δ*_H_ 2.02) to C-1 (*δ*_C_ 163.1) confirmed the aforementioned planar structure. There was no Cotton effect observed on its ECD spectra (Figure S29), which in accordance with the racemic (±)-brevianamide X [35], indicating that **4** might be a pair of enantiomers. Furthermore, the chiral HPLC resolution of **4** contributed to the separation of a pair of enantiomers (+)-**4** and (−)-**4**, which exhibited nearly mirror-image ECD spectra (Figure 5). The absolute configurations of (+)-**4** and (−)-**4** were discriminably determined as 9*R* and 9*S* by comparing the experimental and calculated ECD data obtained using TD-DFT at the B3LYP/6-31+g (d, p) level (Figure 5). Correspondingly, we named (+)-**4** and (−)-**4** as (+)-aspamide D and (−)-aspamide D, respectively.

**Figure 6:**
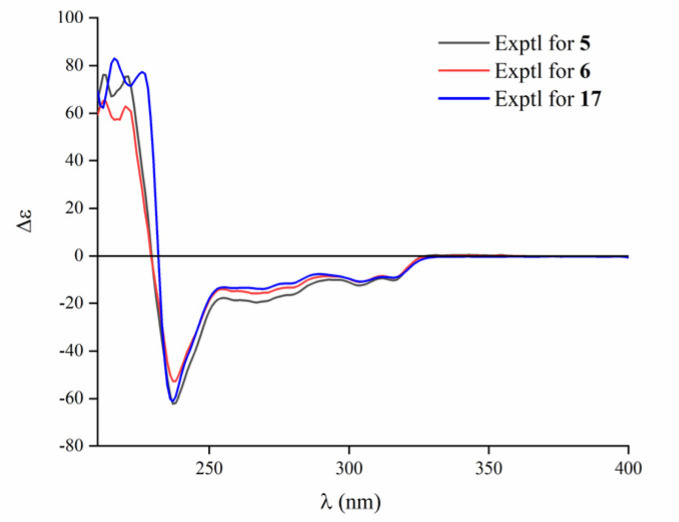
Experimental ECD spectra of **5**, **6**, and **17**.

Aspamide F (**5**) was obtained as a brown powder with the molecular formula of C_19_H_17_N_3_O_3_ from an HRESIMS peak at *m*/*z* 336.1336 [M + H]^+^ (calcd. for C_19_H_18_N_3_O_3_, 336.1343), requiring 13 indices of hydrogen deficiency. The ^1^H NMR, ^13^C NMR, and HMQC spectra (Table 2 and Figure S35) suggested the presence of one oxygenated methyl group, one sp^3^ methylene, two sp^3^ methines carbon signals (including one oxygen-bearing carbon), nine sp^2^ methines, and six sp^2^ non-protonated carbons. These NMR data of **5** were similar to those of brevianamide M [28], except for the different chemical shifts for C-2 and C-3 due to the presence of a methoxy at C-2 in **5**, implying that **5** was an analogue of brevianamide M with a methoxy at C-2. Additionally, the key HMBC correlations (Figure 4) from OCH_3_-2 (*δ*_H_ 3.53) to C-2 (*δ*_C_ 83.9), from H-2 (*δ*_H_ 5.27) to C-3 (*δ*_C_ 146.9)/C-14 (*δ*_C_ 170.0), and from H-13 (*δ*_H_ 5.53) to C-15 (*δ*_C_ 40.0)/C-16 (*δ*_C_ 135.9) confirmed the planner structure of **5** as shown in Figure 4. The absolute configuration of **5** was determined as (2*S*,13*S*) via comparing the ECD curve (Figure 6) of **5** with the brevianamide M (**17**).

Aspamide G (**6**) was isolated as a brown powder. The molecular formula was determined as C_20_H_19_N_3_O_3_ by HRESIMS (*m*/*z* 350.1498 [M + H]^+^, calcd. for C_20_H_20_N_3_O_3_, 350.1499), which was 14 Dalton more than **5**. The ^13^C NMR data of **6** showed a close resemblance to those of **5**, except for an additional oxygenated sp^3^ methylene, suggesting that there was an ethoxy group located at C-2 in **6**. The key HMBC signal (Figure 4) from H-2 (*δ*_H_ 6.40) to OCH_2_-2 (*δ*_C_ 66.2) verified that **6** was an analogue of brevianamide M (**17**) with an ethoxy motif at C-2. In addition, similar Cotton effects at 212, 220, and 237 nm in the ECD spectra (Figure 6) of **6** suggested that **6** and **17** had the same counterpart absolute configurations. Thus, the absolute configuration of **6** was assigned as (2*S*,13*S*), and it was elucidated as 6-ethoxy-aspamide F.

All isolated compounds were tested for their anti-inflammatory activities in *P. acnes*-induced THP-1 cells; unfortunately, none of the compounds showed moderate anti-inflammatory properties. Aiming to give our contribution to the COVID-19 research, all compounds were selected for the virtual screening on the 3CL hydrolase (Mpro) of SARS-CoV-2, which had been exploited as a potential drug target to fight COVID-19 [27]. The docking scores of compounds **1–2**, **5**, **6**, **8** and **17** were top among all screened molecules (docking scores: −5.389, −4.772, −5.146, −4.962, −5.158), and the score of ritonavir [36] (a potent inhibitor in vitro of human immunodeficiency virus type 1 protease) was −7.039, which suggested that these compounds may be helpful in fighting COVID-19 after further studies.

## 3. Materials and Methods

### 3.1. General Experimental Procedures

Optical rotations were recorded on a JASCO P-1020 polarimeter (JASCO Corporation, Tokyo, Japan) in MeOH at 25 °C. UV spectra were measured using a Shimadzu UV-1800 spectrophotometer (Shimadzu Corporation, Tokyo, Japan). High-resolution electrospray ionization (HR-ESI-MS) were obtained with an Agilent 6529B Q-TOF instrument (Agilent Technologies, Santa Clara, CA, USA). ECD spectra were carried out with Chirascan circular dichroism spectrometers (Applied Photophysics Ltd., Leatherhead, UK). Both 1D and 2D NMR spectra were acquired on a Bruker AVIII-400 and Bruker AVIII-600 NMR spectrometers with tetramethylsilane (TMS) as an internal standard (Bruker, Karlsruhe, Germany). Preparative high-performance liquid chromatography (Pre-HPLC) was performed utilizing a Shimadzu LC-20 system (Shimadzu, Tokyo, Japan) equipped with a Shim-pack RP-C18 column (20 × 250 mm i.d., 10 µm, Shimadzu, Tokyo, Japan) with a flow rate at 10 mL/min at 25 °C, which was recorded by a binary channel UV detector at 210 nm and 254 nm. The analytical chiral HPLC used was a JASCO LC-2000 system equipped with a Daicel Chiralpak AD-H column (4.6 mm × 250 mm, 5 μm) and a CD-2095 chiral detector at 280 nm. The mobile phase was *n*-hexane/isopropanol (80:20, *v*/*v*) used at a flow rate of 0.5 mL/min. Column chromatography (CC) was performed with silica gel (200–300 mesh, Qingdao Marine Chemical Inc., Qingdao, China) and ODS (50 µm, YMC, Kyoto, Japan) on a Flash Chromatograph System (SepaBen machine, Santai Technologies, Changzhou, China). Thin-layer chromatography (TLC) was performed using precoated silica gel GF254 plates (Qingdao Marine Chemical Inc., Qingdao, China).

### 3.2. Fungal Material

The endophytic DY180635 was isolated from a sample of crab (*Chiromantes haematocheir*), which was collected from the intertidal zone of Zhoushan, Zhejiang, China, in June 2018. It was incubated on a potato dextrose agar (PDA) plate at 28 °C. The strain DY180635 was identified using ITS rDNA sequence analysis by RuiDi (Shanghai, China) and its DNA sequence using BLAST was compared to the GenBank data. The result of BLAST searching was closest to that of *Aspergillus versicolor* NRRL 238 (GenBank accession number NG_067623) with 99% sequence identity. The nucleotide sequences of the ITS gene (accession number MT361076) of *A*. *versicolor* DY180635 were deposited in GenBank. A reference culture is stored in State Key Laboratory of Bioreactor Engineering laboratory of Shanghai at −80 °C.

### 3.3. Fermentation, Extraction, and Isolation

The fungus was incubated on potato dextrose agar (PDA) medium at 28 °C for approximately 2–3 days; then it was cut into three agar pieces (nearly the size of 0.5 × 0.5 × 0.5 cm) and transferred into a 500 mL Erlenmeyer flask, containing 200 mL of potato dextrose broth (PDB). The flasks were cultured for 2 days at 28 °C on a rotary shaker at 180 rpm for inoculation. The seed cultures were added to the 200 × 1 L flasks containing rice medium (110 g rice, 120 mL deionized water), which was previously sterilized at 121 °C for 25 min. All flasks were incubated at 28 °C for four weeks.

Following incubation, the solid rice cultures were extracted three times by EtOAc to give a crude extract (489.0 g); the crude extract was suspended in water and then partitioned with EtOAc to give an EtOAc soluble fraction (185.2 g) after the solvent was removed to dryness under reduced pressure. The EtOAc fraction was further separated on macroporous adsorbent resin with a stepped gradient elution with EtOH-H_2_O (30, 50, 70 and 100%). The 50% fraction was sequentially separated by silica gel with petroleum ether-EtOAc (5:1 to 0:1) to give four subfractions (A**–**D) using the TLC. The subfraction B (9.0 g) was sequentially loaded onto silica gel CC (petroleum ether-EtOAc, 5:1) and preparative HPLC (MeCN-H_2_O, 50:50, 10.0 mL/min) to yield compounds **1** (10.4 mg, *t*_R_ 34.9 min), **2** (6.4 mg, *t*_R_ 27.1 min), **4** (5.9 mg, *t*_R_ 33.5 min), and **6** (8.7 mg, *t*_R_ 23.2 min). Subfraction C (15.0 g) was further separated by an ODS column (MeCN-H_2_O, 40:60) and repeated preparative HPLC with MeCN-H_2_O (50:50, 10 mL/min) to give compounds **3** (4.6 mg, *t*_R_ 8.8 min) and **5** (4.7 mg, *t*_R_ 17.1 min).

Aspamide A (**1**): yellowish powder; ${\left[\alpha \right]}_{\text{D}}^{25}$ + 120.0 (*c* 0.05, MeOH); ECD (5 mg/L, MeOH) *λ*_max_ (Δε) 212 (92.18), 245 (−41.48) 335 (19.29) nm; UV (MeOH) *λ*_max_ (log*ε*) 200 (6.13), 224 (6.13), 284 (5.46), 341 (5.68) nm; ^1^H and ^13^C NMR (DMSO-*d*_6_), see Table 1; positive HR-ESI-MS *m*/*z* 394.2117 [M + H] ^+^, (calcd. for C_23_H_28_N_3_O_3_, 394.2125).

Aspamide B (**2**): yellowish powder; ${\left[\alpha \right]}_{\text{D}}^{25}$ + 112.0 (*c* 0.05, MeOH); ECD (5 mg/L, MeOH) *λ*_max_ (Δε) 212 (83.31), 261 (−37.91), 340 (20.41) nm; UV (MeOH) *λ*_max_ (log*ε*) 200 (6.18), 224 (6.18), 283 (5.45), 345 (5.73) nm; ^1^H and ^13^C NMR (DMSO-*d_6_*), see Table 1; positive HR-ESI-MS *m*/*z* 394.2120 [M + H]^+^, (calcd. for C_23_H_28_N_3_O_3_, 394.2125).

Aspamide C (**3**): yellowish powder; ${\left[\alpha \right]}_{\text{D}}^{25}$ + 125.0 (*c* 0.05, MeOH); ECD (5 mg/L, MeOH) *λ*_max_ (Δε) 211 (103.75), 255 (−44.38), 334 (24.79) nm; UV (MeOH) *λ*_max_ (log*ε*) 200 (6.20), 224 (6.19), 284 (5.56), 337 (5.75) nm; ^1^H and ^13^C NMR (DMSO-*d_6_*), see Table 1; positive HR-ESI-MS *m*/*z* 366.1807 [M + H]^+^, (calcd. for C_21_H_24_N_3_O_3_, 366.1812).

(±)-Aspamide D (**4**): colorless gum; ${\left[\alpha \right]}_{\text{D}}^{25}$ + 5.0 (*c* 0.05, MeOH); UV (MeOH) *λ*_max_ (log*ε*) 200 (6.07), 224 (6.06), 283 (5.33), 346 (5.60) nm; ^1^H and ^13^C NMR (DMSO-*d_6_*), see Table 2; positive HR-ESI-MS *m*/*z* 394.2122 [M + H]^+^, (calcd. for C_23_H_28_N_3_O_3_, 394.2125).

Aspamide F (**5**): brown powder; ${\left[\alpha \right]}_{\text{D}}^{25}$ + 40.0 (*c* 0.05, MeOH); ECD (5 mg/L, MeOH) *λ*_max_ (Δε) 212 (65.33), 220 (62.98), 237 (−52.79) nm; UV (MeOH) *λ*_max_ (log*ε*) 206 (5.95), 224 (6.15), 270 (5.25), 278 (5.23) nm; ^1^H and ^13^C NMR (CDCl_3_), see Table 2; positive HR-ESI-MS *m*/*z* 336.1336 [M + H]^+^, (calcd. for C_19_H_18_N_3_O_3_, 336.1343).

Aspamide G (**6**): brown powder; ${\left[\alpha \right]}_{\text{D}}^{25}$ + 56.0 (*c* 0.05, MeOH); ECD (5 mg/L, MeOH) *λ*_max_ (Δε) 212 (76.04), 221 (75.42), 237 (-62.18) nm; UV (MeOH) *λ*_max_ (log*ε*) 205 (5.92), 222 (5.88), 270 (5.16), 278 (5.13) nm; ^1^H and ^13^C NMR (CDCl_3_), see Table 2; positive HR-ESI-MS *m*/*z* 350.1498 [M + H]^+^, (calcd. for C_20_H_20_N_3_O_3_, 350.1499).

### 3.4. ECD Calculation

The relative configuration of **1** was established initially according to its ROESY NMR spectra. Monte Carlo conformational searches were carried out by means of the Spartan’s 14 software using the Merck molecular force field (MMFF). The conformers with Boltzmann population of over 1% were chosen for ECD calculations, and then the conformers were initially optimized at the B3LYP/6-31G(d,p) level in gas. The theoretical calculation of ECD was conducted in MeOH using Time-dependent Density functional theory (TD-DFT) at the B3LYP/6-31+G(d,p) level for all conformers. Rotatory strengths for a total of 100 excited states were calculated. ECD spectra were generated using the program SpecDis 1.6 (University of Würzburg, Würzburg, Germany) and GraphPad Prism 5 (University of California San Diego, USA) from dipole-length rotational strengths by applying Gaussian band shapes with sigma = 0.3 eV. And UV-shift values of all configurations were −10 nm. The spectra of enantiomers were produced directly by mirror inversions.

### 3.5. Virtual Screening Against COVID-19 Main Protease

#### 3.5.1. Protein and Ligand Preparation

The 3CL hydrolase (Mpro) of SARS-CoV-2 had been exploited as a potential drug target to fight COVID-19 [27]. Thus, the virtual screening was conducted by using the SARS-CoV-2 enzyme (PDB ID 6LU7) obtained from the Protein Data Bank (PDB, http://www.rcsb.org/pdb), and the structure was optimized by using the protein preparation wizard module in Maestro software package (Schrodinger LLC, NY, USA). Specifically, the water and heteroatom were removed, the polar hydrogens were added to the protein to the protonation state, the entire structure was assumed as a neutral pH, and the energy of the structure was minimized by using an OPLS2005 force field. The docking grid of 20 Å size was generated over the co-crystallized ligand. All compounds 1–17 were implemented by Ligprep software, and the structure energy was minimized using OPL2005 force field.

#### 3.5.2. Virtual Screening

Virtual screening was performed by the Schrodinger glide docking module, and the standard-precision (SP) docking was designated to get accurate results [37]. The results were measured by docking score, and only the compounds with scores in the top half were subjected to the extra-precision (XP) docking.

### 3.6. Cell Culture and Cell Viability Assay

The human monocytic cell line, THP-1 (Cell Bank of China Science Academy, Shanghai, China) and *P*. *acnes* (ATCC6919, Xiangfu biotech, Shanghai, China), were used for the anti-inflammatory assay. THP-1 cells were cultured in RPMI1640 medium with 10% fetal bovine serum (FBS, Gibco, NY, USA) in a humidified incubator (37 °C, 5% CO_2_). *P*. *acnes* bacteria were incubated in Cooked Meat Medium, containing cooked beef granules (Rishui biotechnology, Qingdao, China) in an anaerobic environment. The THP-1 cells were stimulated by the *P*. *acnes*, which was harvested at the exponential phase. The viability of THP-1 cells was evaluated by the MTT assay, specifically, seeding the THP-1 cells in 96-well plates at a density of 2 × 10^5^ cells/well and treated with serially diluted compounds for 36 h (37 °C, 5% CO_2_). After that, we added 20 µL MTT regent (5 mg/mL, Genetimes Technology Inc., Shanghai, China) to each well and incubated the samples at 37 °C for 4 h. Removing the supernatant, the formazan crystals were fully solubilized in DMSO (150 µL), and the absorbance was measured at 570 nm and 630 nm.

## 4. Conclusions

Chemical investigation of a marine-derived fungus *Aspergillus versicolor* DY180635 led to the isolation and identification of four new IDPKs, aspamides A–E (**1–4**) and two new DPKs, aspamides F–G (**5–6**), along with 11 known diketopiperazines and intermediates. Further chiral high-performance liquid chromatography resolution gave enantiomers (+)- and (−)-**4**, respectively. The structures and absolute configurations of compounds **1–6** were determined by the comprehensive analyses of NMR, HRESIMS, and ECD calculation. Compounds **1–17** were selected for the virtual screening on the 3CL hydrolase (Mpro) of SARS-CoV-2, and compounds **1–2**, **5**, **6**, **8** and **17** possessed top docking scores and thus may be helpful in fighting COVID-19 after further studies.

## Figures and Tables

**Table 1:** ^1^H (600 MHz) and ^13^C (150 MHz) NMR data of **1**–**3** in DMSO-*d*_6_.

No.		1		2		3
	*δ*_C_	*δ*_H_, Mult. (*J* in Hz)	*δ*_C_	*δ*_H_, Mult. (*J* in Hz)	*δ*_C_	*δ*_H_, Mult. (*J* in Hz)
1	165.9	-	166.8	-	166.2	-
2	-	9.01, s	-	9.43, s	-	8.93, s
3	125.3	-	125.9	-	126.2	-
4	158.9	-	161.1	-	158.4	-
6a	86.7	5.59, dd (5.7, 1.7)	85.4	5.35, dd (2.8, 1.7)	54.4	3.32, m
6b	-	-	-	-	-	3.75, dd (12.7, 4.5)
7a	29.6	1.75, dddd (13.4, 8.4, 5.0, 1.7)	30.7	1.92, m	66.6	4.35, t (4.5)
7b		1.97, m		-		
8a	25.9	2.13, dddd (12.2, 9.7, 6.7, 5.0)	24.5	1.92, m	37.6	1.98, ddd (12.7, 11.5, 4.5)
8b		2.27, m		2.10, m		2.12, dd (12.7, 6.0)
9	56.5	4.57, dd (8.9, 6.7)	58.7	4.44, m	57.0	4.65, dd (11.5, 6.0)
10	111.9	6.98, s	113.2	7.05, s	110.7	6.92, s
11	103.9	-	104.3	-	103.8	-
12	125.9	-	125.8	-	126.0	-
13	119.6	7.29, d (8.0)	119.7	7.23, d (8.0)	119.4	7.29, d (8.0)
14	119.4	6.99, m	119.3	6.99, ddd (8.0, 7.0, 1.2)	119.3	7.00, m
15	120.7	7.08, ddd (8.0, 7.0, 1.2)	120.7	7.07, ddd (8.0, 7.0, 1.2)	120.7	7.08, ddd (8.0, 7.0, 1.2)
16	111.5	7.41, d (8.0)	111.6	7.41, m	111.4	7.41, d (8.0)
17	135.1	-	135.1	-	135.1	-
18		11.06, s	-	11.06, s	-	11.03, s
19	144.3	-	144.7	-	144.0	-
20	39.0	-	39.1	-	39.0	-
21	145.1	6.07, dd (17.4, 10.5)	145.1	6.09, dd (17.1, 10.8)	145.2	6.08, dd (17.3, 10.5)
22	111.7	5.05, m	111.7	5.06, m	111.6	5.04, m
23	27.4	1.45, s	27.5	1.47, s	27.4	1.46, s
24	27.8	1.49, s	27.9	1.51, s	27.7	1.50, s
6-OEt	63.6	3.65, m	64.3	3.60, m	-	-
	15.2	1.13, t (7.1)	15.4	1.09, t (7.1)	-	-

**Table 2:** ^1^H (600 MHz) and ^13^C (150 MHz) NMR data of **4**–**6** (**4** in DMSO-*d_6_*, **5**–**6** in CDCl_3_).

No.		4		5		6
	*δ*	*δ*_H_, Mult. (*J* in Hz)	*δ*_C_	*δ*_H_, Mult. (*J* in Hz)	*δ*_C_	*δ*_H_, Mult. (*J* in Hz)
1	163.1	-	-	-	-	-
2	-	9.43, s	83.9	5.27, d (4.7)	78.3	6.40, d (4.3)
3	125.5	-	146.9	-	151.1	-
4	159.4	-	-	-		-
5	-	-	146.7	-	139.0	-
6	45.1	3.62, dd (8.6, 5.8)	127.8	7.74, d (8.0)	122.9	8.23, d, (8.0)
7a	19.5	1.92, m	134.9	7.79, t (8.0)	136.7	7.91, t (8.0)
7b		1.96, m				
8a	32.4	2.02, m	128.0	7.53, t (8.0)	129.7	7.66, t (8.0)
8b		2.33, m				
9	91.0	-	127.0	8.24, d (8.0)	127.9	8.23, d, (8.0)
10	112.7	7.02, s	120.8	-	119.1	-
11	103.9	-	160.3	-	157.4	-
12	126.3	-	-	-	-	-
13	119.1	7.21, d (8.0)	57.4	5.53, dd (8.8, 6.5)	58.1	5.54, t (7.8)
14	119.0	7.00, m	170.0	-	167.6	-
15	120.7	7.09, m	40.0	3.43, m	39.9	3.48, m
16	111.6	7.43, d (8.0)	135.9	-	134.9	-
17	135.1	-	129.8	7.29, m	129.7	7.24, m
18		11.10, s	128.6	7.28, m	128.8	7.24, m
19	144.4	-	127.0	7.24, m	127.7	7.20, m
20	39.0	-	128.6	7.28, m	128.8	7.24, m
21	145.2	6.09, ddd (17.1, 10.8, 1.6)	129.8	7.29, m	129.7	7.24, m
22	111.7	5.06, m	-	-	-	-
23	27.4	1.47, s	-	-	-	-
24	27.8	1.50, s	-	-	-	-
9-OEt	59.2	3.53, qd (7.0, 3.9)	-	-	-	-
-	15.2	1.22, td (7.0, 1.5)	-	-	-	-
2-OMe/ 2-OEt	-	-	56.0	3.53, s	66.2	4.04, dt (9.0, 7.0)
						4.11, dt (9.0, 7.0)
	-	-	-	-	15.4	1.35, t (7.0)

## Supplementary Materials

The following are available online at: https://www.mdpi.com/1660-3397/18/7/338/s1, Figures S1–S46: ^1^H, ^13^C, HSQC, HMBC, ROESY, UV, ECD, and HRESIMS spectra of the new compounds **1**–**6**, Tables S1–S6: Computational data of **1** and **4**.
