# Supplementary material for: Bioactive Indolyl Diketopiperazines from the Marine Derived Endophytic Aspergillus versicolor DY180635
Source: Mar Drugs. 2020 Jun 28;18(7):338. doi: 10.3390/md18070338 (PMC7401283; doi:10.3390/md18070338)
Supplement: Supplementary file 1 [file marinedrugs-18-00338-s001.pdf]

# Bioactive Indolyl Diketopiperazines from the Marine Derived Endophytic *Aspergillus versicolor* DY180635

Yi Ding, Xiaojing Zhu, LiLing Hao, MengYao Zhao, Qiang Hua, and Faliang An *

State Key Laboratory of Bioreactor Engineering, East China University of Science and Technology, 130 Mei Long Road, Shanghai 200237, People's Republic of China

* Corresponding author: E-mail address: flan2016@ecust.edu.cn; Tel.: +86-21-6425-1185 (F.A.).

# Supporting Information

# Table of contents


**Figure S1.** $^1$H NMR spectra (600 MHz, DMSO-$d_6$) of **1**......4
**Figure S2.** $^{13}$C NMR spectra (150 MHz, DMSO-$d_6$) of **1**......5
**Figure S3.** HMQC spectra (DMSO-$d_6$) of **1**......5
**Figure S4.** HMBC spectra (DMSO-$d_6$) of **1**......6
**Figure S5.** ROESY spectra (DMSO-$d_6$) of **1**......7
**Figure S6.** UV spectra (MeOH) of **1**......7
**Figure S7.** ECD spectra (MeOH) of **1**......7
**Figure S8.** HRESIMS spectra of **1**......8
**Figure S9.** $^1$H NMR spectra (600 MHz, DMSO-$d_6$) of **2**......8
**Figure S10.** $^{13}$C NMR spectra (150 MHz, DMSO-$d_6$) of **2**......9
**Figure S11.** HMQC spectra (DMSO-$d_6$) of **2**......9
**Figure S12.** HMBC spectra (DMSO-$d_6$) of **2**......10
**Figure S13.** UV spectra (MeOH) of **2**......10
**Figure S14.** ECD spectra (MeOH) of **2**......11
**Figure S15.** HRESIMS spectra of **2**......11
**Figure S16.** $^1$H NMR spectra (600 MHz, DMSO-$d_6$) of **3**......12
**Figure S17.** $^{13}$C NMR spectra (150 MHz, DMSO-$d_6$) of **3**......12
**Figure S18.** HMQC spectra (DMSO-$d_6$) of **3**......13
**Figure S19.** HMBC spectra (DMSO-$d_6$) of **3**......13
**Figure S20.** ROESY spectra (DMSO-$d_6$) of **3**......14
**Figure S21.** UV spectra (MeOH) of **3**......14
**Figure S22.** ECD spectra (MeOH) of **3**......15
**Figure S23.** HRESIMS spectra of **3**......15
**Figure S24.** $^1$H NMR spectra (600 MHz, DMSO-$d_6$) of **4**......16
**Figure S25.** $^{13}$C NMR spectra (150 MHz, DMSO-$d_6$) of **4**......16
**Figure S26.** HMQC spectra (DMSO-$d_6$) of **4**......17
**Figure S27.** HMBC spectra (DMSO-$d_6$) of **4**......17
**Figure S28.** UV spectra (MeOH) of **4**......18
**Figure S29.** ECD spectra (MeOH) of **4**......18
**Figure S30.** ECD spectra (MeOH) of (+) - **4**......19
**Figure S31.** ECD spectra (MeOH) of (-) - **4**......19
**Figure S32.** HRESIMS spectra of **4**......20
**Figure S33.** $^1$H NMR spectra (600 MHz, $CDCl_3$) of **5**......20
**Figure S34.** $^{13}$C NMR spectra (150 MHz, $CDCl_3$) of **5**......21

**Figure S35.** HMQC spectra ($CDCl_3$) of **5.** ......................................................................................21
**Figure S36.** HMBC spectra ($CDCl_3$) of **5**. ......................................................................................22
**Figure S37.** UV spectra (MeOH) of **5**. ..............................................................................................22
**Figure S38.** ECD spectra (MeOH) of **5**...............................................................................................23
**Figure S39.** HRESIMS spectra of **5**. ..................................................................................................23
**Figure S40.** $^{1}$H NMR spectra (600 MHz, $CDCl_3$) of **6**......................................................................24
**Figure S41.** $^{13}$C NMR spectra (150 MHz, $CDCl_3$) of **6**. ....................................................................24
**Figure S42.** HMQC spectra ($CDCl_3$) of **6**. .....................................................................................25
**Figure S43.** HMBC spectra ($CDCl_3$) of **6**. ......................................................................................25
**Figure S44.** UV spectra (MeOH) of **6**. ..............................................................................................26
**Figure S45.** ECD spectra (MeOH) of **6**...............................................................................................26
**Figure S46.** HRESIMS spectra of **6**. ..................................................................................................27
**Table S1.** Energies of conformers of **1** with Boltzmann distribution over 1% at MMFF.......................27
**Table S2.** Gibbs free energies[a] and equilibrium populations[b] of low-energy conformers of **1**. .............27
**Table S3.** Cartesian coordinates for the low-energy reoptimized MMFF conformers of **1** at B3LYP/6-31+G (d, p) level of theory in gas. ..............................................................................................28
**Table S4.** Energies of conformers of **4b** with Boltzmann distribution over 1% at MMFF. ...................45
**Table S5.** Gibbs free energies[a] and equilibrium populations[b] of low-energy conformers of **4b**. ...........45
**Table S6** Cartesian coordinates for the low-energy reoptimized MMFF conformers of **4b** at B3LYP/6-31+G (d, p) level of theory in gas. ..............................................................................................46
**Table S5.** Gibbs free energies[a] and equilibrium populations[b] of low-energy conformers of (-)-**4**. **Error! Bookmark not defined.**
**Table S6.** Cartesian coordinates for the low-energy reoptimized MMFF conformers of (-)-**4** at B3LYP/6-31+G (d, p) level of theory in $CH_3OH$. ..................................................**Error! Bookmark not defined.**

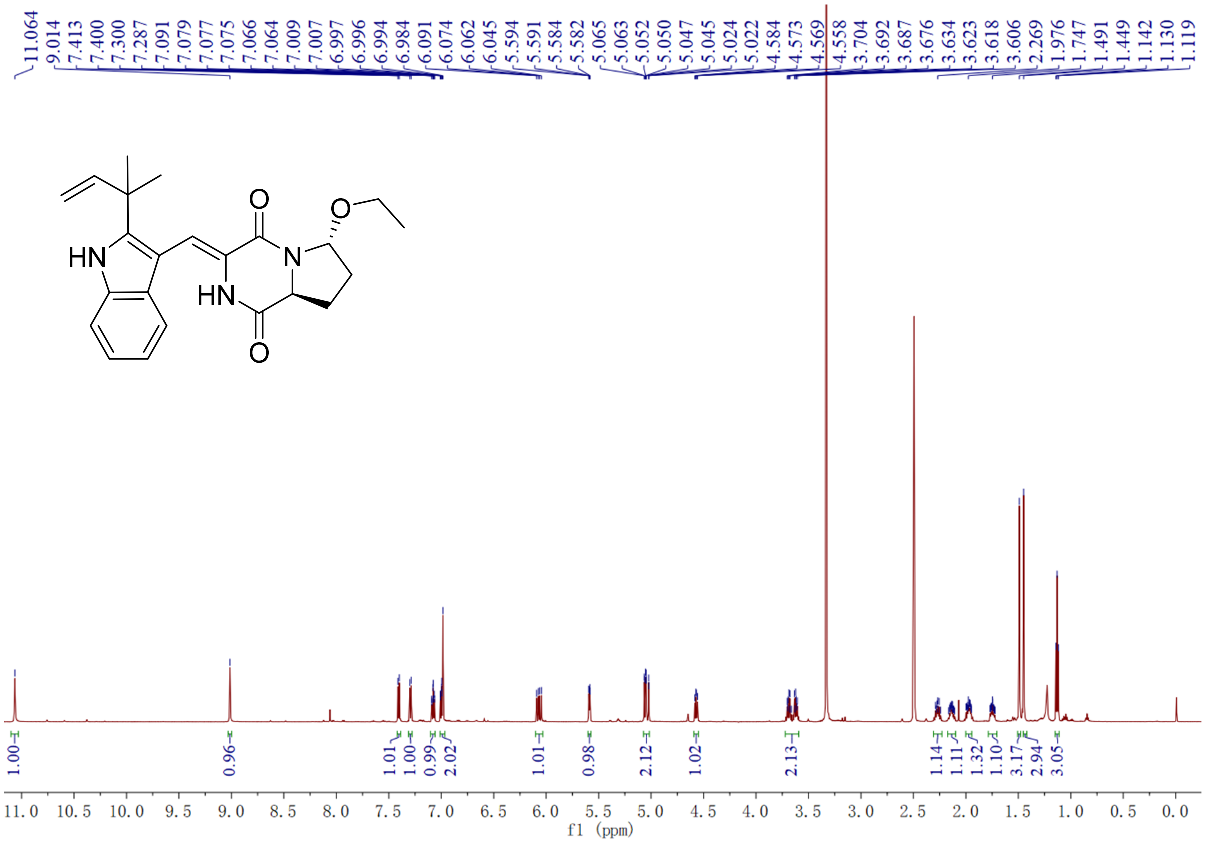


**Figure S1.** $^{1}$H NMR spectra (600 MHz, DMSO-$d_6$) of **1**.

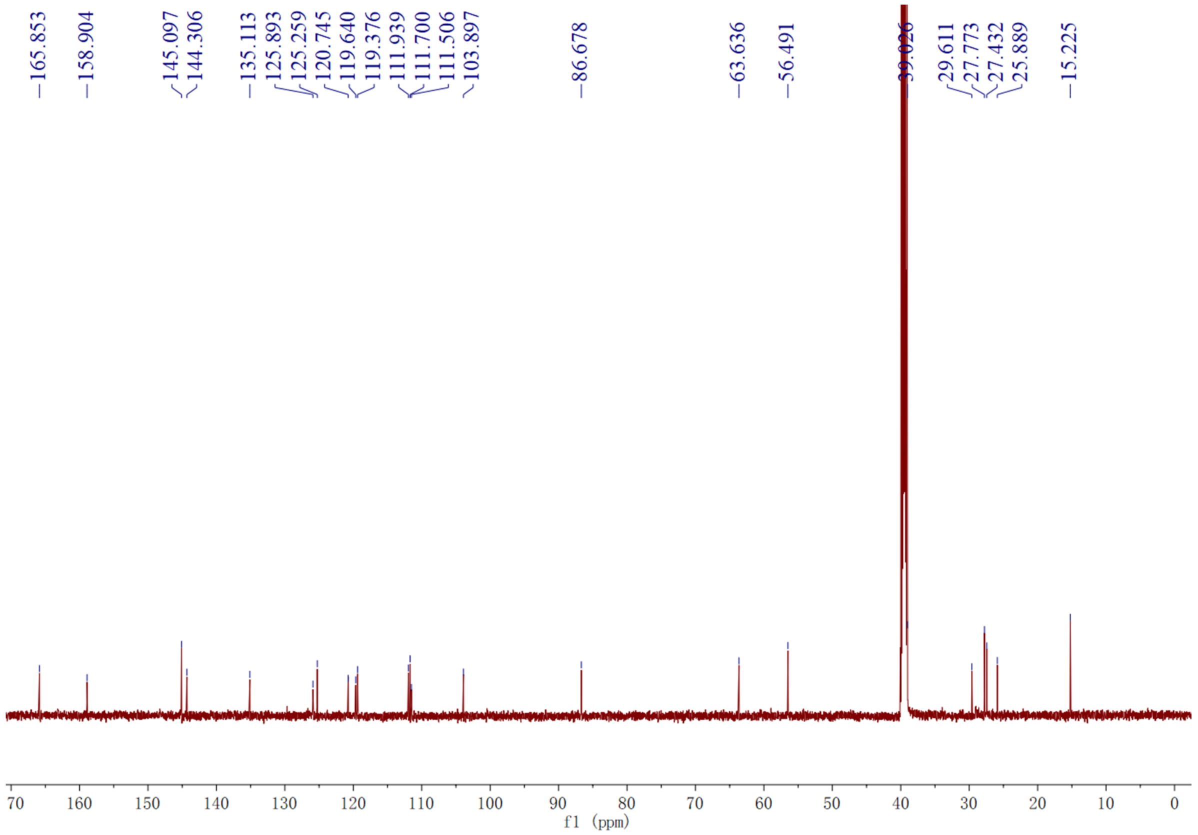


**Figure S2.** $^{13}$C NMR spectra (150 MHz, DMSO-$d_6$) of **1**.

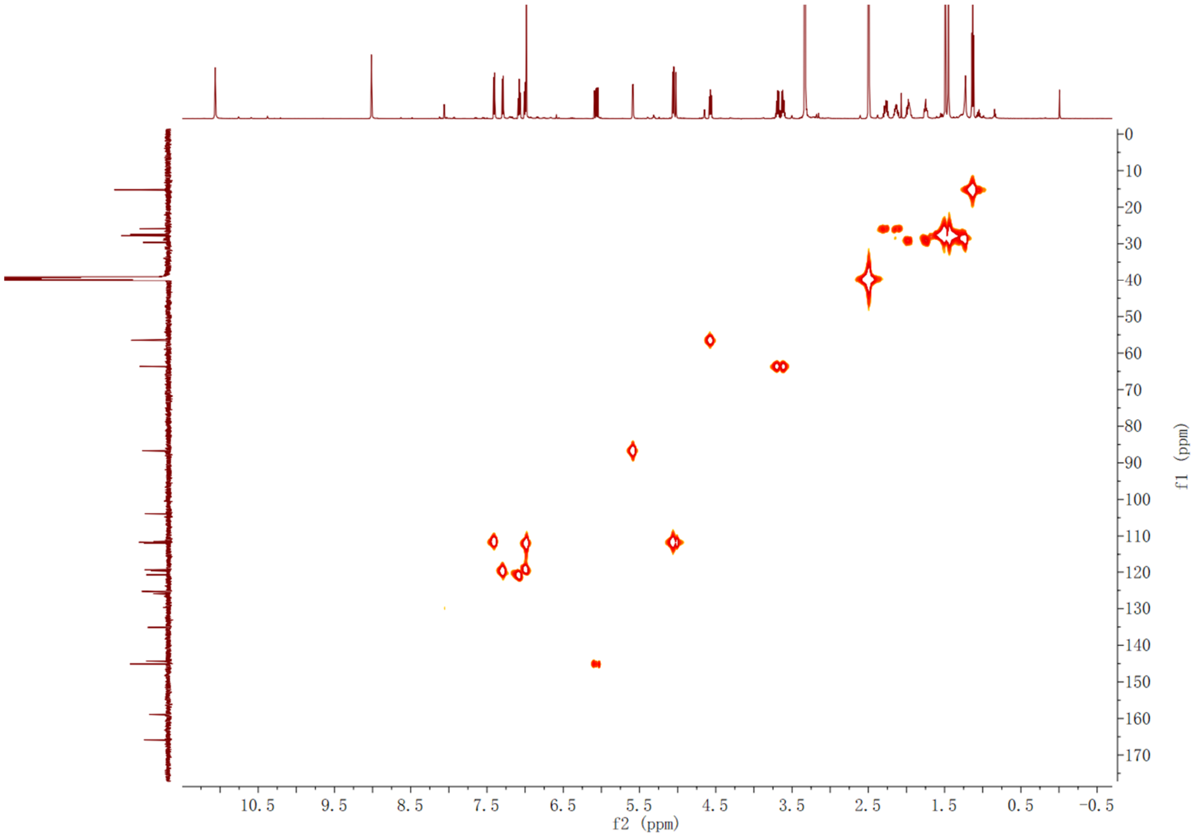


**Figure S3.** HMQC spectra (DMSO-$d_6$) of **1**.

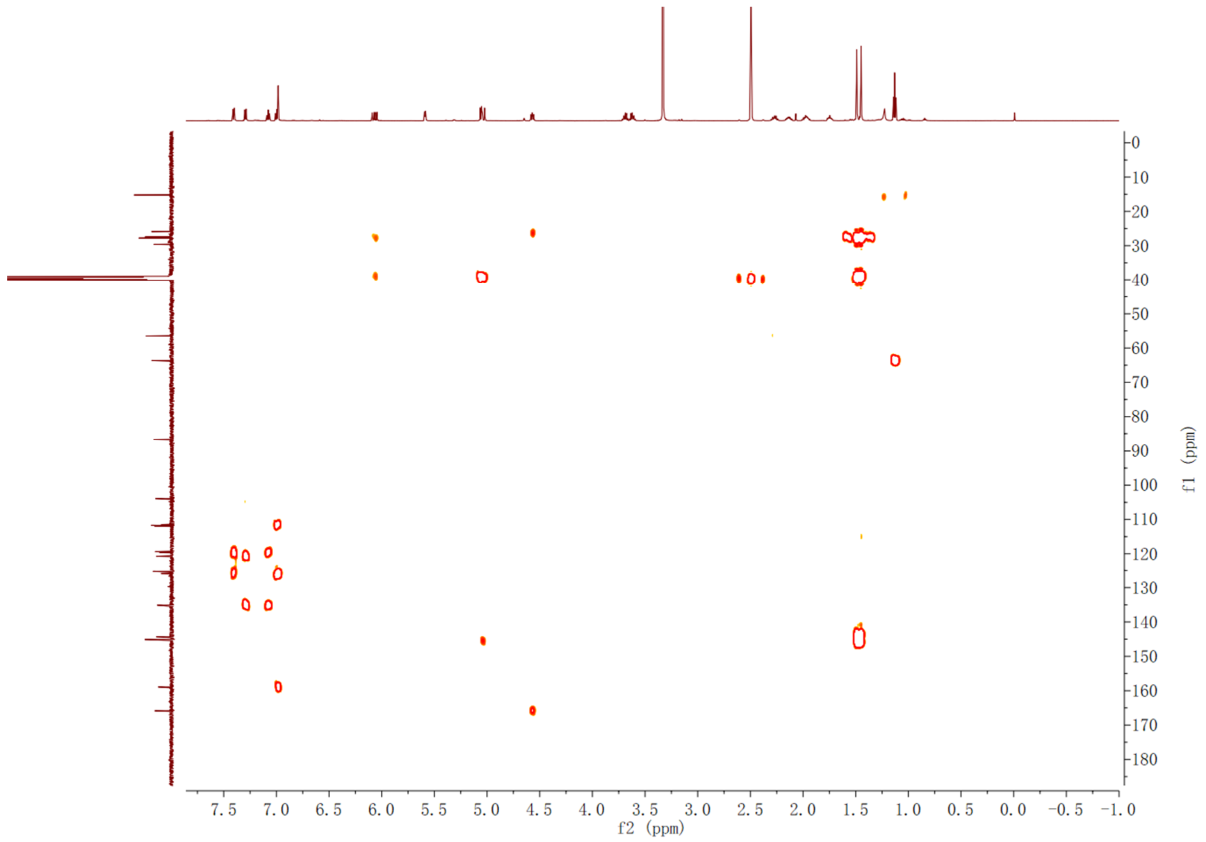


**Figure S4.** HMBC spectra (DMSO-$d_6$) of **1**.

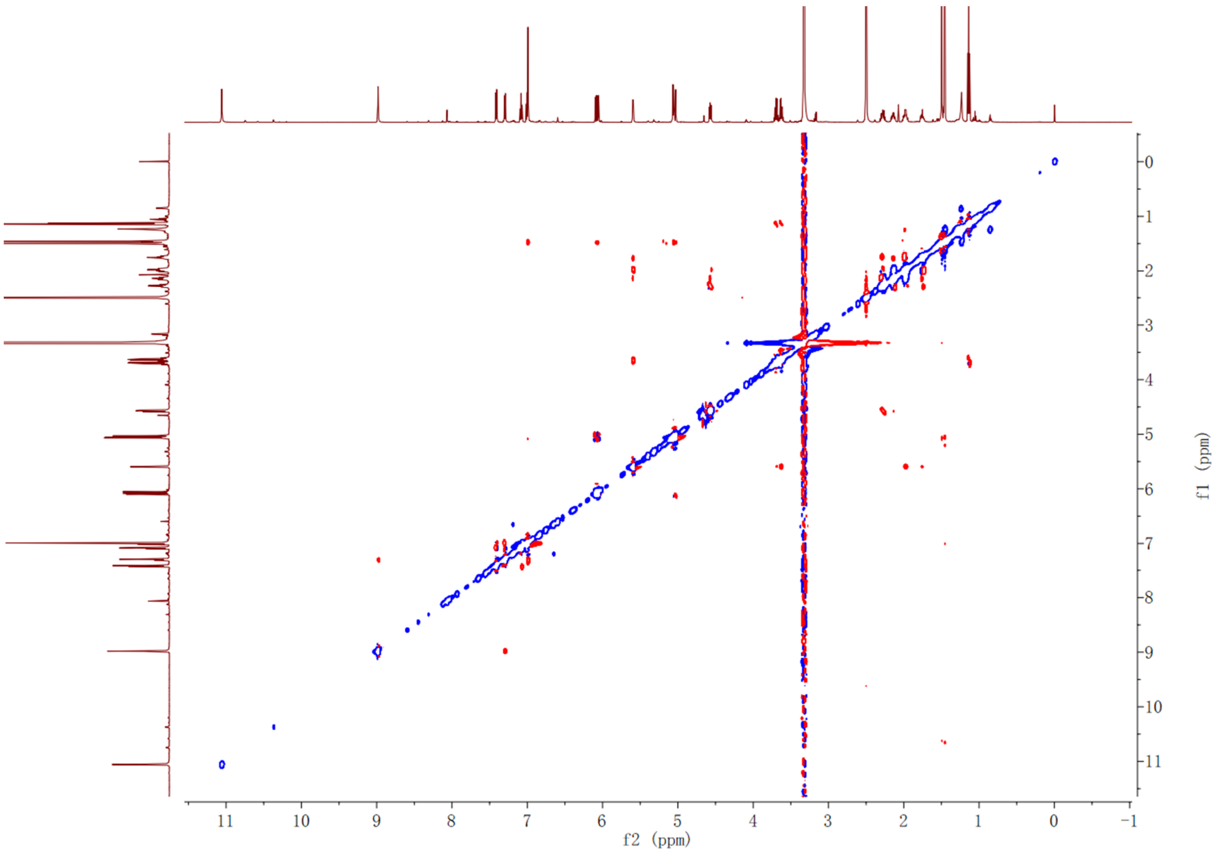

**Figure S5.** ROESY spectra (DMSO-$d_6$) of **1**.

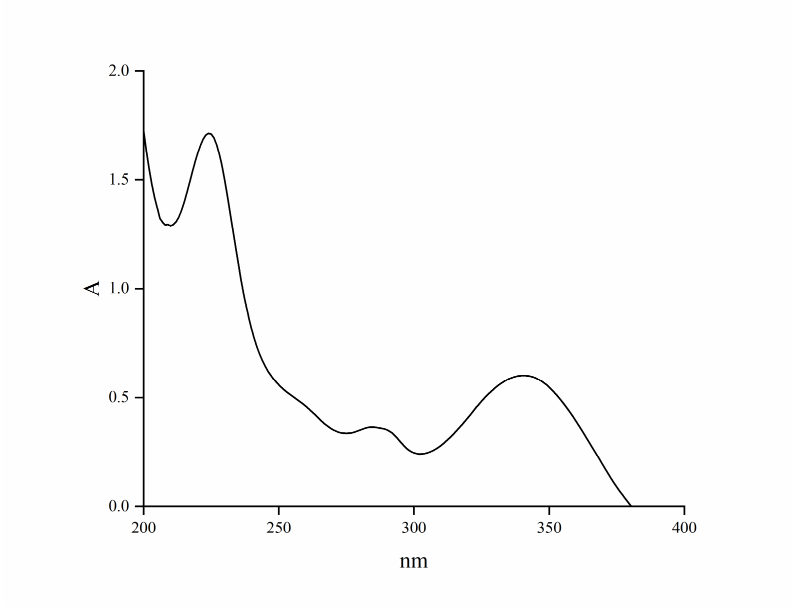


**Figure S6.** UV spectra (MeOH) of **1**.

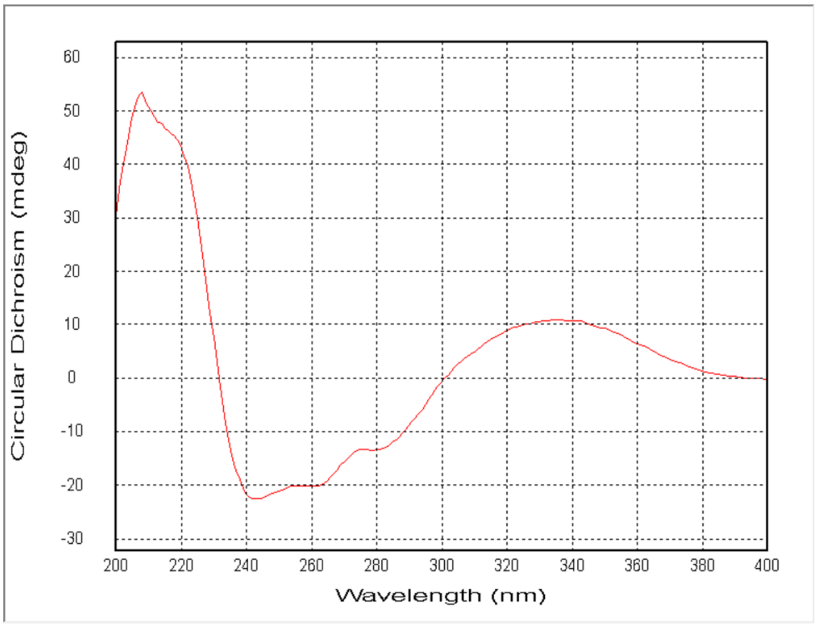


**Figure S7.** ECD spectra (MeOH) of **1**.

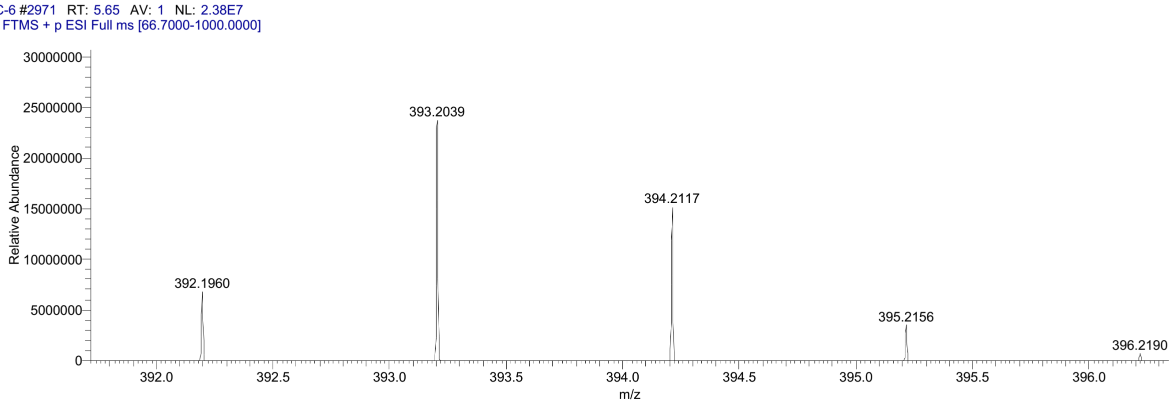


**Figure S8.** HRESIMS spectra of **1**.

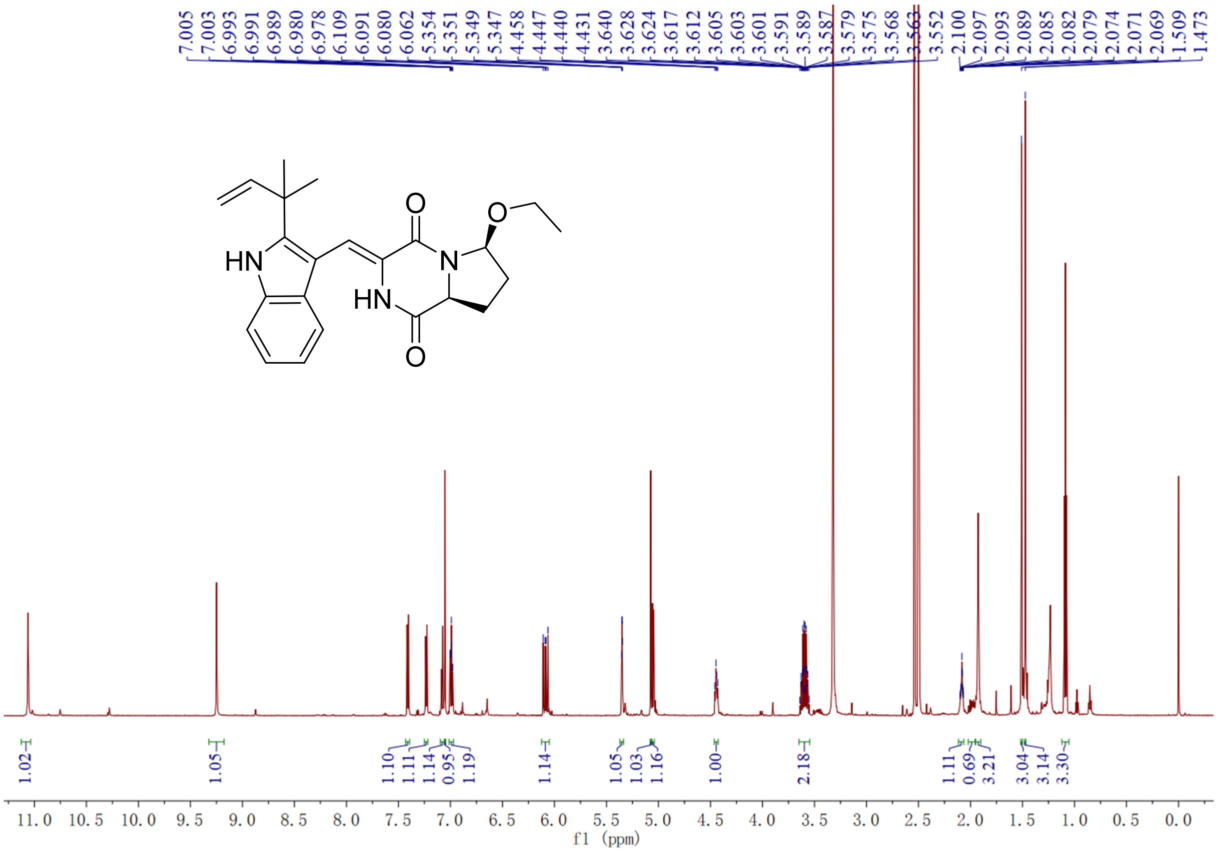


**Figure S9.** $^{1}$H NMR spectra (600 MHz, DMSO-$d_6$) of **2**.

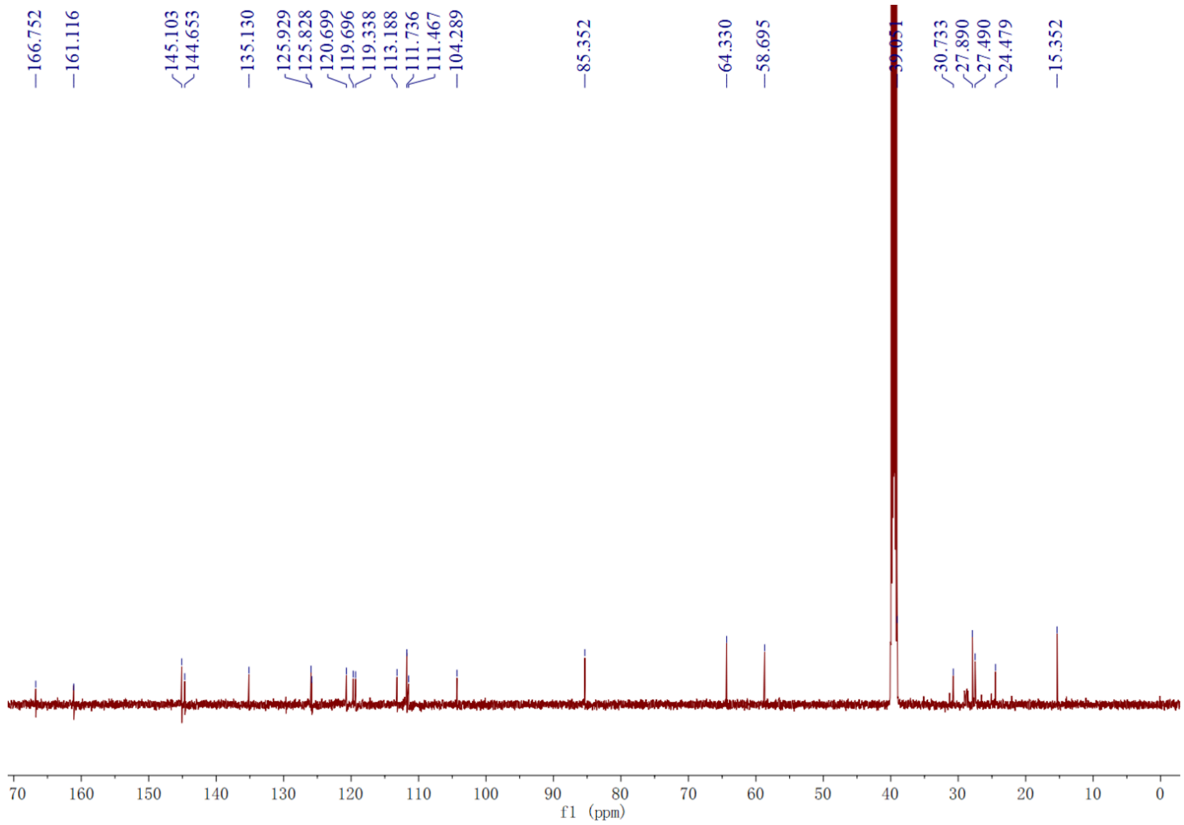


**Figure S10.** $^{13}C$ NMR spectra (150 MHz, DMSO-$d_6$) of **2**.

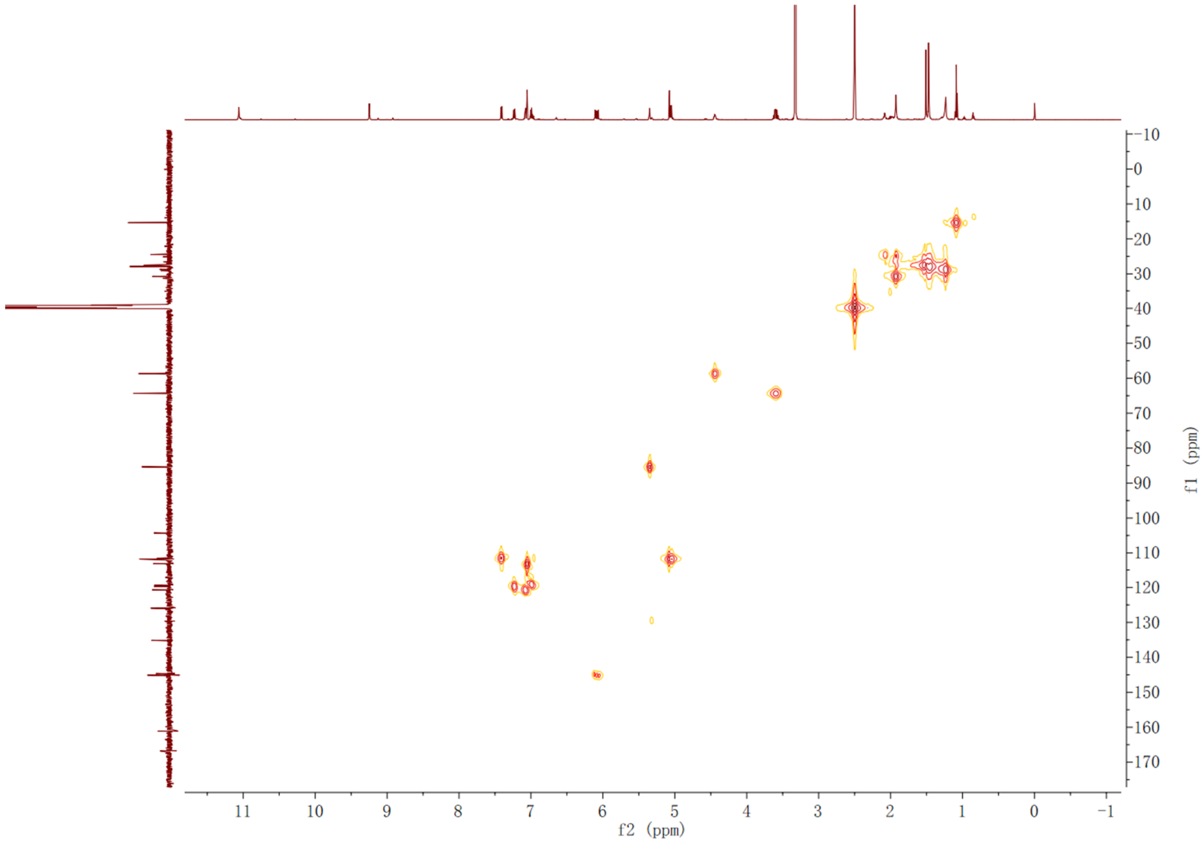


**Figure S11.** HMQC spectra (DMSO-$d_6$) of **2**.

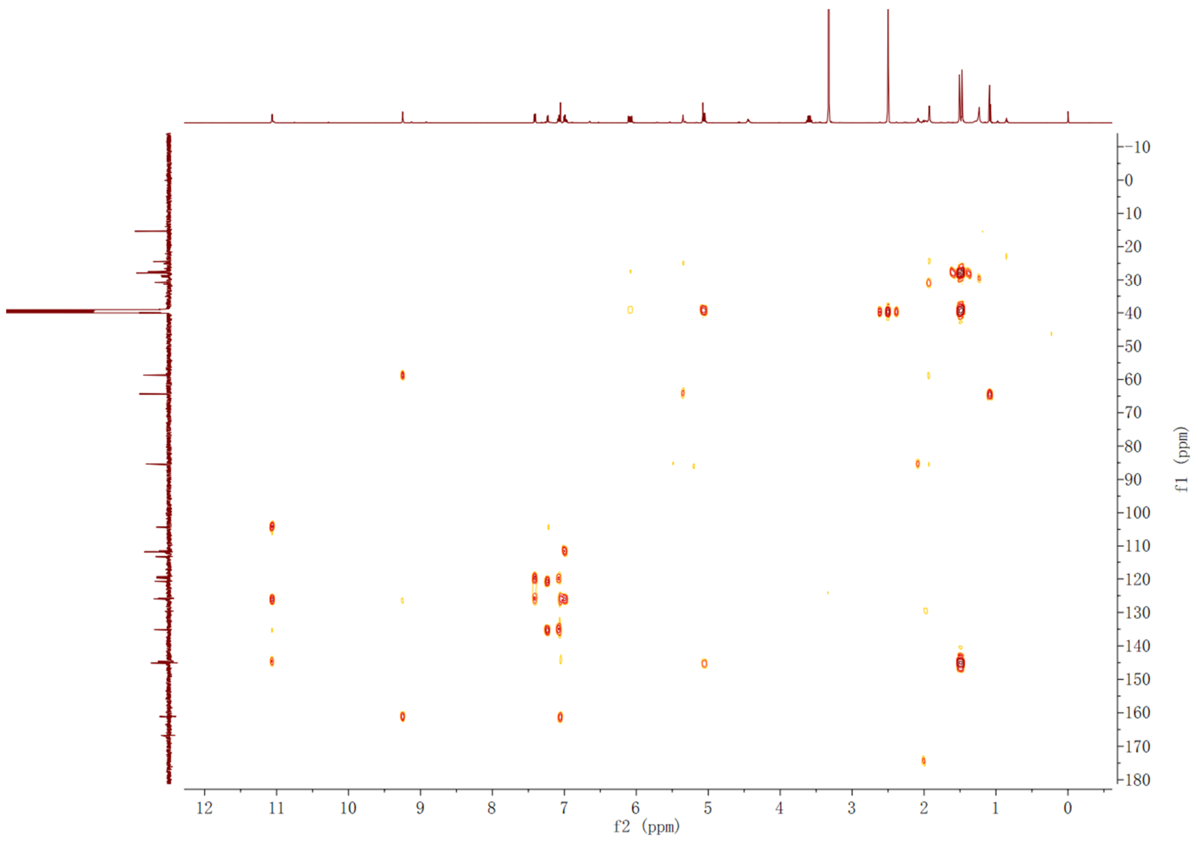


**Figure S12.** HMBC spectra (DMSO-$d_6$) of **2**.

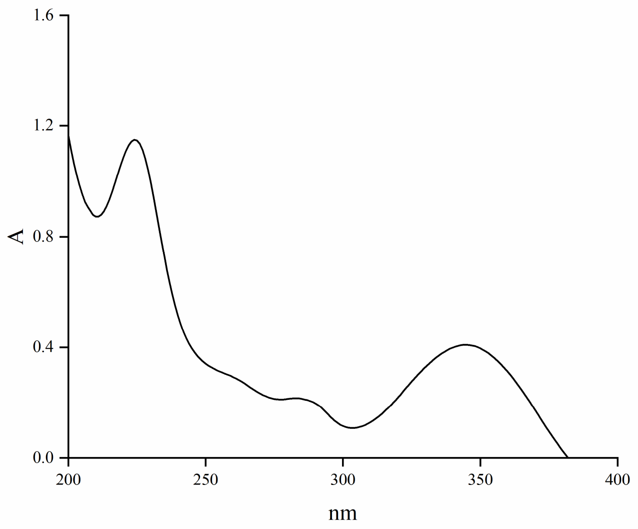


**Figure S13.** UV spectra (MeOH) of **2**.

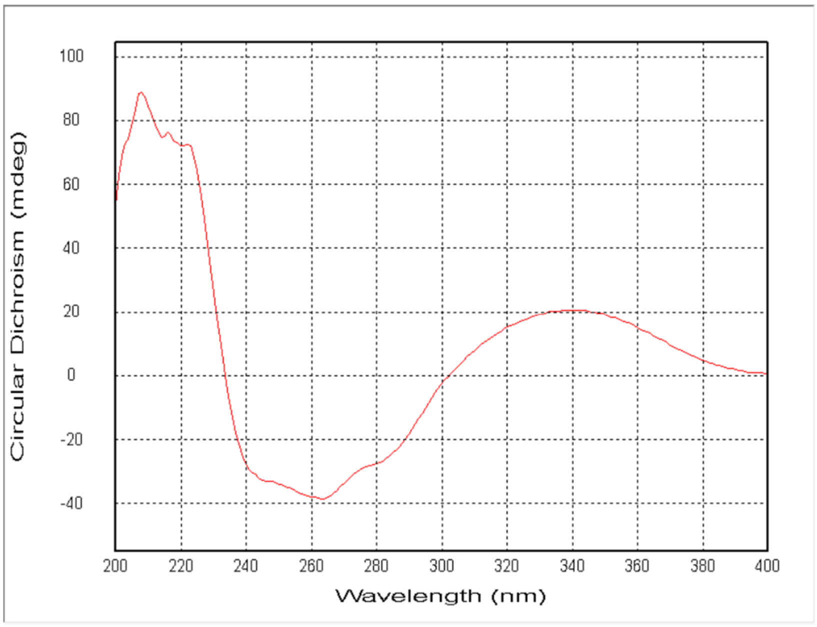


**Figure S14.** ECD spectra (MeOH) of **2**.

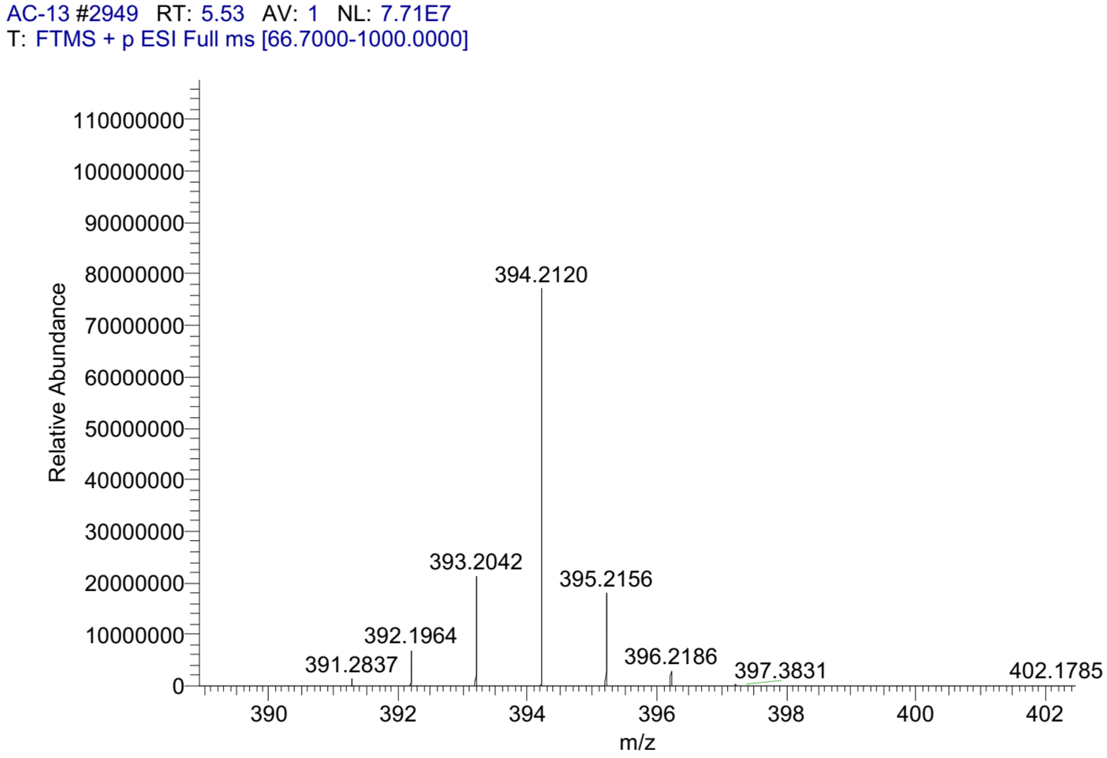


**Figure S15.** HRESIMS spectra of **2**.

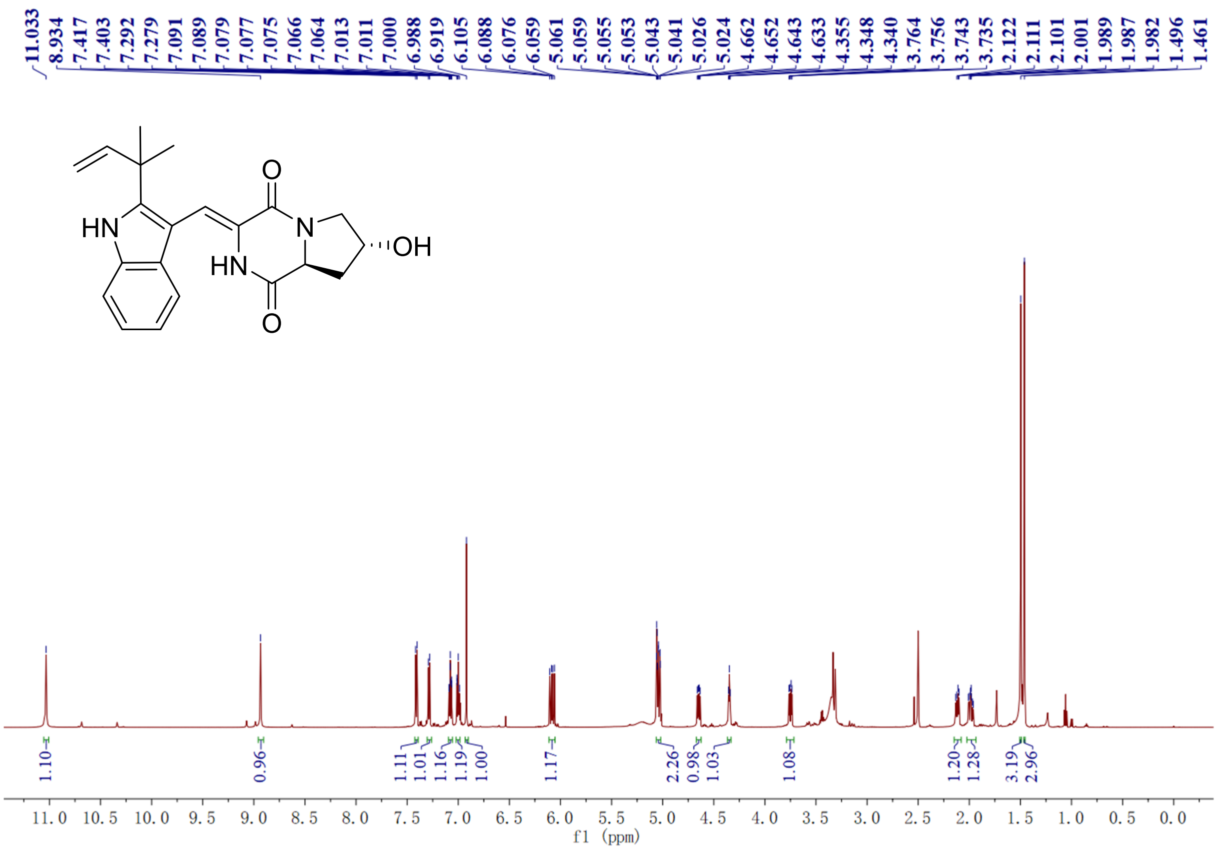


**Figure S16.** $^{1}$H NMR spectra (600 MHz, DMSO-$d_6$) of **3**.

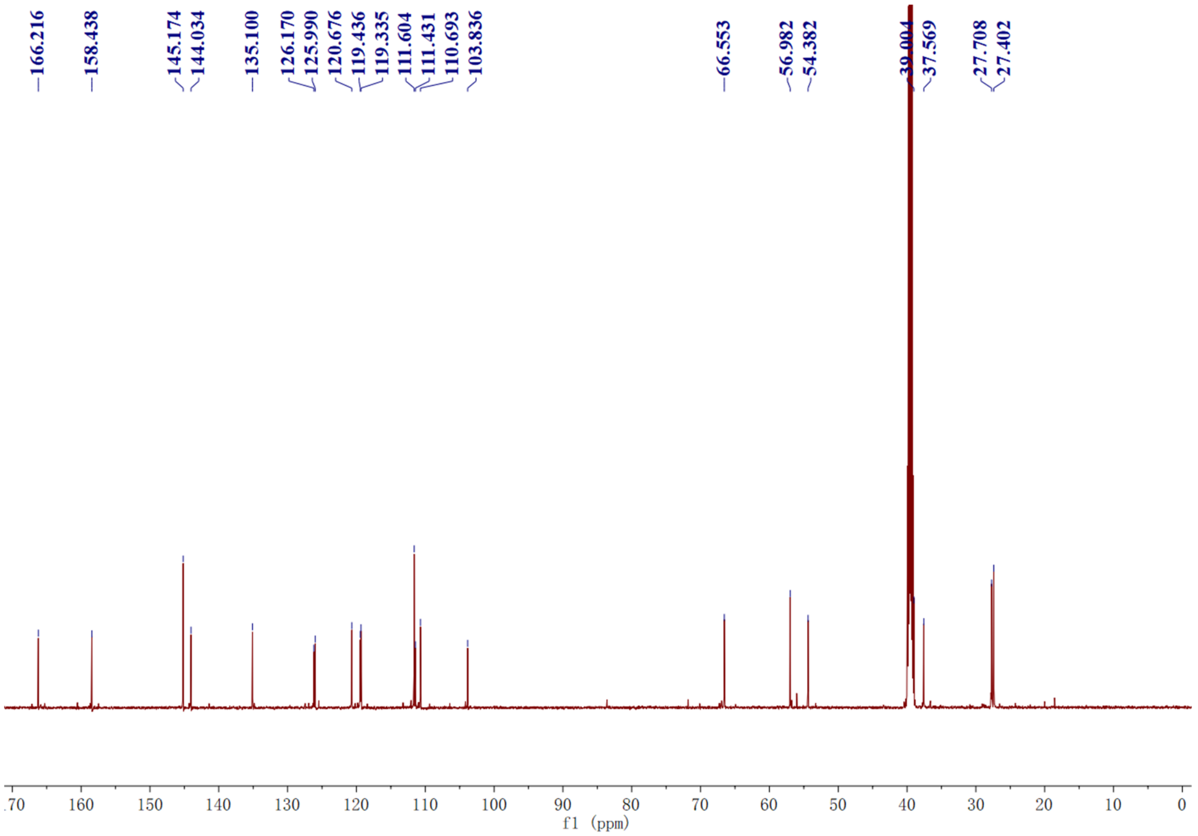


**Figure S17.** $^{13}$C NMR spectra (150 MHz, DMSO-$d_6$) of **3**.

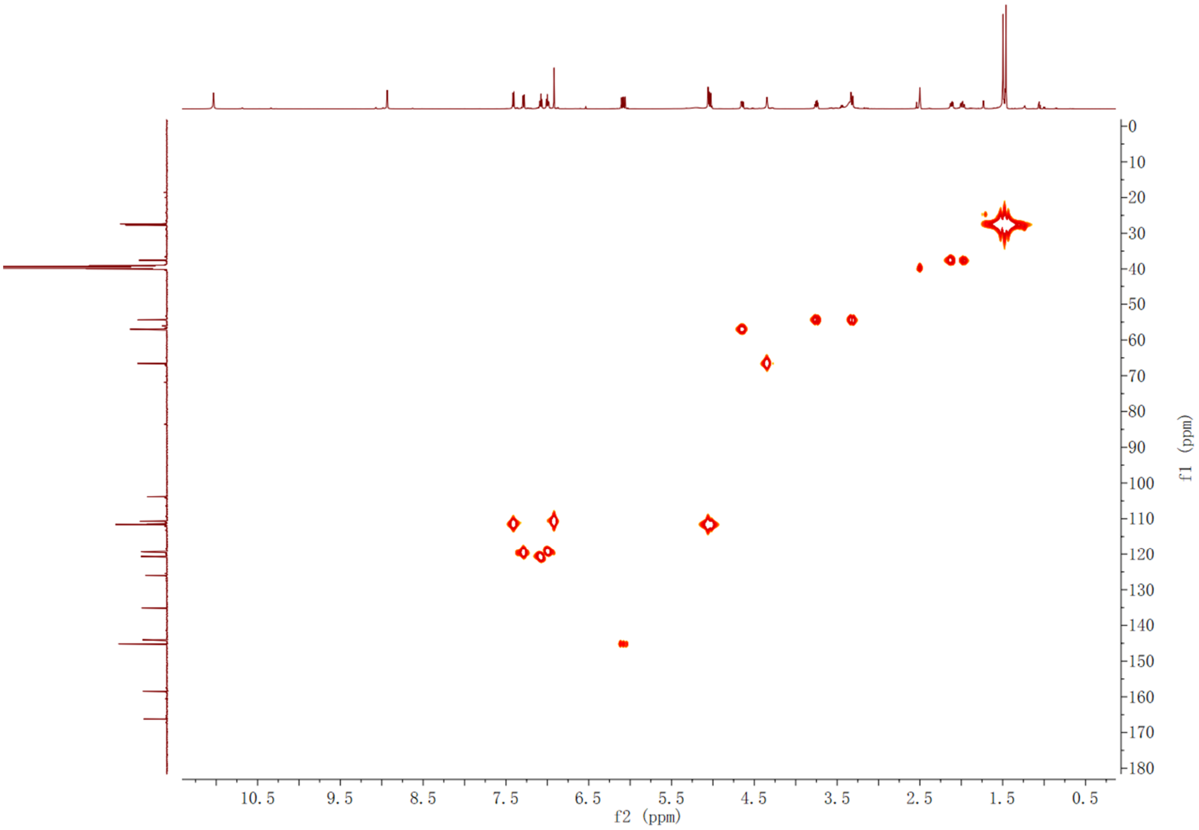


**Figure S18.** HMQC spectra (DMSO-$d_6$) of **3**.

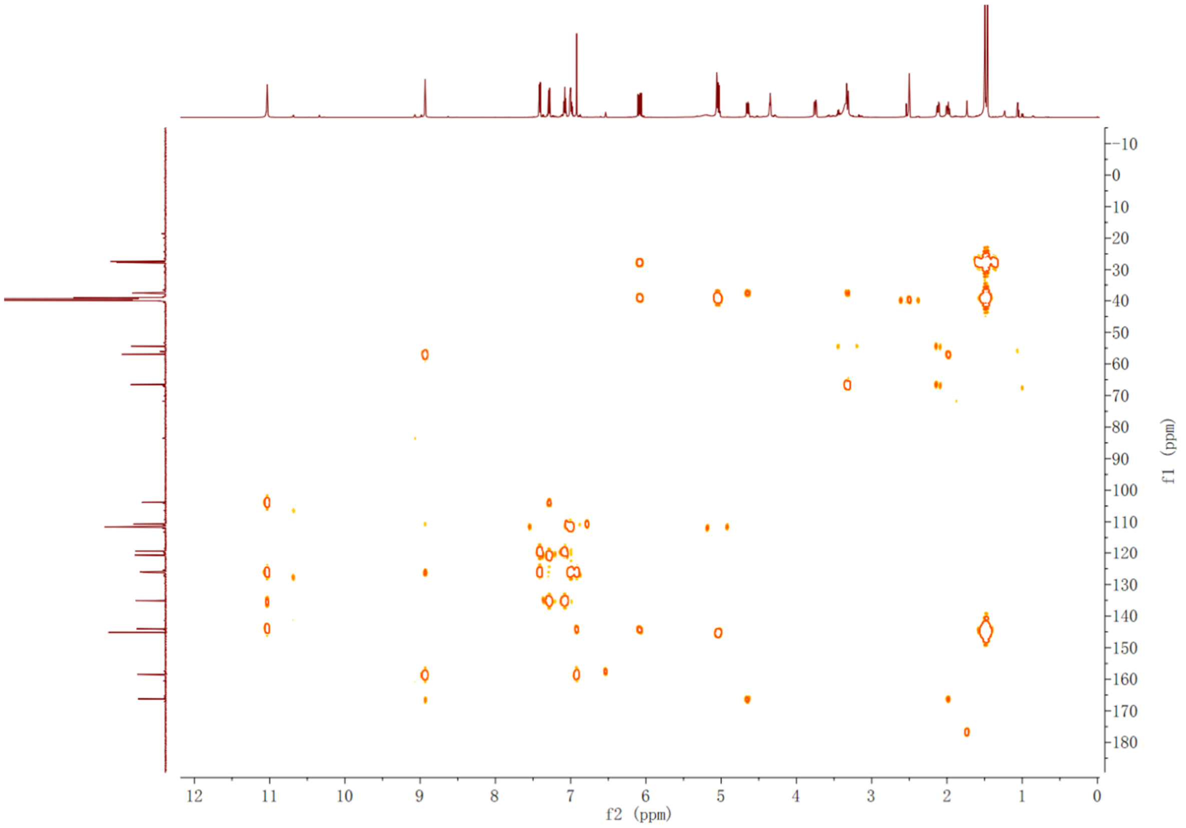


**Figure S19.** HMBC spectra (DMSO-$d_6$) of **3**.

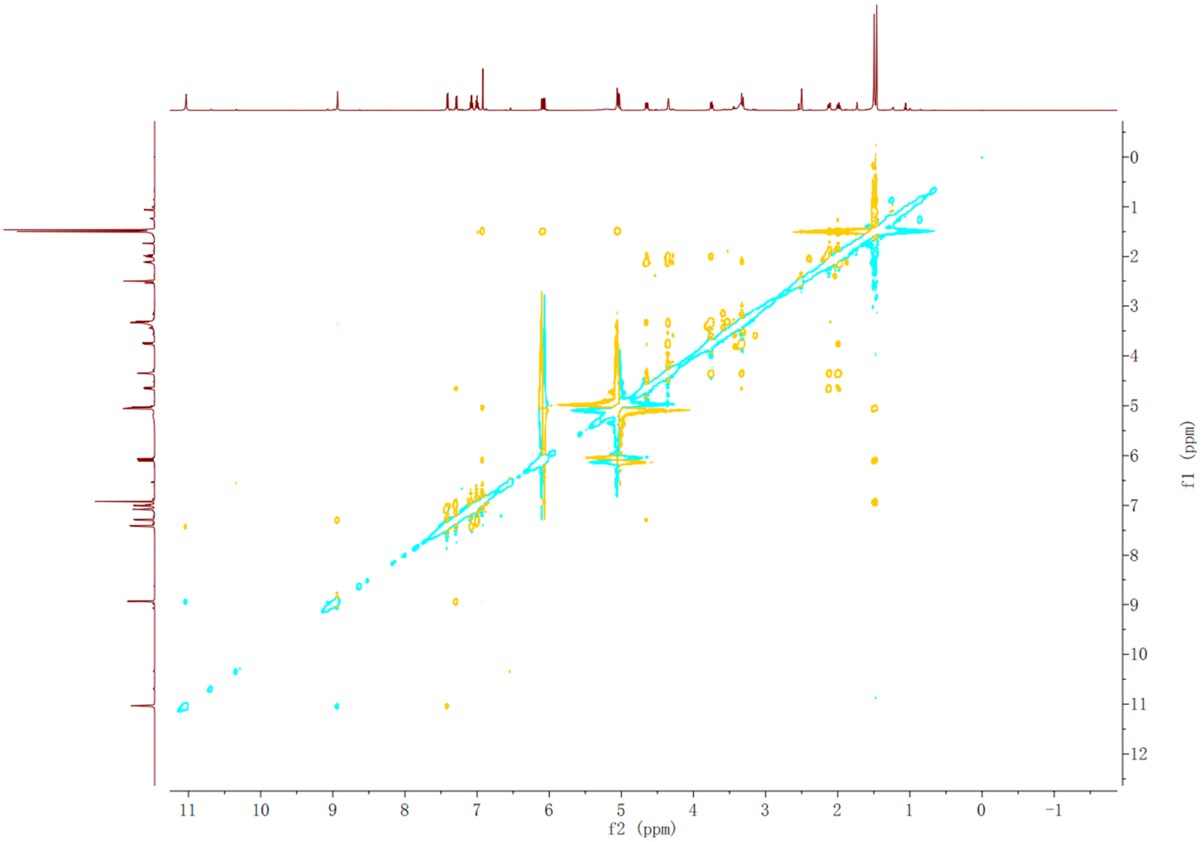


**Figure S20.** ROESY spectra (DMSO-$d_6$) of **3**.

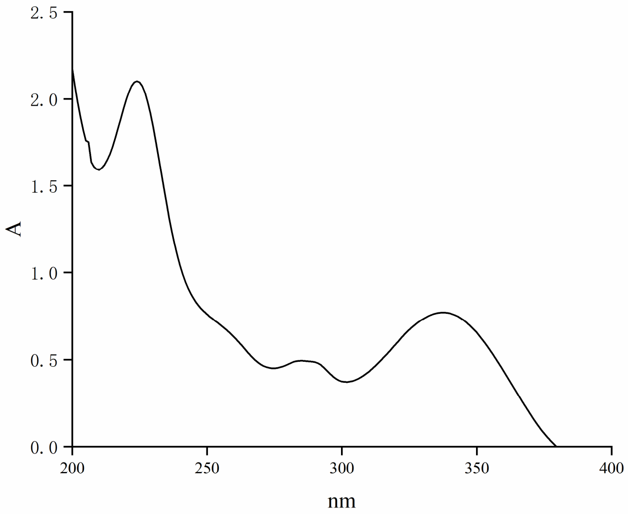


**Figure S21.** UV spectra (MeOH) of **3**.

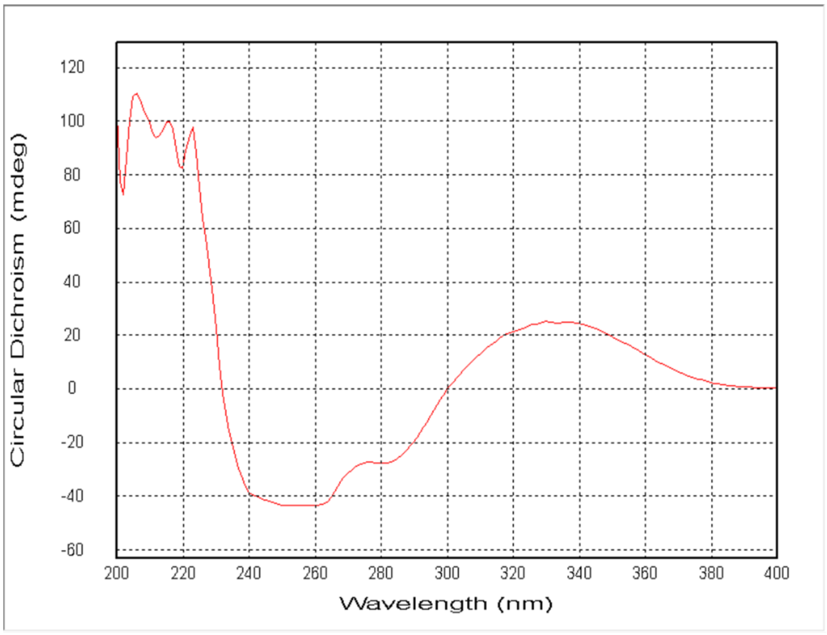


**Figure S22.** ECD spectra (MeOH) of **3**.

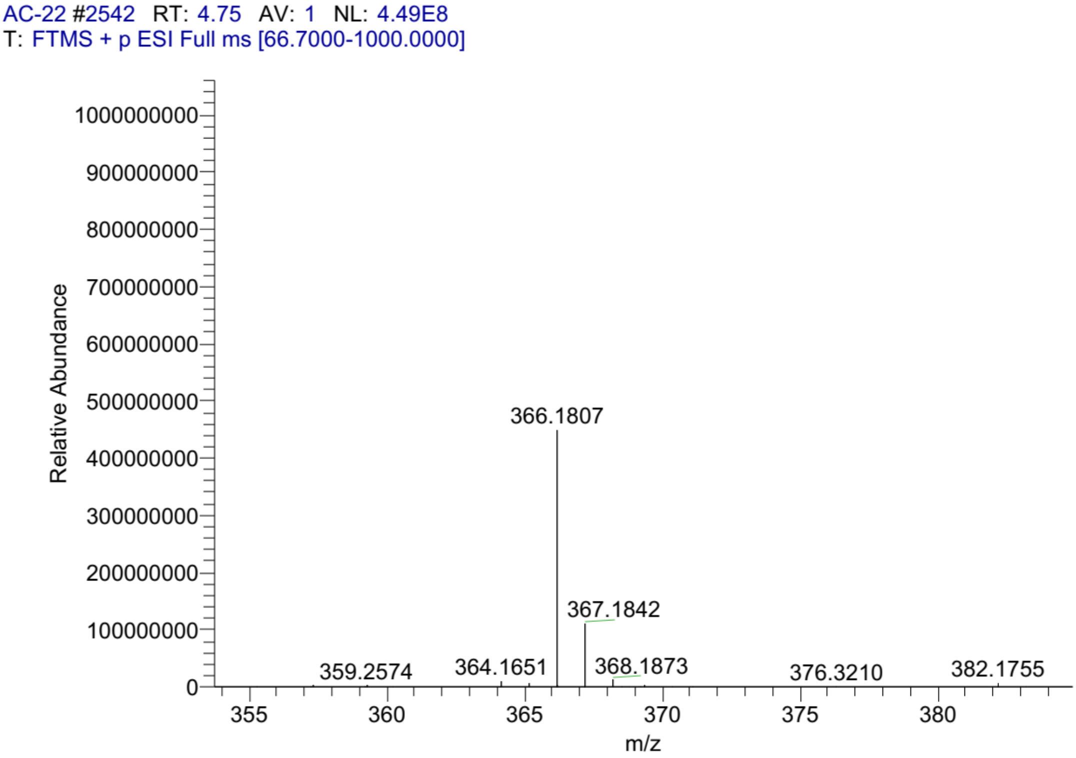


**Figure S23.** HRESIMS spectra of **3**.

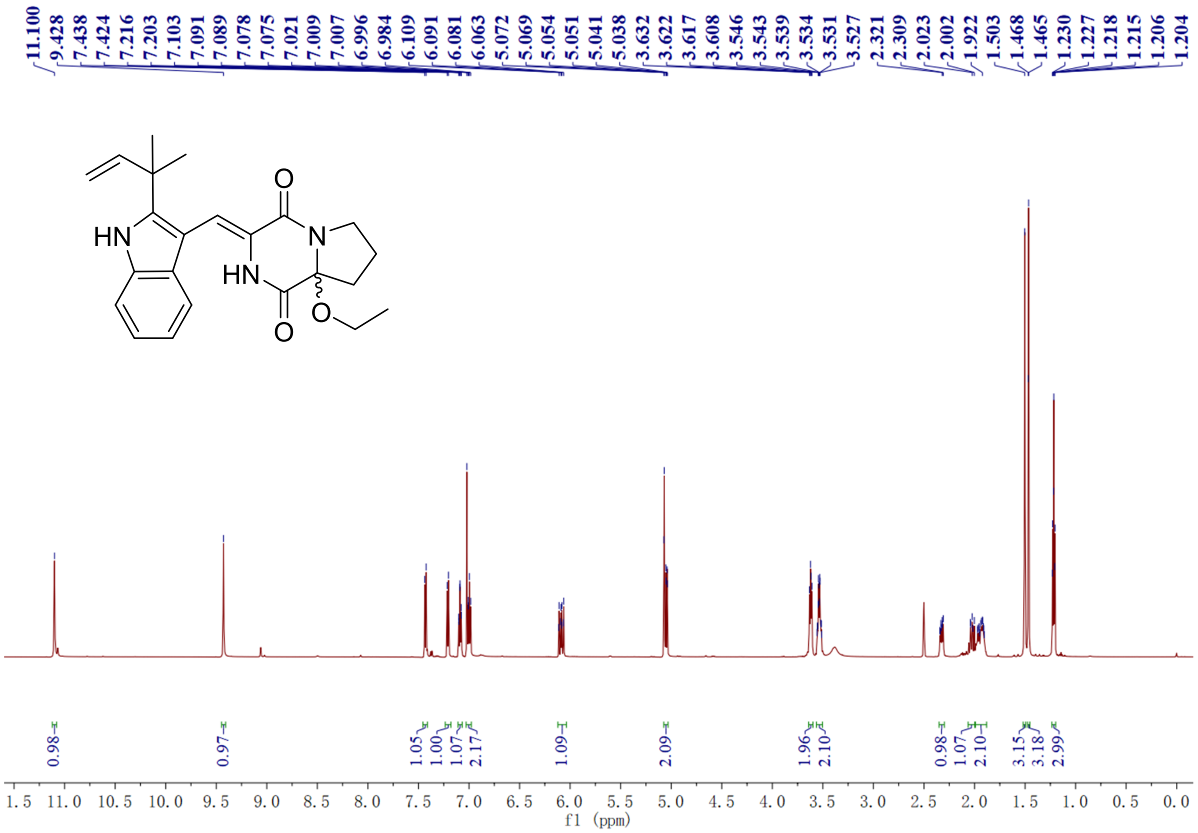


**Figure S24.** $^{1}$H NMR spectra (600 MHz, DMSO-$d_6$) of **4**.

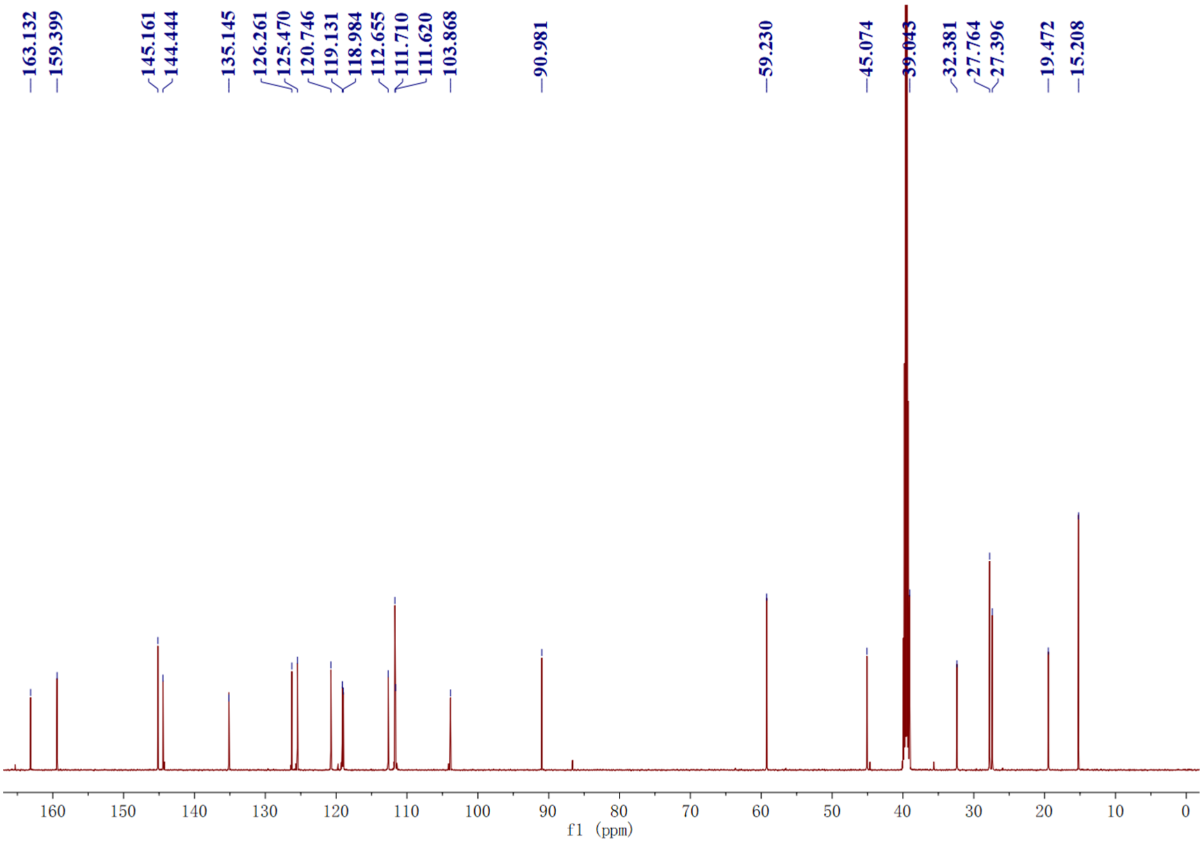


**Figure S25.** $^{13}$C NMR spectra (150 MHz, DMSO-$d_6$) of **4**.

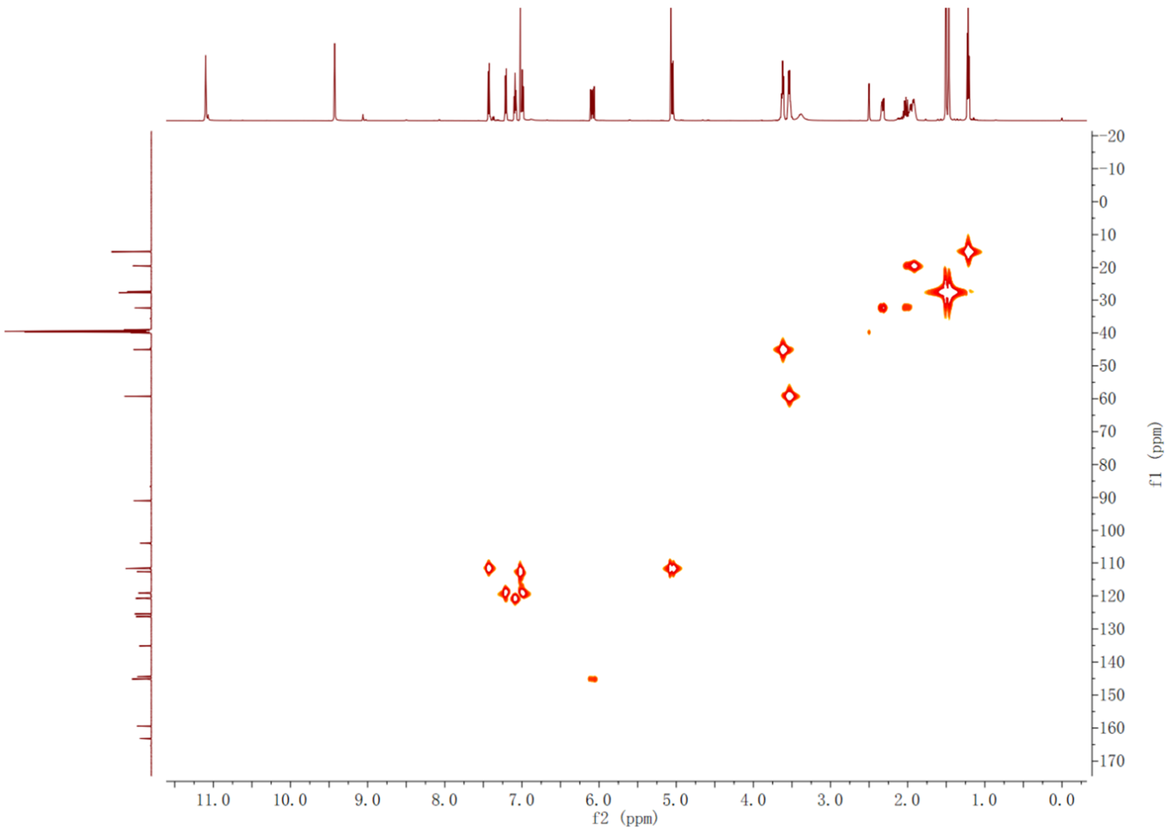


**Figure S26.** HMQC spectra (DMSO-$d_6$) of **4**.

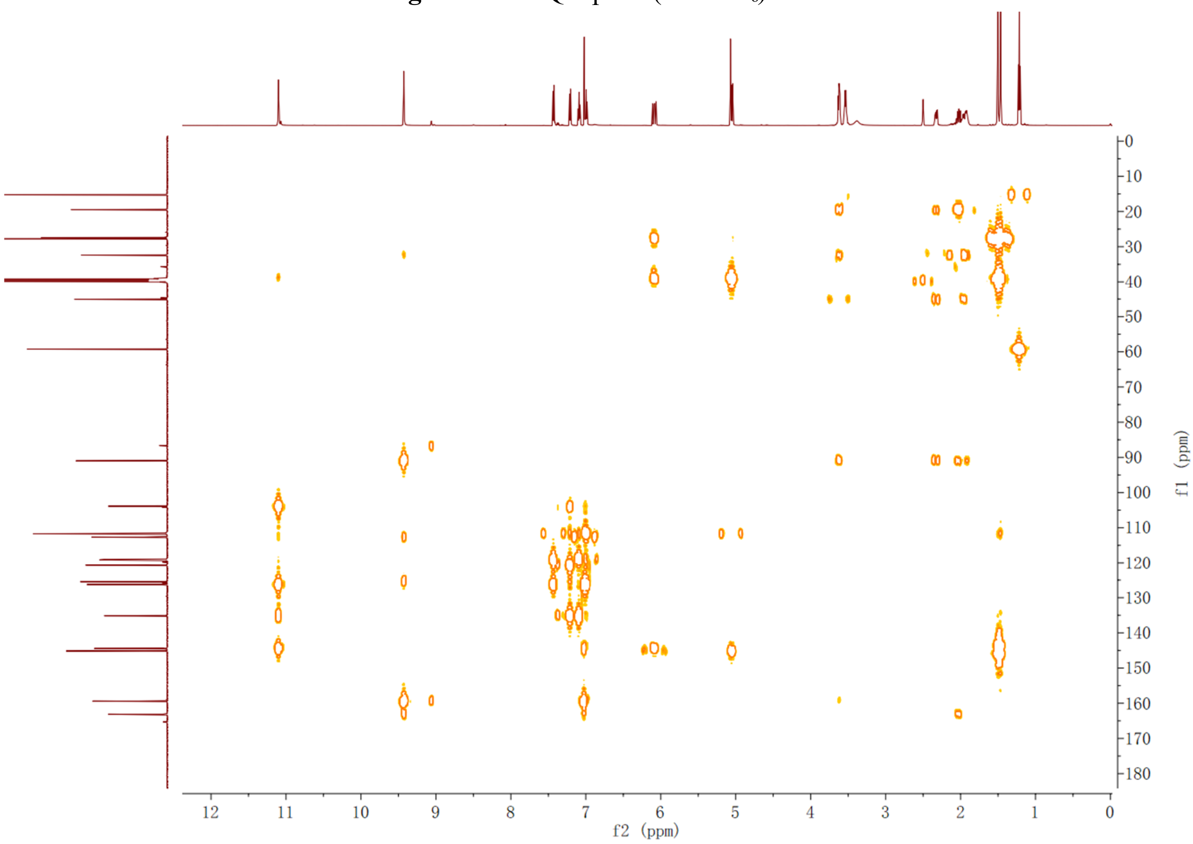


**Figure S27.** HMBC spectra (DMSO-$d_6$) of **4**.

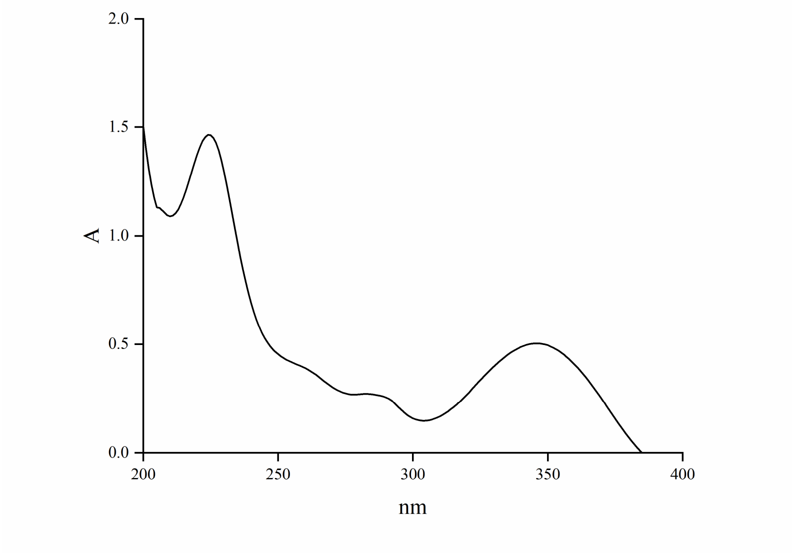


**Figure S28.** UV spectra (MeOH) of **4**.

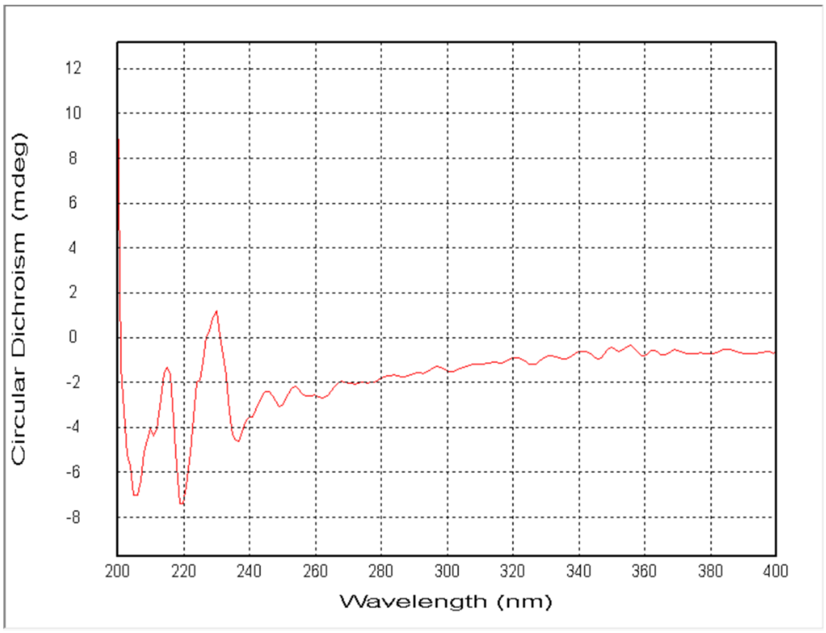


**Figure S29.** ECD spectra (MeOH) of **4**.

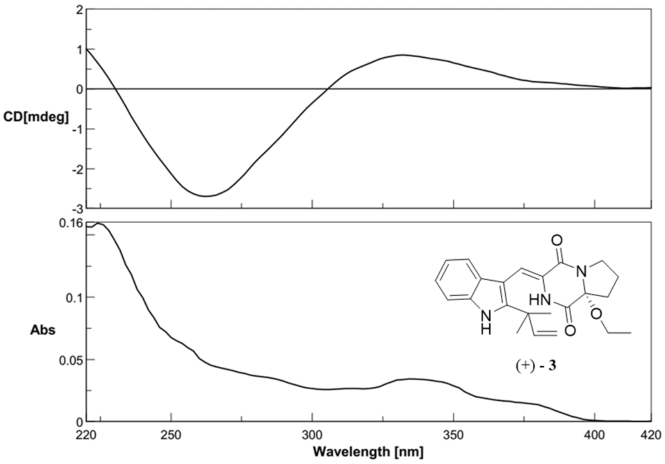


**Figure S30.** ECD spectra (MeOH) of (+) - **4**.

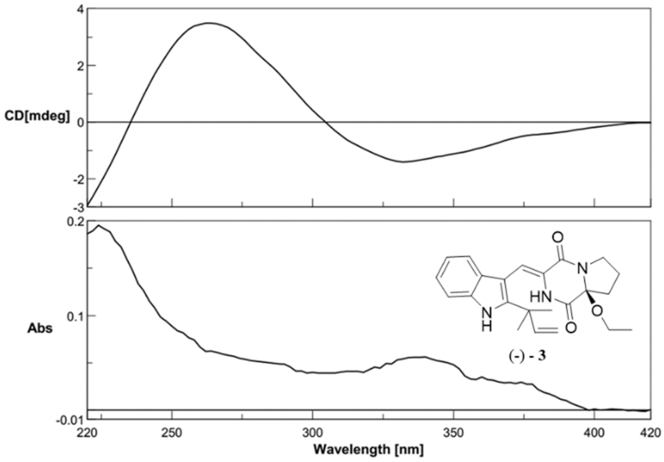


**Figure S31.** ECD spectra (MeOH) of (-) - **4**.

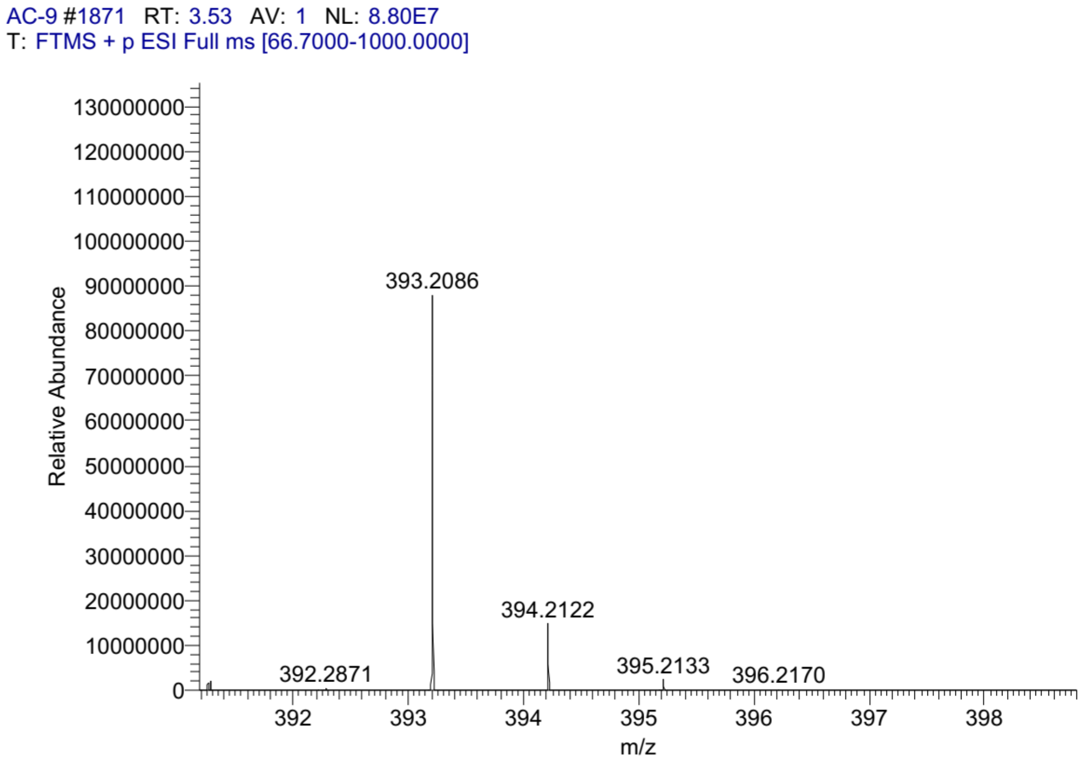


**Figure S32.** HRESIMS spectra of **4**.

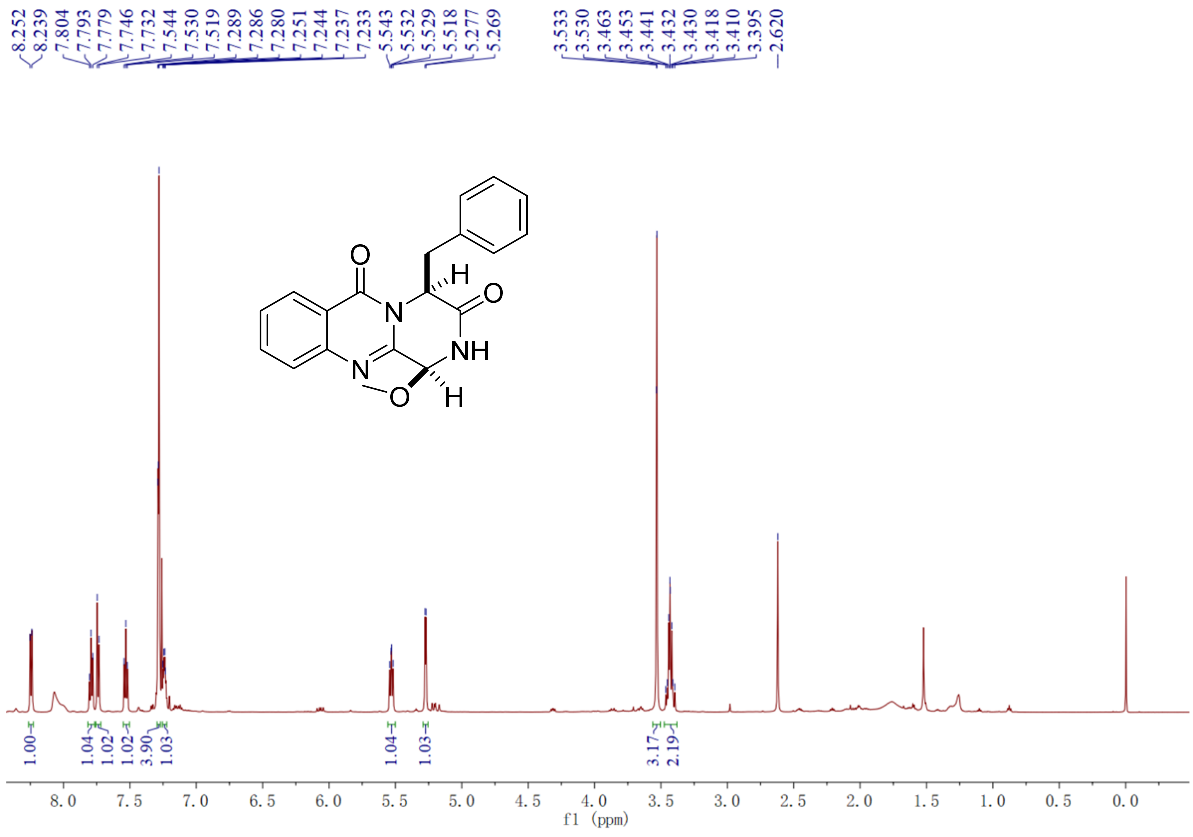


**Figure S33.** $^{1}$H NMR spectra (600 MHz, $CDCl_3$) of **5**.

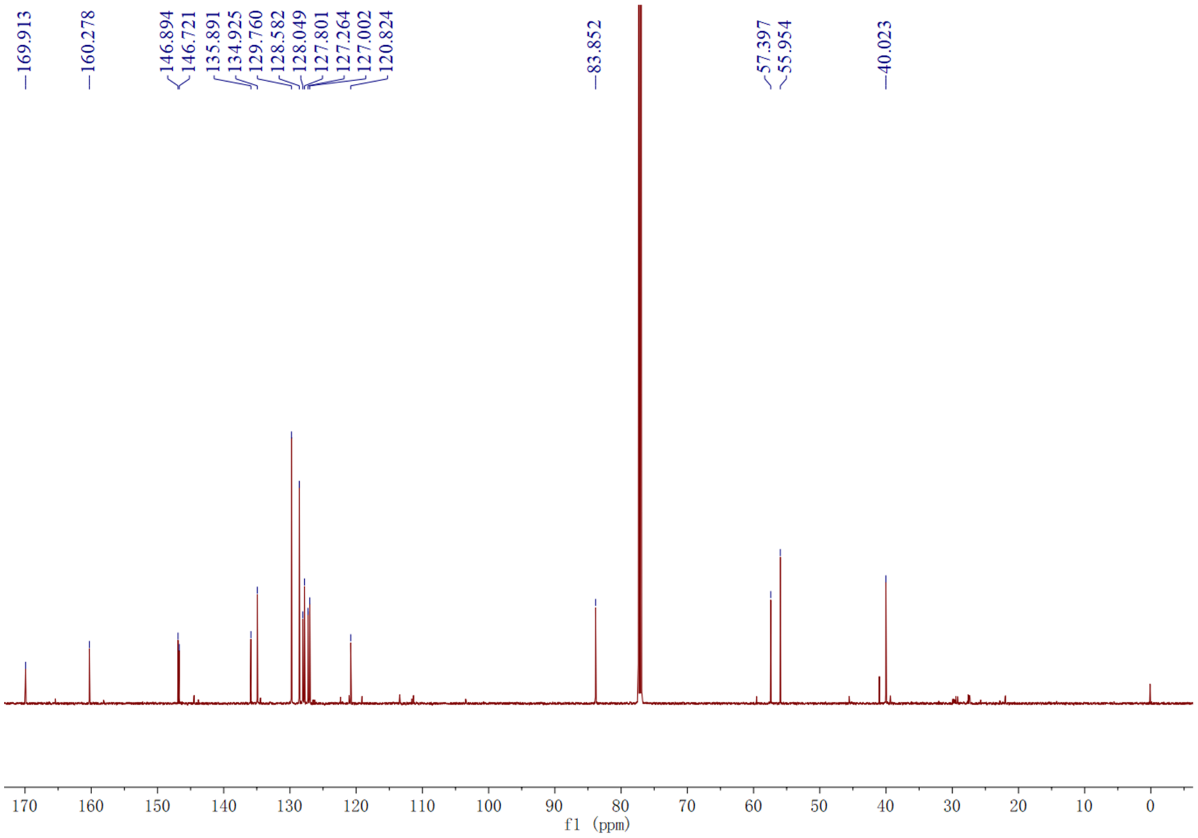


**Figure S34.** $^{13}C$ NMR spectra (150 MHz, $CDCl_3$) of **5**.

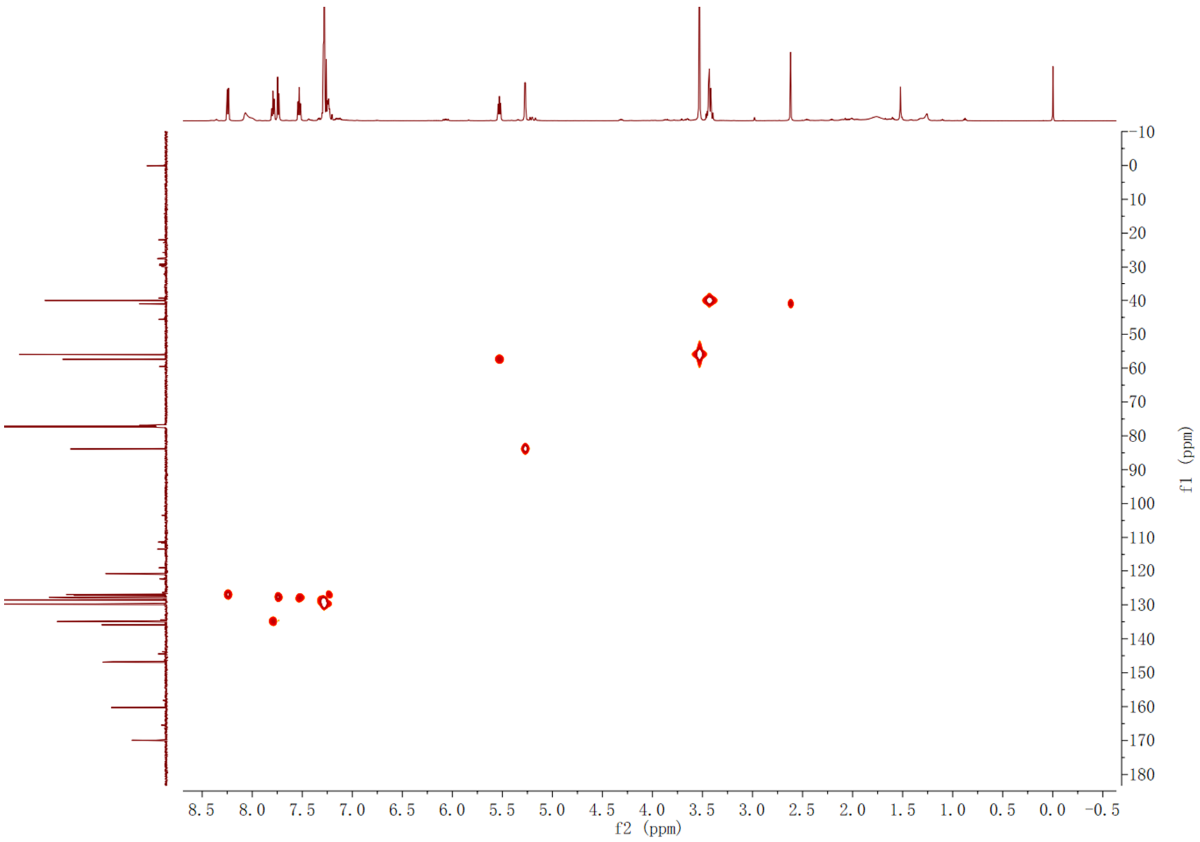


**Figure S35.** HMQC spectra ($CDCl_3$) of **5**.

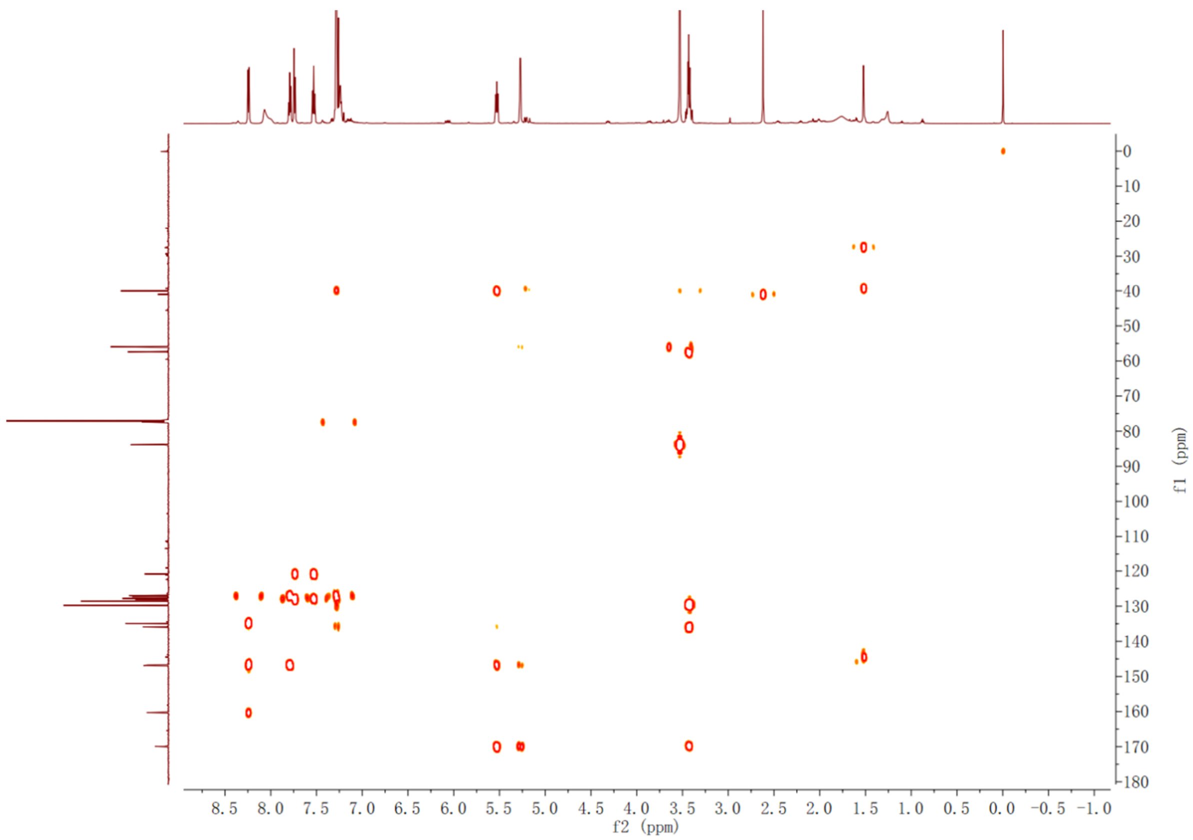


**Figure S36.** HMBC spectra ($CDCl_3$) of **5**.

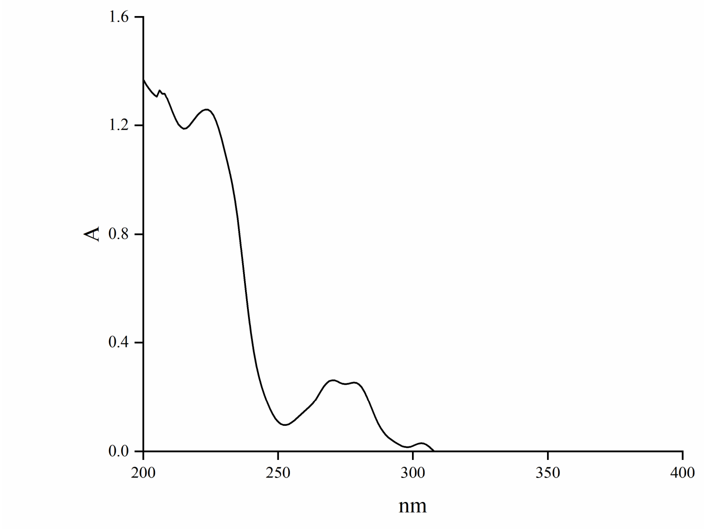


**Figure S37.** UV spectra (MeOH) of **5**.

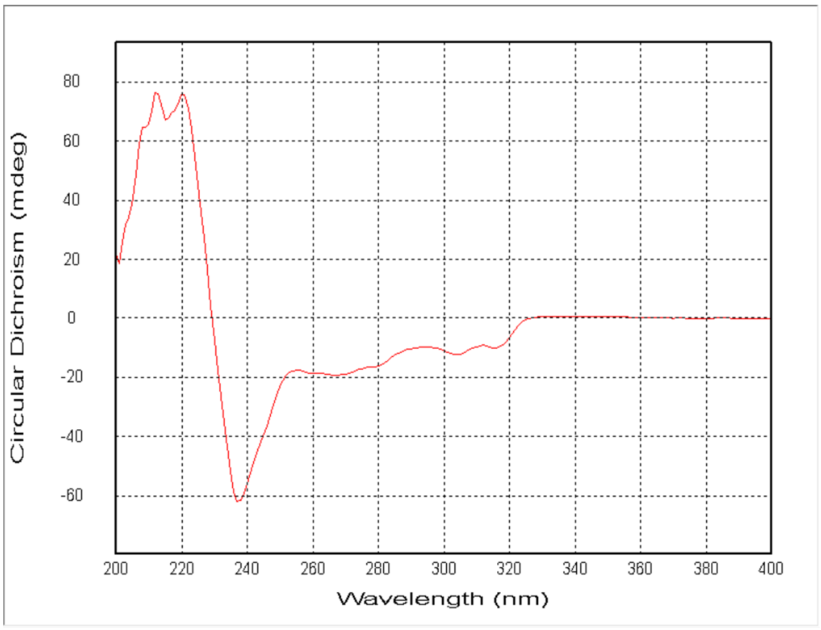


**Figure S38.** ECD spectra (MeOH) of **5**.

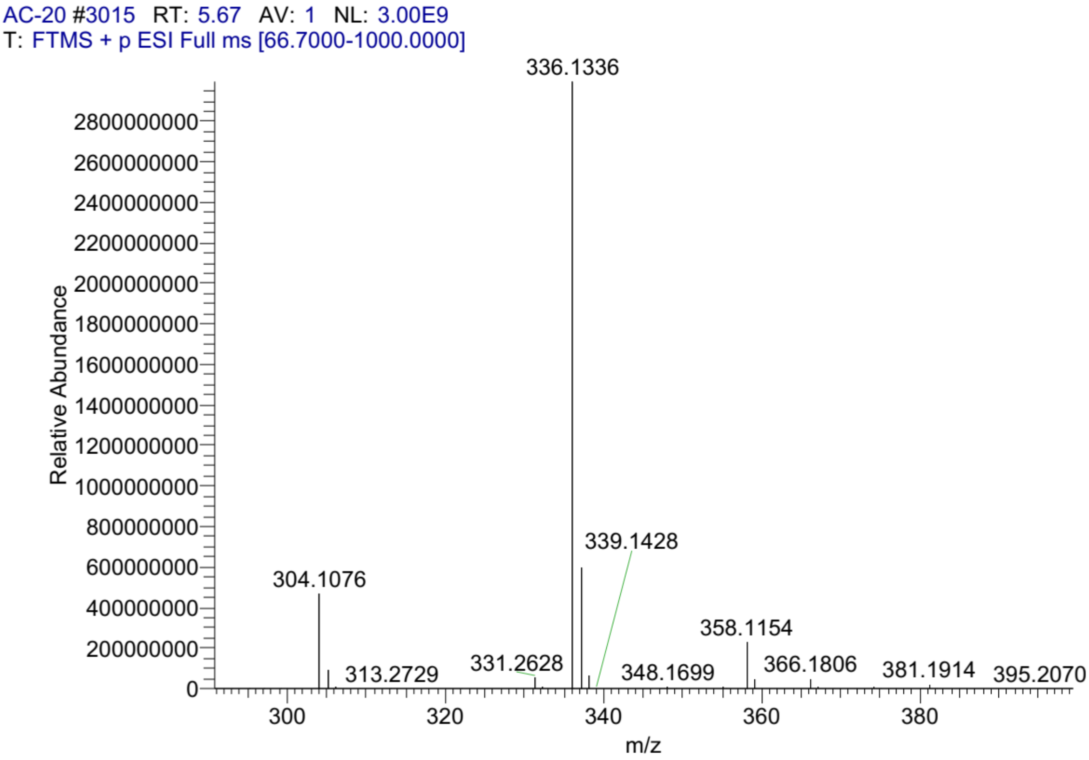


**Figure S39.** HRESIMS spectra of **5**.

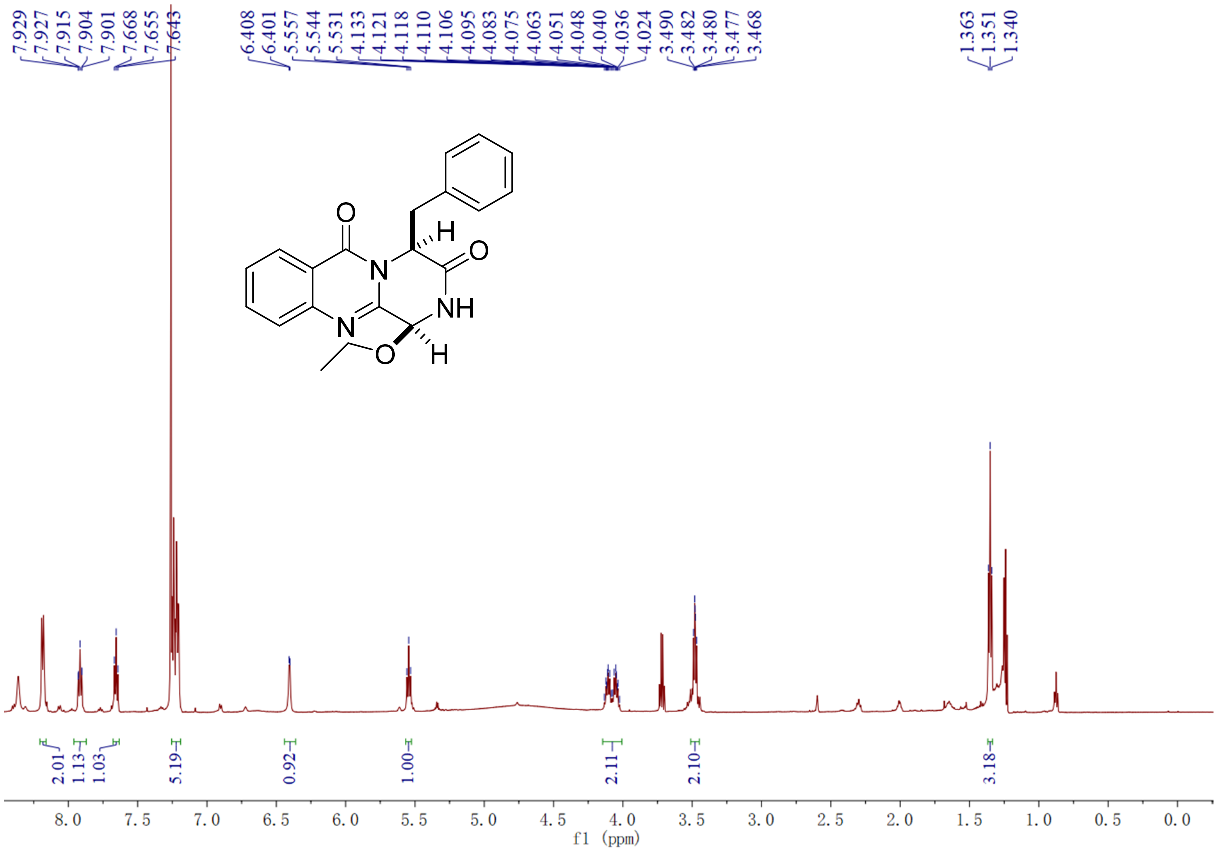


**Figure S40.** $^{1}$H NMR spectra (600 MHz, $CDCl_3$) of **6**.

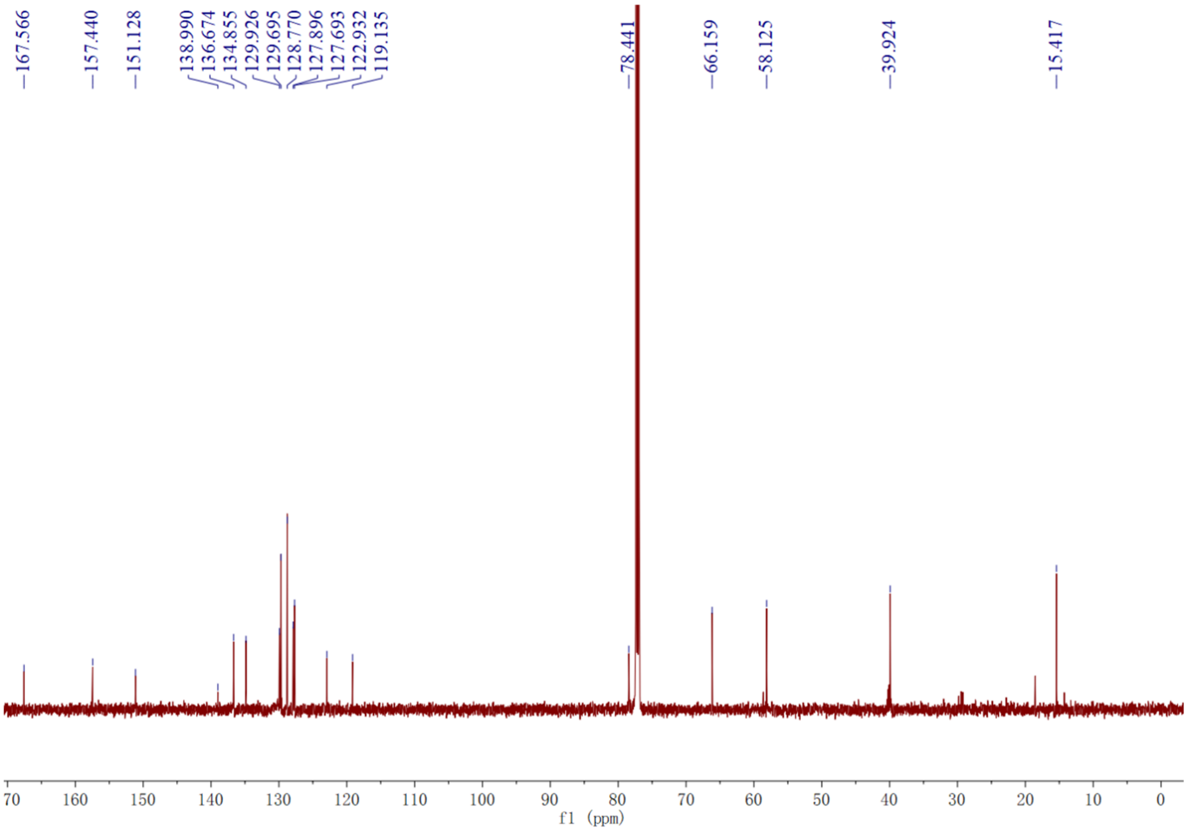


**Figure S41.** $^{13}$C NMR spectra (150 MHz, $CDCl_3$) of **6**.

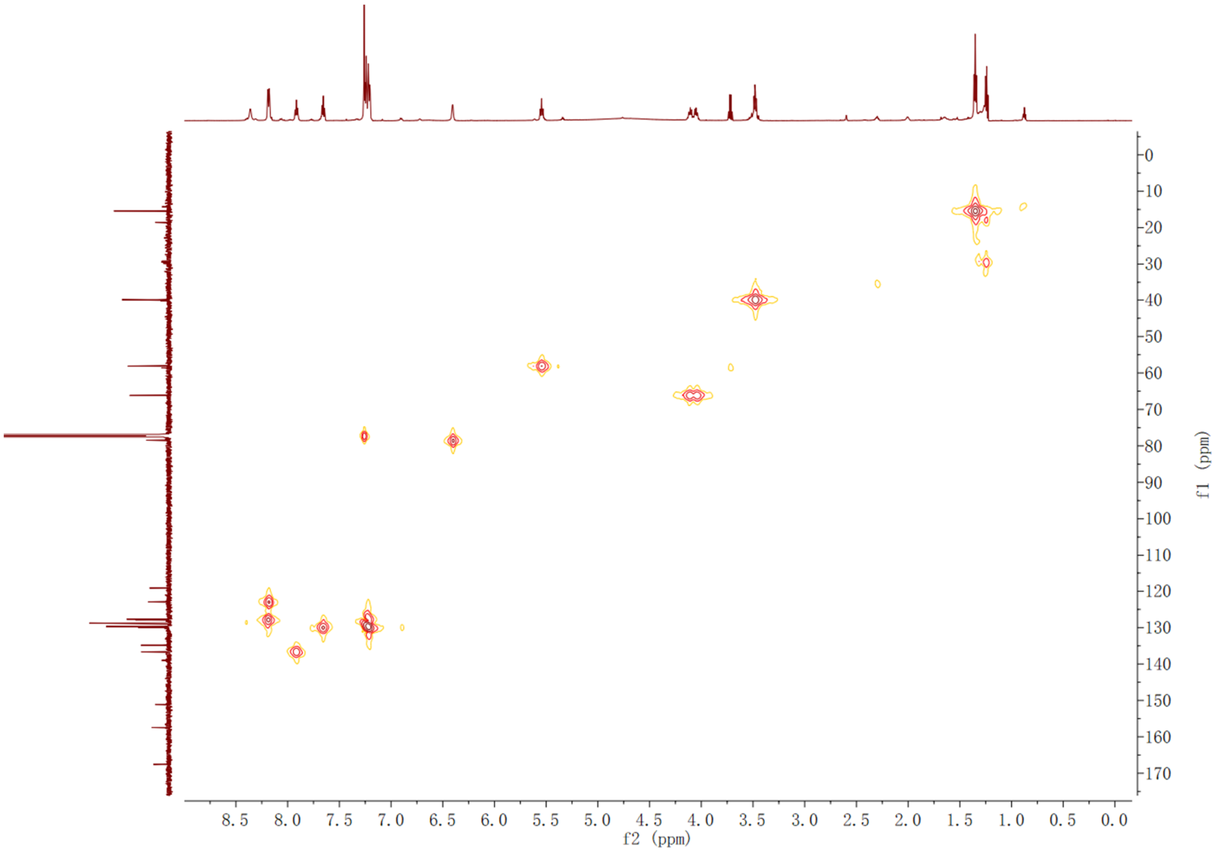


**Figure S42.** HMQC spectra ($CDCl_3$) of **6**.

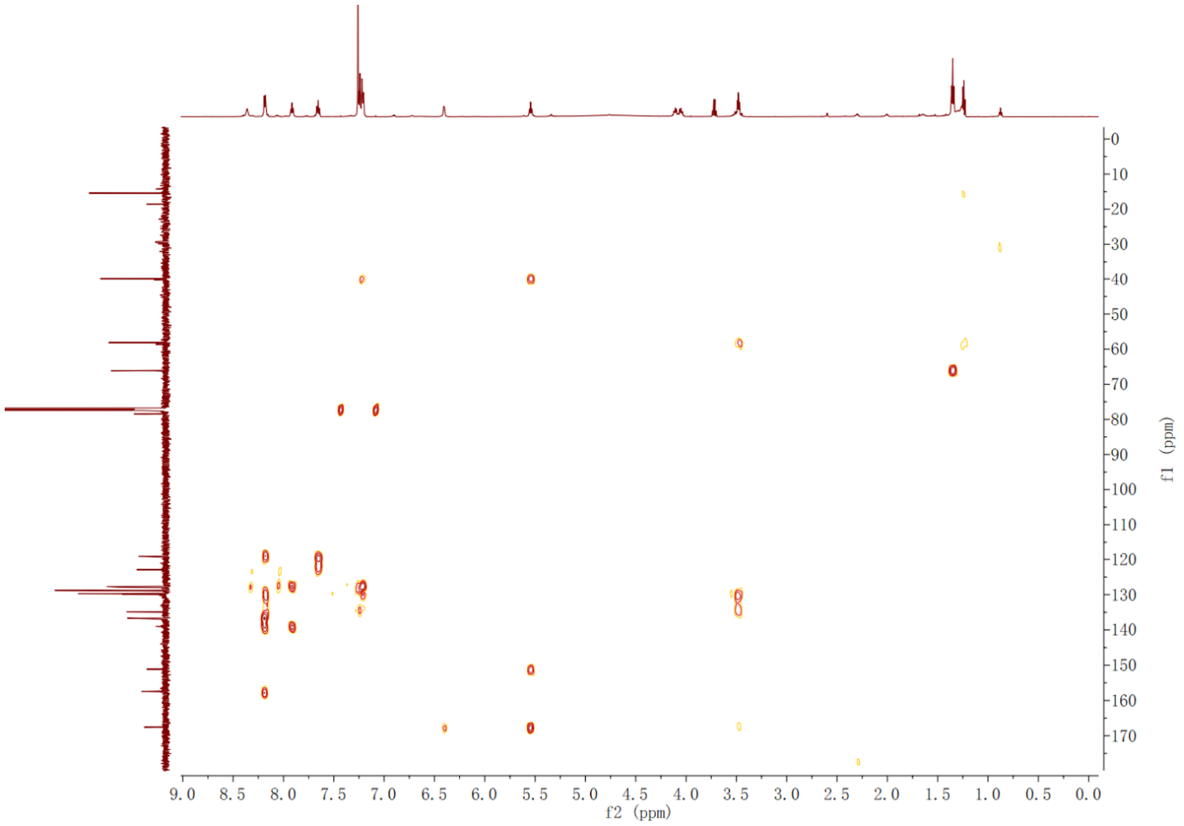


**Figure S43.** HMBC spectra ($CDCl_3$) of **6**.

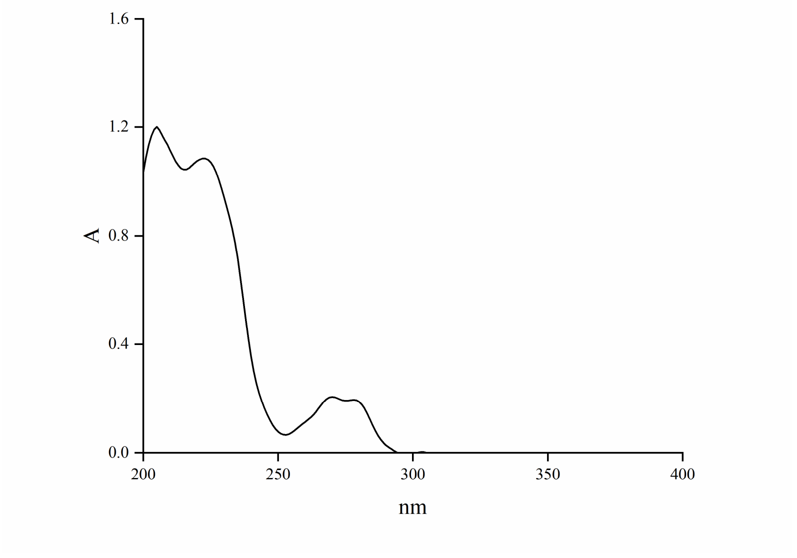


**Figure S44.** UV spectra (MeOH) of **6**.

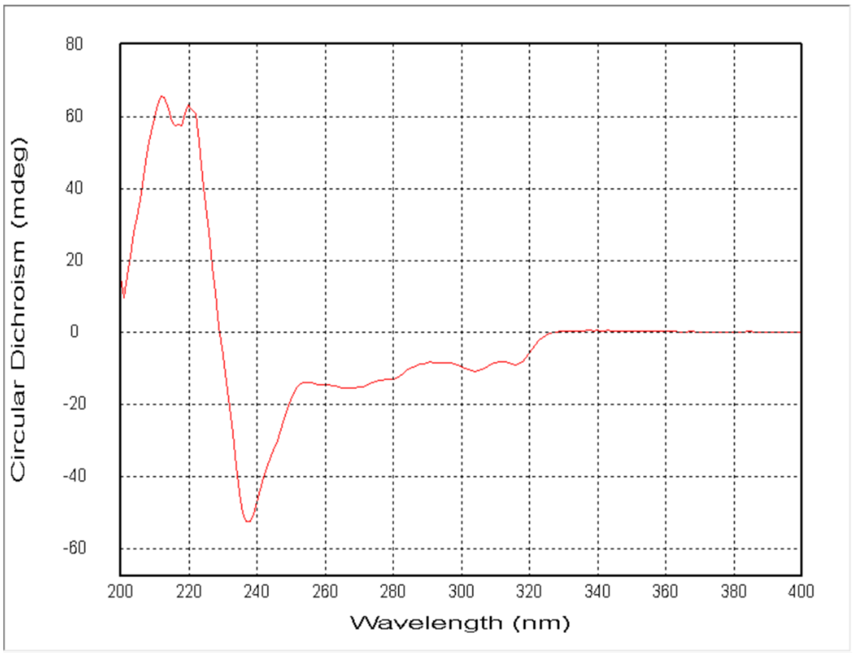


**Figure S45.** ECD spectra (MeOH) of **6**.

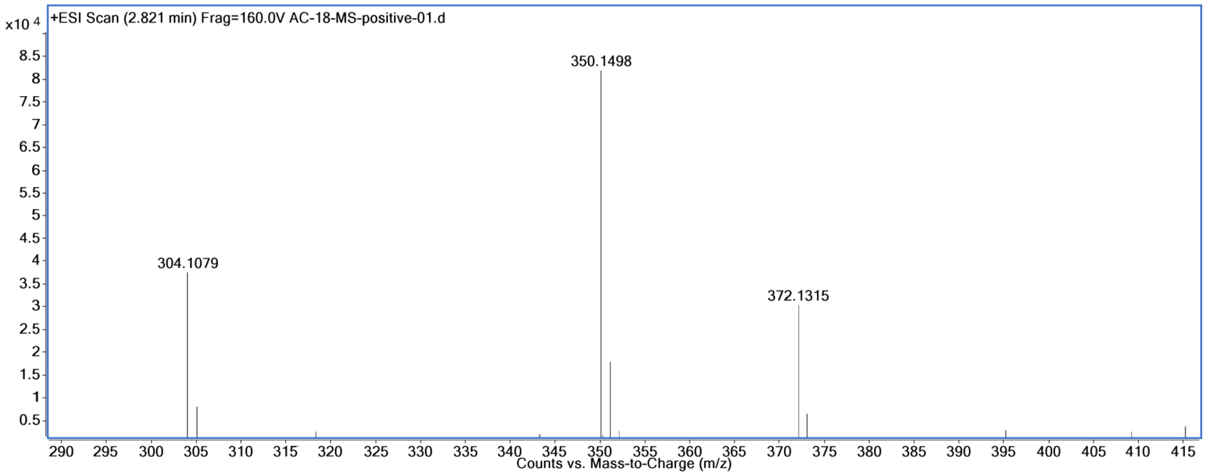


**Figure S46.** HRESIMS spectra of **6**.

**Table S1.** Energies of conformers of **1** with Boltzmann distribution over 1% at MMFF.

| Label | E (kcal/mol) | rel. E (kcal/mol) | Boltzmann Dist |
|---|---|---|---|
| M0001 | 51.83 | 0.00 | 0.214 |
| M0002 | 52.03 | 0.20 | 0.153 |
| M0003 | 52.17 | 0.34 | 0.121 |
| M0004 | 52.26 | 0.43 | 0.103 |
| M0005 | 52.26 | 0.43 | 0.103 |
| M0006 | 52.55 | 0.72 | 0.064 |
| M0007 | 52.66 | 0.83 | 0.053 |
| M0008 | 52.84 | 1.01 | 0.039 |
| M0009 | 53.26 | 1.43 | 0.019 |
| M0010 | 53.45 | 1.62 | 0.014 |
| M0011 | 53.53 | 1.70 | 0.012 |
| M0012 | 53.62 | 1.79 | 0.010 |

**Table S2.** Gibbs free energies[a] and equilibrium populations[b] of low-energy conformers of **1**.

| Conformers | In MeOH | |
|---|---|---|
| | *G* | *P* (%) |
| **1-1** | -804640.53898995 | 21.78 |
| **1-2** | -804640.19950704 | 12.27 |
| **1-3** | -804640.14616869 | 11.22 |
| **1-4** | -804639.38311653 | 3.09 |
| **1-5** | -804639.77217273 | 5.96 |
| **1-6** | -804638.47824711 | 0.67 |
| **1-7** | -804640.38022992 | 16.66 |
| **1-8** | -804639.18419586 | 2.21 |
| **1-9** | -804640.14616869 | 11.22 |
| **1-10** | -804639.64165065 | 4.78 |

| 1-11 | -804639.93281529 | 7.82 |
|---|---|---|
| **1-12** | -804639.21243381 | 2.32 |

[a]B3LYP/6-31G(d,p), in kcal/mol. [b]From *G* values at 298.15K.

**Table S3.** Cartesian coordinates for the low-energy reoptimized MMFF conformers of **1** at B3LYP/6-31+G(d,p) level of theory in gas.

| **1-1** | | Standard Orientation (Ångstroms) | | | |
|---|---|---|---|---|---|
| Center number | Atomic number | Atomic Type | X | Y | Z |
| 1. | 6. | 0. | 4.644157 | 2.411265 | 0.291423 |
| 2. | 6. | 0. | 3.805298 | 1.304376 | 0.136323 |
| 3. | 6. | 0. | 2.437460 | 1.419027 | -0.224611 |
| 4. | 6. | 0. | 1.920899 | 2.703780 | -0.478794 |
| 5. | 6. | 0. | 2.749067 | 3.809151 | -0.325865 |
| 6. | 6. | 0. | 4.095674 | 3.667392 | 0.062220 |
| 7. | 7. | 0. | 4.080717 | -0.040490 | 0.258156 |
| 8. | 6. | 0. | 2.958320 | -0.793353 | -0.009286 |
| 9. | 6. | 0. | 1.903761 | 0.069691 | -0.309795 |
| 10. | 6. | 0. | 3.050259 | -2.319121 | 0.032501 |
| 11. | 7. | 0. | -0.748569 | 1.274035 | 0.574396 |
| 12. | 6. | 0. | -0.619346 | 0.226627 | -0.359857 |
| 13. | 6. | 0. | 0.562170 | -0.334789 | -0.702772 |
| 14. | 6. | 0. | -1.885417 | 2.008200 | 0.809546 |
| 15. | 6. | 0. | -2.986086 | 1.720500 | -0.199096 |
| 16. | 7. | 0. | -3.019211 | 0.296191 | -0.565213 |
| 17. | 6. | 0. | -1.885587 | -0.406431 | -0.864255 |
| 18. | 6. | 0. | -4.424297 | 2.005275 | 0.229864 |
| 19. | 6. | 0. | -5.216045 | 1.095870 | -0.727859 |
| 20. | 6. | 0. | -4.392165 | -0.200003 | -0.767762 |
| 21. | 8. | 0. | -1.894723 | -1.493102 | -1.441591 |
| 22. | 8. | 0. | -1.972394 | 2.845059 | 1.693655 |
| 23. | 6. | 0. | 4.266252 | -2.714397 | 0.863315 |
| 24. | 6. | 0. | 3.136749 | -2.857227 | -1.413952 |
| 25. | 6. | 0. | 5.389763 | -3.292762 | 0.435938 |
| 26. | 6. | 0. | 1.819028 | -2.937233 | 0.745144 |
| 27. | 8. | 0. | -4.848424 | -1.014020 | 0.288025 |
| 28. | 6. | 0. | -4.373742 | -2.363634 | 0.307446 |
| 29. | 6. | 0. | -5.212359 | -3.130251 | 1.315772 |
| 30. | 1. | 0. | -2.747981 | 2.324147 | -1.093011 |
| 31. | 1. | 0. | 5.686855 | 2.293109 | 0.571907 |
| 32. | 1. | 0. | 0.892835 | 2.832783 | -0.798148 |

| | | | | | |
|---|---|---|---|---|---|
| 33. | 1. | 0. | 2.352367 | 4.802307 | -0.513443 |
| 34. | 1. | 0. | 4.716626 | 4.550870 | 0.174571 |
| 35. | 1. | 0. | 4.971867 | -0.447905 | 0.498075 |
| 36. | 1. | 0. | 0.048147 | 1.492708 | 1.161298 |
| 37. | 1. | 0. | 0.449360 | -1.214020 | -1.328953 |
| 38. | 1. | 0. | -4.570877 | 1.696487 | 1.268152 |
| 39. | 1. | 0. | -4.677110 | 3.063364 | 0.145518 |
| 40. | 1. | 0. | -6.235420 | 0.888917 | -0.399528 |
| 41. | 1. | 0. | -5.254599 | 1.538714 | -1.729672 |
| 42. | 1. | 0. | -4.438919 | -0.747243 | -1.717160 |
| 43. | 1. | 0. | 4.168073 | -2.493994 | 1.927330 |
| 44. | 1. | 0. | 3.222737 | -3.948520 | -1.414092 |
| 45. | 1. | 0. | 2.239245 | -2.588618 | -1.976476 |
| 46. | 1. | 0. | 3.997212 | -2.440610 | -1.945595 |
| 47. | 1. | 0. | 6.183572 | -3.551300 | 1.130550 |
| 48. | 1. | 0. | 5.558428 | -3.547284 | -0.605667 |
| 49. | 1. | 0. | 1.719215 | -2.546884 | 1.762639 |
| 50. | 1. | 0. | 0.889935 | -2.727578 | 0.213301 |
| 51. | 1. | 0. | 1.940453 | -4.023286 | 0.806215 |
| 52. | 1. | 0. | -3.311866 | -2.393072 | 0.578456 |
| 53. | 1. | 0. | -4.460313 | -2.802846 | -0.696680 |
| 54. | 1. | 0. | -6.268335 | -3.120302 | 1.030116 |
| 55. | 1. | 0. | -4.877152 | -4.170592 | 1.374525 |
| 56. | 1. | 0. | -5.123479 | -2.682730 | 2.310141 |

| **1-2** | | Standard Orientation (Ångstroms) | | | |
|---|---|---|---|---|---|
| Center number | Atom number | Type | X | Y | Z |
| 1. | 6. | 0. | 4.166535 | 2.896443 | 0.282706 |
| 2. | 6. | 0. | 3.544364 | 1.645954 | 0.237308 |
| 3. | 6. | 0. | 2.146764 | 1.477385 | 0.422249 |
| 4. | 6. | 0. | 1.367873 | 2.614016 | 0.713528 |
| 5. | 6. | 0. | 1.981039 | 3.860746 | 0.758128 |
| 6. | 6. | 0. | 3.364669 | 4.003263 | 0.536758 |
| 7. | 7. | 0. | 4.079350 | 0.390975 | 0.049545 |
| 8. | 6. | 0. | 3.091366 | -0.571510 | 0.100135 |
| 9. | 6. | 0. | 1.868599 | 0.056579 | 0.320882 |
| 10. | 6. | 0. | 3.476396 | -2.043886 | -0.036671 |
| 11. | 7. | 0. | -0.733788 | 0.830336 | -1.007589 |
| 12. | 6. | 0. | -0.600519 | -0.231884 | -0.090630 |
| 13. | 6. | 0. | 0.567842 | -0.587540 | 0.488500 |

| | | | | | |
|---|---|---|---|---|---|
| 14. | 6. | 0. | -1.813357 | 1.060143 | -1.825792 |
| 15. | 6. | 0. | -2.838237 | -0.060322 | -1.786731 |
| 16. | 7. | 0. | -2.950026 | -0.650799 | -0.445182 |
| 17. | 6. | 0. | -1.853081 | -0.963701 | 0.308558 |
| 18. | 6. | 0. | -4.283915 | 0.285660 | -2.138614 |
| 19. | 6. | 0. | -5.043558 | -0.879017 | -1.476329 |
| 20. | 6. | 0. | -4.329027 | -1.065663 | -0.129340 |
| 21. | 8. | 0. | -1.886938 | -1.733717 | 1.266507 |
| 22. | 8. | 0. | -1.895992 | 2.027086 | -2.565984 |
| 23. | 6. | 0. | 4.801146 | -2.135056 | -0.785931 |
| 24. | 6. | 0. | 2.438443 | -2.818616 | -0.887100 |
| 25. | 6. | 0. | 5.979678 | -2.535615 | -0.306057 |
| 26. | 6. | 0. | 3.559168 | -2.670820 | 1.373556 |
| 27. | 8. | 0. | -4.962896 | -0.214536 | 0.796978 |
| 28. | 6. | 0. | -4.586330 | -0.367981 | 2.169017 |
| 29. | 6. | 0. | -5.598503 | 0.390515 | 3.010294 |
| 30. | 1. | 0. | -2.470914 | -0.824830 | -2.494806 |
| 31. | 1. | 0. | 5.237750 | 2.998778 | 0.134695 |
| 32. | 1. | 0. | 0.307007 | 2.520737 | 0.924031 |
| 33. | 1. | 0. | 1.384338 | 4.741087 | 0.976550 |
| 34. | 1. | 0. | 3.814156 | 4.990638 | 0.577732 |
| 35. | 1. | 0. | 5.050649 | 0.173489 | -0.114259 |
| 36. | 1. | 0. | 0.028144 | 1.493083 | -1.094241 |
| 37. | 1. | 0. | 0.479598 | -1.429702 | 1.169066 |
| 38. | 1. | 0. | -4.562052 | 1.239436 | -1.682431 |
| 39. | 1. | 0. | -4.438601 | 0.359509 | -3.216111 |
| 40. | 1. | 0. | -6.105017 | -0.682826 | -1.319693 |
| 41. | 1. | 0. | -4.941470 | -1.790121 | -2.076844 |
| 42. | 1. | 0. | -4.316869 | -2.095726 | 0.247464 |
| 43. | 1. | 0. | 4.739881 | -1.838972 | -1.834177 |
| 44. | 1. | 0. | 2.765264 | -3.856155 | -1.007500 |
| 45. | 1. | 0. | 1.453295 | -2.821876 | -0.418301 |
| 46. | 1. | 0. | 2.335351 | -2.373572 | -1.881429 |
| 47. | 1. | 0. | 6.856627 | -2.580538 | -0.945115 |
| 48. | 1. | 0. | 6.116872 | -2.849407 | 0.723967 |
| 49. | 1. | 0. | 4.283268 | -2.146454 | 2.003778 |
| 50. | 1. | 0. | 2.587170 | -2.615231 | 1.870408 |
| 51. | 1. | 0. | 3.850770 | -3.724179 | 1.311545 |
| 52. | 1. | 0. | -3.573612 | 0.018909 | 2.333673 |
| 53. | 1. | 0. | -4.568812 | -1.434808 | 2.433022 |
| 54. | 1. | 0. | -6.604464 | -0.014659 | 2.866600 |
| 55. | 1. | 0. | -5.340922 | 0.315869 | 4.071467 |

| | | | | | |
|---|---|---|---|---|---|
| 56. | 1. | 0. | -5.613683 | 1.448711 | 2.732942 |

| **1-3** | | Standard Orientation (Ångstroms) | | | |
|---|---|---|---|---|---|
| Center number | Atom number | Type | X | Y | Z |
| 1. | 6. | 0. | -4.588818 | -2.419101 | 0.278286 |
| 2. | 6. | 0. | -3.768987 | -1.303181 | 0.086659 |
| 3. | 6. | 0. | -2.415410 | -1.406676 | -0.324229 |
| 4. | 6. | 0. | -1.888519 | -2.686593 | -0.579254 |
| 5. | 6. | 0. | -2.695830 | -3.800706 | -0.385589 |
| 6. | 6. | 0. | -4.031127 | -3.670313 | 0.044503 |
| 7. | 7. | 0. | -4.052126 | 0.039142 | 0.205233 |
| 8. | 6. | 0. | -2.951820 | 0.803495 | -0.129599 |
| 9. | 6. | 0. | -1.898140 | -0.053625 | -0.448332 |
| 10. | 6. | 0. | -3.060284 | 2.317261 | 0.053638 |
| 11. | 7. | 0. | 0.707536 | -1.210254 | 0.579590 |
| 12. | 6. | 0. | 0.618026 | -0.223394 | -0.422554 |
| 13. | 6. | 0. | -0.545676 | 0.325596 | -0.838533 |
| 14. | 6. | 0. | 1.827188 | -1.940496 | 0.895504 |
| 15. | 6. | 0. | 2.963676 | -1.724911 | -0.090787 |
| 16. | 7. | 0. | 3.023030 | -0.327062 | -0.544984 |
| 17. | 6. | 0. | 1.907032 | 0.362845 | -0.928576 |
| 18. | 6. | 0. | 4.384550 | -1.994293 | 0.400977 |
| 19. | 6. | 0. | 5.214991 | -1.155055 | -0.587708 |
| 20. | 6. | 0. | 4.406745 | 0.142636 | -0.735727 |
| 21. | 8. | 0. | 1.946279 | 1.407166 | -1.577550 |
| 22. | 8. | 0. | 1.874563 | -2.725230 | 1.828998 |
| 23. | 6. | 0. | -4.477264 | 2.732223 | -0.329011 |
| 24. | 6. | 0. | -2.102817 | 3.104594 | -0.871568 |
| 25. | 6. | 0. | -5.420341 | 3.232723 | 0.471140 |
| 26. | 6. | 0. | -2.726785 | 2.656335 | 1.525316 |
| 27. | 8. | 0. | 4.839599 | 1.017522 | 0.280639 |
| 28. | 6. | 0. | 4.371593 | 2.367433 | 0.206191 |
| 29. | 6. | 0. | 5.191463 | 3.191881 | 1.183910 |
| 30. | 1. | 0. | 2.748482 | -2.382094 | -0.952229 |
| 31. | 1. | 0. | -5.622647 | -2.311876 | 0.593652 |
| 32. | 1. | 0. | -0.868860 | -2.802913 | -0.929627 |
| 33. | 1. | 0. | -2.292839 | -4.791313 | -0.573080 |
| 34. | 1. | 0. | -4.636259 | -4.560290 | 0.187956 |
| 35. | 1. | 0. | -4.945982 | 0.441374 | 0.444706 |
| 36. | 1. | 0. | -0.114895 | -1.397519 | 1.141328 |

| | | | | | |
|---|---|---|---|---|---|
| 37. | 1. | 0. | -0.407564 | 1.155158 | -1.521254 |
| 38. | 1. | 0. | 4.503008 | -1.620615 | 1.421404 |
| 39. | 1. | 0. | 4.628303 | -3.057790 | 0.392393 |
| 40. | 1. | 0. | 6.225964 | -0.936410 | -0.241457 |
| 41. | 1. | 0. | 5.279703 | -1.661648 | -1.557466 |
| 42. | 1. | 0. | 4.488265 | 0.628239 | -1.715768 |
| 43. | 1. | 0. | -4.708618 | 2.593740 | -1.385861 |
| 44. | 1. | 0. | -2.314813 | 4.173967 | -0.779331 |
| 45. | 1. | 0. | -1.056723 | 2.947990 | -0.602277 |
| 46. | 1. | 0. | -2.236109 | 2.822534 | -1.920448 |
| 47. | 1. | 0. | -6.393982 | 3.511711 | 0.079345 |
| 48. | 1. | 0. | -5.259953 | 3.403388 | 1.531119 |
| 49. | 1. | 0. | -3.391955 | 2.136564 | 2.220966 |
| 50. | 1. | 0. | -1.702137 | 2.350819 | 1.752020 |
| 51. | 1. | 0. | -2.811699 | 3.733256 | 1.703341 |
| 52. | 1. | 0. | 3.304192 | 2.417264 | 0.451980 |
| 53. | 1. | 0. | 4.481860 | 2.743509 | -0.820831 |
| 54. | 1. | 0. | 6.253015 | 3.163471 | 0.921144 |
| 55. | 1. | 0. | 4.857846 | 4.234349 | 1.173587 |
| 56. | 1. | 0. | 5.081130 | 2.804617 | 2.201164 |

| **1-4** | | Standard Orientation (Ångstroms) | | | |
|---|---|---|---|---|---|
| Center number | Atom number | Type | X | Y | Z |
| 1. | 6. | 0. | -4.937502 | -2.077982 | 0.366335 |
| 2. | 6. | 0. | -4.027202 | -1.033271 | 0.187246 |
| 3. | 6. | 0. | -2.677887 | -1.242482 | -0.198194 |
| 4. | 6. | 0. | -2.258022 | -2.561440 | -0.456712 |
| 5. | 6. | 0. | -3.157485 | -3.606073 | -0.281443 |
| 6. | 6. | 0. | -4.482352 | -3.370374 | 0.134105 |
| 7. | 7. | 0. | -4.212174 | 0.329096 | 0.299490 |
| 8. | 6. | 0. | -3.045605 | 1.003293 | 0.002894 |
| 9. | 6. | 0. | -2.056042 | 0.067288 | -0.299334 |
| 10. | 6. | 0. | -2.992395 | 2.529090 | 0.021564 |
| 11. | 7. | 0. | 0.506914 | -1.346006 | 0.541846 |
| 12. | 6. | 0. | 0.449137 | -0.266091 | -0.364054 |
| 13. | 6. | 0. | -0.690151 | 0.389818 | -0.682309 |
| 14. | 6. | 0. | 1.588396 | -2.164741 | 0.754228 |
| 15. | 6. | 0. | 2.701369 | -1.933380 | -0.254865 |
| 16. | 7. | 0. | 2.837093 | -0.505634 | -0.581192 |
| 17. | 6. | 0. | 1.755048 | 0.283292 | -0.863886 |

| | | | | | |
|---|---|---|---|---|---|
| 18. | 6. | 0. | 4.117073 | -2.335000 | 0.154174 |
| 19. | 6. | 0. | 4.966750 | -1.462290 | -0.787997 |
| 20. | 6. | 0. | 4.240322 | -0.108966 | -0.788704 |
| 21. | 8. | 0. | 1.843089 | 1.373007 | -1.427655 |
| 22. | 8. | 0. | 1.619780 | -3.024025 | 1.620909 |
| 23. | 6. | 0. | -1.774046 | 2.977062 | 0.820334 |
| 24. | 6. | 0. | -4.234681 | 3.114397 | 0.742482 |
| 25. | 6. | 0. | -0.826302 | 3.830137 | 0.435834 |
| 26. | 6. | 0. | -2.983911 | 3.055717 | -1.431896 |
| 27. | 8. | 0. | 4.766952 | 0.643994 | 0.280503 |
| 28. | 6. | 0. | 4.375531 | 2.018110 | 0.346703 |
| 29. | 6. | 0. | 5.268145 | 2.700361 | 1.369313 |
| 30. | 1. | 0. | 2.413983 | -2.493054 | -1.162941 |
| 31. | 1. | 0. | -5.963931 | -1.886567 | 0.666238 |
| 32. | 1. | 0. | -1.248925 | -2.763610 | -0.797149 |
| 33. | 1. | 0. | -2.834496 | -4.624810 | -0.473046 |
| 34. | 1. | 0. | -5.160458 | -4.208150 | 0.264337 |
| 35. | 1. | 0. | -5.068452 | 0.770358 | 0.590475 |
| 36. | 1. | 0. | -0.295454 | -1.513175 | 1.137602 |
| 37. | 1. | 0. | -0.515109 | 1.291654 | -1.257597 |
| 38. | 1. | 0. | 4.293451 | -2.064281 | 1.198443 |
| 39. | 1. | 0. | 4.290088 | -3.406420 | 0.041186 |
| 40. | 1. | 0. | 6.001178 | -1.339516 | -0.464319 |
| 41. | 1. | 0. | 4.965021 | -1.881384 | -1.800715 |
| 42. | 1. | 0. | 4.318881 | 0.456089 | -1.725604 |
| 43. | 1. | 0. | -1.726632 | 2.555754 | 1.824516 |
| 44. | 1. | 0. | -4.151864 | 4.203285 | 0.778149 |
| 45. | 1. | 0. | -5.163527 | 2.876704 | 0.209902 |
| 46. | 1. | 0. | -4.315183 | 2.753044 | 1.773033 |
| 47. | 1. | 0. | -0.015693 | 4.098316 | 1.105855 |
| 48. | 1. | 0. | -0.799586 | 4.276655 | -0.552830 |
| 49. | 1. | 0. | -2.107487 | 2.711150 | -1.985102 |
| 50. | 1. | 0. | -3.874237 | 2.706344 | -1.962860 |
| 51. | 1. | 0. | -2.988739 | 4.150265 | -1.445957 |
| 52. | 1. | 0. | 3.319783 | 2.103409 | 0.630091 |
| 53. | 1. | 0. | 4.479174 | 2.483070 | -0.644164 |
| 54. | 1. | 0. | 6.319009 | 2.635544 | 1.072226 |
| 55. | 1. | 0. | 4.997694 | 3.756830 | 1.464091 |
| 56. | 1. | 0. | 5.160993 | 2.227650 | 2.350151 |

| **1-5** | Standard Orientation<br>(Ångstroms) |
|---|---|

| Center number | Atom number | Type | X | Y | Z |
|---|---|---|---|---|---|
| 1. | 6. | 0. | -4.106763 | 2.925798 | -0.137625 |
| 2. | 6. | 0. | -3.505320 | 1.665401 | -0.195062 |
| 3. | 6. | 0. | -2.118707 | 1.489976 | -0.441241 |
| 4. | 6. | 0. | -1.326105 | 2.631273 | -0.669160 |
| 5. | 6. | 0. | -1.917431 | 3.887592 | -0.605833 |
| 6. | 6. | 0. | -3.292366 | 4.034670 | -0.336991 |
| 7. | 7. | 0. | -4.052485 | 0.408505 | -0.073279 |
| 8. | 6. | 0. | -3.085159 | -0.564346 | -0.246269 |
| 9. | 6. | 0. | -1.859799 | 0.061433 | -0.456452 |
| 10. | 6. | 0. | -3.495205 | -2.021273 | -0.042476 |
| 11. | 7. | 0. | 0.684541 | 0.712331 | 1.015864 |
| 12. | 6. | 0. | 0.592317 | -0.259442 | -0.000952 |
| 13. | 6. | 0. | -0.551797 | -0.564506 | -0.652736 |
| 14. | 6. | 0. | 1.733739 | 0.870660 | 1.888222 |
| 15. | 6. | 0. | 2.768421 | -0.236458 | 1.780859 |
| 16. | 7. | 0. | 2.932097 | -0.697569 | 0.394664 |
| 17. | 6. | 0. | 1.865497 | -0.940988 | -0.425265 |
| 18. | 6. | 0. | 4.197581 | 0.084474 | 2.214054 |
| 19. | 6. | 0. | 4.991354 | -1.007347 | 1.472931 |
| 20. | 6. | 0. | 4.325105 | -1.072550 | 0.089980 |
| 21. | 8. | 0. | 1.939987 | -1.613250 | -1.451849 |
| 22. | 8. | 0. | 1.785139 | 1.766187 | 2.715921 |
| 23. | 6. | 0. | -4.904781 | -2.193235 | -0.597906 |
| 24. | 6. | 0. | -3.414861 | -2.348485 | 1.466627 |
| 25. | 6. | 0. | -6.014582 | -2.485537 | 0.082567 |
| 26. | 6. | 0. | -2.584088 | -2.999901 | -0.818609 |
| 27. | 8. | 0. | 4.982158 | -0.135260 | -0.730860 |
| 28. | 6. | 0. | 4.665257 | -0.171713 | -2.126001 |
| 29. | 6. | 0. | 5.698656 | 0.671103 | -2.853525 |
| 30. | 1. | 0. | 2.384150 | -1.066430 | 2.400722 |
| 31. | 1. | 0. | -5.170782 | 3.034940 | 0.051473 |
| 32. | 1. | 0. | -0.272045 | 2.533913 | -0.910361 |
| 33. | 1. | 0. | -1.311323 | 4.772339 | -0.774808 |
| 34. | 1. | 0. | -3.725221 | 5.029416 | -0.294167 |
| 35. | 1. | 0. | -5.028500 | 0.193069 | 0.063910 |
| 36. | 1. | 0. | -0.080465 | 1.368025 | 1.127255 |
| 37. | 1. | 0. | -0.432626 | -1.328064 | -1.413789 |
| 38. | 1. | 0. | 4.481604 | 1.078703 | 1.859130 |
| 39. | 1. | 0. | 4.314478 | 0.058173 | 3.298495 |
| 40. | 1. | 0. | 6.055340 | -0.789485 | 1.372104 |

| | | | | | |
|---|---|---|---|---|---|
| 41. | 1. | 0. | 4.879026 | -1.971352 | 1.982216 |
| 42. | 1. | 0. | 4.335930 | -2.063350 | -0.380451 |
| 43. | 1. | 0. | -4.974801 | -2.057890 | -1.677901 |
| 44. | 1. | 0. | -3.718740 | -3.383177 | 1.655750 |
| 45. | 1. | 0. | -2.387544 | -2.221887 | 1.817583 |
| 46. | 1. | 0. | -4.053133 | -1.686178 | 2.058580 |
| 47. | 1. | 0. | -6.966868 | -2.601513 | -0.426344 |
| 48. | 1. | 0. | -6.020088 | -2.639580 | 1.157174 |
| 49. | 1. | 0. | -2.549297 | -2.755197 | -1.884575 |
| 50. | 1. | 0. | -1.563759 | -2.990814 | -0.430506 |
| 51. | 1. | 0. | -2.975143 | -4.016519 | -0.715954 |
| 52. | 1. | 0. | 3.653843 | 0.214483 | -2.299520 |
| 53. | 1. | 0. | 4.677191 | -1.211326 | -2.482824 |
| 54. | 1. | 0. | 5.683933 | 1.701120 | -2.485026 |
| 55. | 1. | 0. | 6.704564 | 0.268613 | -2.702104 |
| 56. | 1. | 0. | 5.487152 | 0.686085 | -3.927331 |

| **1-6** | | Standard Orientation (Ångstroms) | | | |
|---|---|---|---|---|---|
| Center number | Atom number | Type | X | Y | Z |
| 1. | 6. | 0. | 4.459124 | 2.734357 | 0.264641 |
| 2. | 6. | 0. | 3.770656 | 1.519231 | 0.228049 |
| 3. | 6. | 0. | 2.367636 | 1.424467 | 0.420723 |
| 4. | 6. | 0. | 1.654130 | 2.601888 | 0.718734 |
| 5. | 6. | 0. | 2.332839 | 3.814545 | 0.755597 |
| 6. | 6. | 0. | 3.719575 | 3.883422 | 0.520892 |
| 7. | 7. | 0. | 4.242392 | 0.236063 | 0.049277 |
| 8. | 6. | 0. | 3.204997 | -0.674378 | 0.112935 |
| 9. | 6. | 0. | 2.016791 | 0.019946 | 0.323886 |
| 10. | 6. | 0. | 3.463627 | -2.173027 | -0.010776 |
| 11. | 7. | 0. | -0.569679 | 0.970238 | -0.952060 |
| 12. | 6. | 0. | -0.465600 | -0.144801 | -0.094272 |
| 13. | 6. | 0. | 0.691191 | -0.574907 | 0.457269 |
| 14. | 6. | 0. | -1.643900 | 1.279319 | -1.750005 |
| 15. | 6. | 0. | -2.710855 | 0.198962 | -1.758753 |
| 16. | 7. | 0. | -2.831548 | -0.460084 | -0.451022 |
| 17. | 6. | 0. | -1.739002 | -0.856150 | 0.271268 |
| 18. | 6. | 0. | -4.145417 | 0.619655 | -2.071990 |
| 19. | 6. | 0. | -4.942631 | -0.551534 | -1.468528 |
| 20. | 6. | 0. | -4.220958 | -0.843232 | -0.143732 |
| 21. | 8. | 0. | -1.795409 | -1.679358 | 1.182811 |

| | | | | | |
|---|---|---|---|---|---|
| 22. | 8. | 0. | -1.693573 | 2.283860 | -2.442396 |
| 23. | 6. | 0. | 2.460696 | -2.776946 | -0.986466 |
| 24. | 6. | 0. | 3.391602 | -2.814824 | 1.393822 |
| 25. | 6. | 0. | 1.694905 | -3.849216 | -0.791278 |
| 26. | 6. | 0. | 4.873403 | -2.442578 | -0.598789 |
| 27. | 8. | 0. | -4.816007 | -0.025699 | 0.837990 |
| 28. | 6. | 0. | -4.422623 | -0.262198 | 2.193099 |
| 29. | 6. | 0. | -5.404587 | 0.471447 | 3.090546 |
| 30. | 1. | 0. | -2.381996 | -0.538302 | -2.513297 |
| 31. | 1. | 0. | 5.533359 | 2.779151 | 0.109225 |
| 32. | 1. | 0. | 0.592830 | 2.566321 | 0.943493 |
| 33. | 1. | 0. | 1.785649 | 4.725162 | 0.979218 |
| 34. | 1. | 0. | 4.221407 | 4.845480 | 0.554976 |
| 35. | 1. | 0. | 5.200513 | -0.003075 | -0.143878 |
| 36. | 1. | 0. | 0.217077 | 1.605050 | -1.021138 |
| 37. | 1. | 0. | 0.576439 | -1.464854 | 1.066727 |
| 38. | 1. | 0. | -4.381974 | 1.554890 | -1.557539 |
| 39. | 1. | 0. | -4.308601 | 0.762352 | -3.141316 |
| 40. | 1. | 0. | -5.993783 | -0.324175 | -1.286435 |
| 41. | 1. | 0. | -4.883389 | -1.429291 | -2.122284 |
| 42. | 1. | 0. | -4.243005 | -1.893187 | 0.172864 |
| 43. | 1. | 0. | 2.423752 | -2.271884 | -1.951552 |
| 44. | 1. | 0. | 3.618601 | -3.884523 | 1.343426 |
| 45. | 1. | 0. | 4.122103 | -2.345091 | 2.058843 |
| 46. | 1. | 0. | 2.402900 | -2.695858 | 1.843070 |
| 47. | 1. | 0. | 1.039664 | -4.214911 | -1.575665 |
| 48. | 1. | 0. | 1.671841 | -4.393624 | 0.147154 |
| 49. | 1. | 0. | 4.998532 | -1.982870 | -1.584724 |
| 50. | 1. | 0. | 5.666441 | -2.077636 | 0.065101 |
| 51. | 1. | 0. | 5.018262 | -3.519433 | -0.713350 |
| 52. | 1. | 0. | -3.399216 | 0.093624 | 2.362114 |
| 53. | 1. | 0. | -4.424649 | -1.341789 | 2.398914 |
| 54. | 1. | 0. | -5.400232 | 1.543188 | 2.870660 |
| 55. | 1. | 0. | -6.421510 | 0.096589 | 2.941642 |
| 56. | 1. | 0. | -5.132854 | 0.334062 | 4.141902 |

| **1-7** | | Standard Orientation (Ångstroms) | | | |
|---|---|---|---|---|---|
| Center number | Atom number | Type | X | Y | Z |
| 1. | 6. | 0. | -4.887181 | -2.163875 | 0.352398 |
| 2. | 6. | 0. | -3.992895 | -1.098473 | 0.220733 |

| | | | | | |
|---|---|---|---|---|---|
| 3. | 6. | 0. | -2.645215 | -1.268204 | -0.189149 |
| 4. | 6. | 0. | -2.209961 | -2.565682 | -0.520646 |
| 5. | 6. | 0. | -3.093203 | -3.630700 | -0.392106 |
| 6. | 6. | 0. | -4.416983 | -3.435611 | 0.047503 |
| 7. | 7. | 0. | -4.194915 | 0.253392 | 0.407820 |
| 8. | 6. | 0. | -3.042151 | 0.958847 | 0.132417 |
| 9. | 6. | 0. | -2.043046 | 0.053439 | -0.229887 |
| 10. | 6. | 0. | -2.992602 | 2.479850 | 0.262851 |
| 11. | 7. | 0. | 0.549759 | -1.358935 | 0.518822 |
| 12. | 6. | 0. | 0.468103 | -0.225162 | -0.317148 |
| 13. | 6. | 0. | -0.685915 | 0.422336 | -0.599500 |
| 14. | 6. | 0. | 1.640983 | -2.181341 | 0.656058 |
| 15. | 6. | 0. | 2.734328 | -1.867792 | -0.352528 |
| 16. | 7. | 0. | 2.856177 | -0.418062 | -0.569937 |
| 17. | 6. | 0. | 1.764528 | 0.380430 | -0.776990 |
| 18. | 6. | 0. | 4.159287 | -2.290955 | -0.001479 |
| 19. | 6. | 0. | 4.987948 | -1.339382 | -0.884224 |
| 20. | 6. | 0. | 4.253961 | 0.004364 | -0.762919 |
| 21. | 8. | 0. | 1.836871 | 1.515663 | -1.244941 |
| 22. | 8. | 0. | 1.693597 | -3.099170 | 1.459184 |
| 23. | 6. | 0. | -2.707693 | 3.063290 | -1.118257 |
| 24. | 6. | 0. | -1.934006 | 2.881555 | 1.312264 |
| 25. | 6. | 0. | -1.701628 | 3.865941 | -1.464981 |
| 26. | 6. | 0. | -4.362833 | 3.042001 | 0.716415 |
| 27. | 8. | 0. | 4.792666 | 0.669512 | 0.357006 |
| 28. | 6. | 0. | 4.396696 | 2.031435 | 0.542158 |
| 29. | 6. | 0. | 5.307408 | 2.634469 | 1.598017 |
| 30. | 1. | 0. | 2.432227 | -2.357461 | -1.295532 |
| 31. | 1. | 0. | -5.912892 | -2.003216 | 0.672134 |
| 32. | 1. | 0. | -1.201554 | -2.734751 | -0.880718 |
| 33. | 1. | 0. | -2.758333 | -4.633385 | -0.639733 |
| 34. | 1. | 0. | -5.082407 | -4.288487 | 0.139614 |
| 35. | 1. | 0. | -5.058823 | 0.668434 | 0.714421 |
| 36. | 1. | 0. | -0.245792 | -1.579326 | 1.106357 |
| 37. | 1. | 0. | -0.545160 | 1.357460 | -1.132393 |
| 38. | 1. | 0. | 4.352790 | -2.104660 | 1.058113 |
| 39. | 1. | 0. | 4.336369 | -3.348507 | -0.203738 |
| 40. | 1. | 0. | 6.027020 | -1.236090 | -0.568806 |
| 41. | 1. | 0. | 4.971148 | -1.675195 | -1.927396 |
| 42. | 1. | 0. | 4.314790 | 0.645480 | -1.650844 |
| 43. | 1. | 0. | -3.440493 | 2.781385 | -1.874838 |
| 44. | 1. | 0. | -1.901209 | 3.969942 | 1.424312 |

| | | | | | |
|---|---|---|---|---|---|
| 45. | 1. | 0. | -2.186803 | 2.447629 | 2.284335 |
| 46. | 1. | 0. | -0.937021 | 2.531836 | 1.037419 |
| 47. | 1. | 0. | -1.609785 | 4.234802 | -2.481842 |
| 48. | 1. | 0. | -0.928635 | 4.174960 | -0.769316 |
| 49. | 1. | 0. | -5.158115 | 2.813801 | -0.001977 |
| 50. | 1. | 0. | -4.650592 | 2.654312 | 1.700573 |
| 51. | 1. | 0. | -4.299724 | 4.129987 | 0.795168 |
| 52. | 1. | 0. | 3.346807 | 2.086732 | 0.853813 |
| 53. | 1. | 0. | 4.477229 | 2.575091 | -0.409965 |
| 54. | 1. | 0. | 5.223340 | 2.083049 | 2.539190 |
| 55. | 1. | 0. | 6.352004 | 2.600773 | 1.274812 |
| 56. | 1. | 0. | 5.033814 | 3.677921 | 1.783905 |

| **1-8** | | Standard Orientation (Ångstroms) | | | |
|---|---|---|---|---|---|
| Center number | Atom number | Type | X | Y | Z |
| 1. | 6. | 0. | 4.447234 | 2.710820 | 0.475628 |
| 2. | 6. | 0. | 3.759757 | 1.506330 | 0.307429 |
| 3. | 6. | 0. | 2.359980 | 1.385041 | 0.509477 |
| 4. | 6. | 0. | 1.648960 | 2.518605 | 0.950184 |
| 5. | 6. | 0. | 2.326558 | 3.720848 | 1.117049 |
| 6. | 6. | 0. | 3.710013 | 3.820285 | 0.873622 |
| 7. | 7. | 0. | 4.230785 | 0.254576 | -0.028235 |
| 8. | 6. | 0. | 3.197529 | -0.661709 | -0.049340 |
| 9. | 6. | 0. | 2.010611 | -0.000535 | 0.259832 |
| 10. | 6. | 0. | 3.442264 | -2.130048 | -0.388807 |
| 11. | 7. | 0. | -0.589810 | 1.075175 | -0.874206 |
| 12. | 6. | 0. | -0.473834 | -0.132829 | -0.155012 |
| 13. | 6. | 0. | 0.690276 | -0.615293 | 0.337027 |
| 14. | 6. | 0. | -1.670185 | 1.469380 | -1.625312 |
| 15. | 6. | 0. | -2.730035 | 0.390073 | -1.756646 |
| 16. | 7. | 0. | -2.840309 | -0.417879 | -0.534458 |
| 17. | 6. | 0. | -1.742118 | -0.888643 | 0.132769 |
| 18. | 6. | 0. | -4.168747 | 0.834702 | -2.013317 |
| 19. | 6. | 0. | -4.956347 | -0.402986 | -1.544666 |
| 20. | 6. | 0. | -4.226380 | -0.838983 | -0.265067 |
| 21. | 8. | 0. | -1.789992 | -1.812234 | 0.942643 |
| 22. | 8. | 0. | -1.729837 | 2.548511 | -2.193346 |
| 23. | 6. | 0. | 3.042958 | -2.962764 | 0.825812 |
| 24. | 6. | 0. | 4.944911 | -2.398424 | -0.649557 |
| 25. | 6. | 0. | 2.150685 | -3.951876 | 0.869270 |

| | | | | | |
|---|---|---|---|---|---|
| 26. | 6. | 0. | 2.652669 | -2.509429 | -1.659694 |
| 27. | 8. | 0. | -4.818809 | -0.139499 | 0.805405 |
| 28. | 6. | 0. | -4.417308 | -0.526070 | 2.123231 |
| 29. | 6. | 0. | -5.399577 | 0.093317 | 3.102718 |
| 30. | 1. | 0. | -2.399236 | -0.251446 | -2.593442 |
| 31. | 1. | 0. | 5.519020 | 2.776477 | 0.310969 |
| 32. | 1. | 0. | 0.590612 | 2.454921 | 1.183035 |
| 33. | 1. | 0. | 1.781215 | 4.598227 | 1.450921 |
| 34. | 1. | 0. | 4.211025 | 4.773359 | 1.012088 |
| 35. | 1. | 0. | 5.190216 | 0.037846 | -0.240400 |
| 36. | 1. | 0. | 0.192671 | 1.719019 | -0.870724 |
| 37. | 1. | 0. | 0.598246 | -1.571123 | 0.843881 |
| 38. | 1. | 0. | -4.407845 | 1.703159 | -1.393792 |
| 39. | 1. | 0. | -4.338120 | 1.098312 | -3.058416 |
| 40. | 1. | 0. | -6.007963 | -0.204702 | -1.333636 |
| 41. | 1. | 0. | -4.894604 | -1.199965 | -2.294554 |
| 42. | 1. | 0. | -4.243000 | -1.918023 | -0.068756 |
| 43. | 1. | 0. | 3.578898 | -2.697191 | 1.737385 |
| 44. | 1. | 0. | 5.090150 | -3.457313 | -0.877033 |
| 45. | 1. | 0. | 5.313798 | -1.822364 | -1.506241 |
| 46. | 1. | 0. | 5.561042 | -2.166255 | 0.226242 |
| 47. | 1. | 0. | 1.957615 | -4.487914 | 1.793235 |
| 48. | 1. | 0. | 1.573961 | -4.260413 | 0.003406 |
| 49. | 1. | 0. | 1.580206 | -2.351947 | -1.528218 |
| 50. | 1. | 0. | 2.981803 | -1.894866 | -2.502840 |
| 51. | 1. | 0. | 2.821949 | -3.559677 | -1.917791 |
| 52. | 1. | 0. | -3.395421 | -0.184595 | 2.327551 |
| 53. | 1. | 0. | -4.411210 | -1.622057 | 2.204659 |
| 54. | 1. | 0. | -5.402356 | 1.183266 | 3.007924 |
| 55. | 1. | 0. | -6.414936 | -0.268222 | 2.915220 |
| 56. | 1. | 0. | -5.122537 | -0.162720 | 4.130224 |

| **1-9** | | Standard Orientation (Ångstroms) | | | |
|---|---|---|---|---|---|
| Center number | Atom number | Type | X | Y | Z |
| 1. | 6. | 0. | -4.588037 | -2.419732 | 0.278799 |
| 2. | 6. | 0. | -3.768507 | -1.303628 | 0.086975 |
| 3. | 6. | 0. | -2.414967 | -1.406838 | -0.324098 |
| 4. | 6. | 0. | -1.887787 | -2.686642 | -0.579044 |
| 5. | 6. | 0. | -2.694809 | -3.800939 | -0.385200 |
| 6. | 6. | 0. | -4.030085 | -3.670832 | 0.045032 |

| | | | | | |
|---|---|---|---|---|---|
| 7. | 7. | 0. | -4.051912 | 0.038637 | 0.205620 |
| 8. | 6. | 0. | -2.951855 | 0.803225 | -0.129541 |
| 9. | 6. | 0. | -1.898045 | -0.053667 | -0.448370 |
| 10. | 6. | 0. | -3.060761 | 2.316985 | 0.053534 |
| 11. | 7. | 0. | 0.707849 | -1.209425 | 0.579839 |
| 12. | 6. | 0. | 0.618122 | -0.222906 | -0.422612 |
| 13. | 6. | 0. | -0.545666 | 0.325804 | -0.838690 |
| 14. | 6. | 0. | 1.827440 | -1.939936 | 0.895412 |
| 15. | 6. | 0. | 2.963717 | -1.724309 | -0.091124 |
| 16. | 7. | 0. | 3.023103 | -0.326358 | -0.544973 |
| 17. | 6. | 0. | 1.907055 | 0.363410 | -0.928805 |
| 18. | 6. | 0. | 4.384723 | -1.993994 | 0.400100 |
| 19. | 6. | 0. | 5.214925 | -1.154666 | -0.588725 |
| 20. | 6. | 0. | 4.406814 | 0.143156 | -0.736117 |
| 21. | 8. | 0. | 1.946223 | 1.407612 | -1.577921 |
| 22. | 8. | 0. | 1.874863 | -2.724824 | 1.828764 |
| 23. | 6. | 0. | -4.477664 | 2.731507 | -0.329881 |
| 24. | 6. | 0. | -2.103021 | 3.104514 | -0.871218 |
| 25. | 6. | 0. | -5.421376 | 3.231618 | 0.469767 |
| 26. | 6. | 0. | -2.728152 | 2.656246 | 1.525370 |
| 27. | 8. | 0. | 4.840222 | 1.017628 | 0.280386 |
| 28. | 6. | 0. | 4.370893 | 2.367154 | 0.207409 |
| 29. | 6. | 0. | 5.190292 | 3.191440 | 1.185644 |
| 30. | 1. | 0. | 2.748150 | -2.381256 | -0.952654 |
| 31. | 1. | 0. | -5.621845 | -2.312725 | 0.594312 |
| 32. | 1. | 0. | -0.868139 | -2.802764 | -0.929519 |
| 33. | 1. | 0. | -2.291594 | -4.791464 | -0.572643 |
| 34. | 1. | 0. | -4.634988 | -4.560943 | 0.188615 |
| 35. | 1. | 0. | -4.945960 | 0.440670 | 0.444706 |
| 36. | 1. | 0. | -0.114616 | -1.396910 | 1.141454 |
| 37. | 1. | 0. | -0.407721 | 1.155184 | -1.521670 |
| 38. | 1. | 0. | 4.503570 | -1.620527 | 1.420554 |
| 39. | 1. | 0. | 4.628341 | -3.057521 | 0.391190 |
| 40. | 1. | 0. | 6.226068 | -0.936263 | -0.242815 |
| 41. | 1. | 0. | 5.279170 | -1.661066 | -1.558611 |
| 42. | 1. | 0. | 4.487958 | 0.629140 | -1.716009 |
| 43. | 1. | 0. | -4.708369 | 2.592978 | -1.386867 |
| 44. | 1. | 0. | -2.315221 | 4.173842 | -0.778983 |
| 45. | 1. | 0. | -1.057020 | 2.948006 | -0.601513 |
| 46. | 1. | 0. | -2.235857 | 2.822504 | -1.920169 |
| 47. | 1. | 0. | -6.394900 | 3.510260 | 0.077438 |
| 48. | 1. | 0. | -5.261667 | 3.402222 | 1.529859 |

| | | | | | |
|---|---|---|---|---|---|
| 49. | 1. | 0. | -3.393580 | 2.136352 | 2.220676 |
| 50. | 1. | 0. | -1.703550 | 2.351010 | 1.752650 |
| 51. | 1. | 0. | -2.813448 | 3.733159 | 1.703258 |
| 52. | 1. | 0. | 3.303522 | 2.415725 | 0.453642 |
| 53. | 1. | 0. | 4.480362 | 2.744289 | -0.819317 |
| 54. | 1. | 0. | 5.080622 | 2.803068 | 2.202545 |
| 55. | 1. | 0. | 6.251785 | 3.164296 | 0.922522 |
| 56. | 1. | 0. | 4.855690 | 4.233602 | 1.176427 |

| **1-10** | | Standard Orientation (Ångstroms) | | | |
|---|---|---|---|---|---|
| Center number | Atom number | Type | X | Y | Z |
| 1. | 6. | 0. | -4.769690 | -2.296893 | -0.006026 |
| 2. | 6. | 0. | -3.891289 | -1.210373 | -0.036278 |
| 3. | 6. | 0. | -2.515839 | -1.341468 | -0.361420 |
| 4. | 6. | 0. | -2.031482 | -2.616489 | -0.710265 |
| 5. | 6. | 0. | -2.899330 | -3.701406 | -0.682019 |
| 6. | 6. | 0. | -4.253666 | -3.546751 | -0.326874 |
| 7. | 7. | 0. | -4.127449 | 0.128003 | 0.191575 |
| 8. | 6. | 0. | -2.972192 | 0.861108 | 0.026318 |
| 9. | 6. | 0. | -1.936410 | -0.009815 | -0.312163 |
| 10. | 6. | 0. | -3.017914 | 2.380719 | 0.190636 |
| 11. | 7. | 0. | 0.629142 | -1.367024 | 0.584299 |
| 12. | 6. | 0. | 0.578620 | -0.248006 | -0.269316 |
| 13. | 6. | 0. | -0.567226 | 0.377052 | -0.621383 |
| 14. | 6. | 0. | 1.735639 | -2.132935 | 0.851239 |
| 15. | 6. | 0. | 2.926208 | -1.809623 | -0.037994 |
| 16. | 7. | 0. | 2.985650 | -0.387186 | -0.398935 |
| 17. | 6. | 0. | 1.885638 | 0.372294 | -0.668621 |
| 18. | 6. | 0. | 4.310592 | -2.075351 | 0.556401 |
| 19. | 6. | 0. | 5.198234 | -1.158382 | -0.302195 |
| 20. | 6. | 0. | 4.366499 | 0.126758 | -0.467289 |
| 21. | 8. | 0. | 1.950914 | 1.501762 | -1.156283 |
| 22. | 8. | 0. | 1.741539 | -3.033780 | 1.675381 |
| 23. | 6. | 0. | -4.243691 | 2.746312 | 1.020975 |
| 24. | 6. | 0. | -3.050350 | 3.035477 | -1.209188 |
| 25. | 6. | 0. | -5.339742 | 3.387895 | 0.612813 |
| 26. | 6. | 0. | -1.788143 | 2.900012 | 0.980050 |
| 27. | 8. | 0. | 4.641428 | 1.006058 | 0.599416 |
| 28. | 6. | 0. | 4.682753 | 2.405984 | 0.276936 |
| 29. | 6. | 0. | 6.066749 | 2.837760 | -0.195966 |

| | | | | | |
|---|---|---|---|---|---|
| 30. | 1. | 0. | 2.799525 | -2.419027 | -0.950443 |
| 31. | 1. | 0. | -5.817482 | -2.167433 | 0.249387 |
| 32. | 1. | 0. | -0.997371 | -2.752321 | -1.006966 |
| 33. | 1. | 0. | -2.528404 | -4.687816 | -0.943455 |
| 34. | 1. | 0. | -4.906138 | -4.414417 | -0.313117 |
| 35. | 1. | 0. | -5.012117 | 0.544591 | 0.439561 |
| 36. | 1. | 0. | -0.209624 | -1.614155 | 1.096947 |
| 37. | 1. | 0. | -0.400394 | 1.293724 | -1.177580 |
| 38. | 1. | 0. | 4.328049 | -1.763258 | 1.604271 |
| 39. | 1. | 0. | 4.584192 | -3.130304 | 0.507497 |
| 40. | 1. | 0. | 6.163533 | -0.928846 | 0.150742 |
| 41. | 1. | 0. | 5.373723 | -1.613041 | -1.284398 |
| 42. | 1. | 0. | 4.519654 | 0.629302 | -1.428367 |
| 43. | 1. | 0. | -4.179217 | 2.441859 | 2.066671 |
| 44. | 1. | 0. | -3.103909 | 4.125385 | -1.122517 |
| 45. | 1. | 0. | -2.146515 | 2.783955 | -1.769625 |
| 46. | 1. | 0. | -3.908492 | 2.690296 | -1.793158 |
| 47. | 1. | 0. | -6.144347 | 3.615147 | 1.305951 |
| 48. | 1. | 0. | -5.473769 | 3.726543 | -0.409706 |
| 49. | 1. | 0. | -1.723434 | 2.421480 | 1.962072 |
| 50. | 1. | 0. | -0.851966 | 2.710468 | 0.453127 |
| 51. | 1. | 0. | -1.882024 | 3.979886 | 1.131972 |
| 52. | 1. | 0. | 4.423583 | 2.922186 | 1.207040 |
| 53. | 1. | 0. | 3.909840 | 2.638146 | -0.462125 |
| 54. | 1. | 0. | 6.825721 | 2.586862 | 0.551306 |
| 55. | 1. | 0. | 6.339786 | 2.348582 | -1.137213 |
| 56. | 1. | 0. | 6.091326 | 3.920072 | -0.364793 |

| **1-11** | | Standard Orientation (Ångstroms) | | | |
|---|---|---|---|---|---|
| Center number | Atom number | Type | X | Y | Z |
| 1. | 6. | 0. | -4.827941 | 2.154402 | -0.425334 |
| 2. | 6. | 0. | -3.942167 | 1.082638 | -0.286048 |
| 3. | 6. | 0. | -2.596855 | 1.243045 | 0.135264 |
| 4. | 6. | 0. | -2.155132 | 2.537696 | 0.469771 |
| 5. | 6. | 0. | -3.029678 | 3.608747 | 0.333180 |
| 6. | 6. | 0. | -4.351268 | 3.422789 | -0.117304 |
| 7. | 7. | 0. | -4.151877 | -0.267402 | -0.473251 |
| 8. | 6. | 0. | -3.007518 | -0.981946 | -0.186967 |
| 9. | 6. | 0. | -2.004708 | -0.082944 | 0.181446 |
| 10. | 6. | 0. | -2.968557 | -2.495704 | -0.340745 |

| | | | | | |
|---|---|---|---|---|---|
| 11. | 7. | 0. | 0.605018 | 1.308665 | -0.541675 |
| 12. | 6. | 0. | 0.506974 | 0.180134 | 0.298928 |
| 13. | 6. | 0. | -0.654080 | -0.459552 | 0.569715 |
| 14. | 6. | 0. | 1.703065 | 2.123563 | -0.671541 |
| 15. | 6. | 0. | 2.783355 | 1.809742 | 0.350805 |
| 16. | 7. | 0. | 2.893043 | 0.361019 | 0.580231 |
| 17. | 6. | 0. | 1.794336 | -0.429574 | 0.779212 |
| 18. | 6. | 0. | 4.214897 | 2.220734 | 0.011935 |
| 19. | 6. | 0. | 5.027666 | 1.270006 | 0.910216 |
| 20. | 6. | 0. | 4.286012 | -0.069664 | 0.790474 |
| 21. | 8. | 0. | 1.853513 | -1.561822 | 1.255554 |
| 22. | 8. | 0. | 1.769732 | 3.036238 | -1.479341 |
| 23. | 6. | 0. | -2.750771 | -3.207026 | 0.992497 |
| 24. | 6. | 0. | -1.831215 | -2.893864 | -1.319070 |
| 25. | 6. | 0. | -2.853462 | -2.693562 | 2.217071 |
| 26. | 6. | 0. | -4.300206 | -3.034982 | -0.923506 |
| 27. | 8. | 0. | 4.831210 | -0.745865 | -0.319455 |
| 28. | 6. | 0. | 4.429182 | -2.107009 | -0.498809 |
| 29. | 6. | 0. | 5.346611 | -2.722655 | -1.541450 |
| 30. | 1. | 0. | 2.474576 | 2.308566 | 1.286829 |
| 31. | 1. | 0. | -5.852156 | 2.000716 | -0.753142 |
| 32. | 1. | 0. | -1.148666 | 2.699979 | 0.838315 |
| 33. | 1. | 0. | -2.689815 | 4.609198 | 0.583037 |
| 34. | 1. | 0. | -5.009813 | 4.280340 | -0.215182 |
| 35. | 1. | 0. | -5.017110 | -0.677284 | -0.782797 |
| 36. | 1. | 0. | -0.182496 | 1.530827 | -1.139318 |
| 37. | 1. | 0. | -0.525421 | -1.390941 | 1.112236 |
| 38. | 1. | 0. | 4.418400 | 2.025411 | -1.044152 |
| 39. | 1. | 0. | 4.397003 | 3.278510 | 0.208469 |
| 40. | 1. | 0. | 6.069292 | 1.157585 | 0.606576 |
| 41. | 1. | 0. | 5.002073 | 1.613359 | 1.950756 |
| 42. | 1. | 0. | 4.333358 | -0.705061 | 1.683289 |
| 43. | 1. | 0. | -2.525587 | -4.268698 | 0.883188 |
| 44. | 1. | 0. | -1.813217 | -3.981326 | -1.450039 |
| 45. | 1. | 0. | -1.991557 | -2.434858 | -2.299621 |
| 46. | 1. | 0. | -0.853486 | -2.579416 | -0.950420 |
| 47. | 1. | 0. | -2.708312 | -3.314636 | 3.095523 |
| 48. | 1. | 0. | -3.069300 | -1.643183 | 2.387491 |
| 49. | 1. | 0. | -5.149043 | -2.813981 | -0.267981 |
| 50. | 1. | 0. | -4.495024 | -2.627840 | -1.922466 |
| 51. | 1. | 0. | -4.245174 | -4.122831 | -1.019667 |
| 52. | 1. | 0. | 3.382112 | -2.158662 | -0.820206 |

| | | | | | |
|---|---|---|---|---|---|
| 53. | 1. | 0. | 4.497409 | -2.644236 | 0.457879 |
| 54. | 1. | 0. | 5.274698 | -2.177661 | -2.487372 |
| 55. | 1. | 0. | 6.388240 | -2.692484 | -1.208462 |
| 56. | 1. | 0. | 5.068832 | -3.765857 | -1.722403 |

| **1-12** | | Standard Orientation (Ångstroms) | | | |
|---|---|---|---|---|---|
| Center number | Atom number | Type | X | Y | Z |
| 1. | 6. | 0. | -4.387672 | 2.626624 | -0.751606 |
| 2. | 6. | 0. | -3.685214 | 1.445670 | -0.497304 |
| 3. | 6. | 0. | -2.277335 | 1.342075 | -0.649179 |
| 4. | 6. | 0. | -1.571173 | 2.464273 | -1.124571 |
| 5. | 6. | 0. | -2.264399 | 3.642257 | -1.377410 |
| 6. | 6. | 0. | -3.657136 | 3.726923 | -1.185305 |
| 7. | 7. | 0. | -4.139897 | 0.206354 | -0.104856 |
| 8. | 6. | 0. | -3.091158 | -0.684062 | 0.004741 |
| 9. | 6. | 0. | -1.908653 | -0.021312 | -0.313515 |
| 10. | 6. | 0. | -3.378049 | -2.137315 | 0.380626 |
| 11. | 7. | 0. | 0.628253 | 1.134889 | 0.849813 |
| 12. | 6. | 0. | 0.571212 | -0.075625 | 0.132693 |
| 13. | 6. | 0. | -0.568548 | -0.600273 | -0.369903 |
| 14. | 6. | 0. | 1.700822 | 1.609358 | 1.561576 |
| 15. | 6. | 0. | 2.845586 | 0.619254 | 1.692229 |
| 16. | 7. | 0. | 2.943452 | -0.296900 | 0.550367 |
| 17. | 6. | 0. | 1.868525 | -0.784239 | -0.133544 |
| 18. | 6. | 0. | 4.254239 | 1.209751 | 1.784561 |
| 19. | 6. | 0. | 5.118933 | 0.004551 | 1.376945 |
| 20. | 6. | 0. | 4.337184 | -0.652258 | 0.223684 |
| 21. | 8. | 0. | 1.951897 | -1.707415 | -0.943705 |
| 22. | 8. | 0. | 1.703147 | 2.698981 | 2.112994 |
| 23. | 6. | 0. | -4.721462 | -2.203253 | 1.098449 |
| 24. | 6. | 0. | -2.321044 | -2.675351 | 1.378045 |
| 25. | 6. | 0. | -5.850034 | -2.762811 | 0.659351 |
| 26. | 6. | 0. | -3.361097 | -2.997365 | -0.903436 |
| 27. | 8. | 0. | 4.732393 | -0.083772 | -1.003711 |
| 28. | 6. | 0. | 4.848112 | -0.987157 | -2.115530 |
| 29. | 6. | 0. | 6.228019 | -1.632684 | -2.180853 |
| 30. | 1. | 0. | 2.636708 | 0.044493 | 2.612233 |
| 31. | 1. | 0. | -5.465136 | 2.679836 | -0.624588 |
| 32. | 1. | 0. | -0.503811 | 2.409370 | -1.314522 |
| 33. | 1. | 0. | -1.723909 | 4.511871 | -1.738519 |

| | | | | | |
|---|---|---|---|---|---|
| 34. | 1. | 0. | -4.169961 | 4.661413 | -1.391228 |
| 35. | 1. | 0. | -5.098357 | -0.047004 | 0.081184 |
| 36. | 1. | 0. | -0.187670 | 1.736407 | 0.845425 |
| 37. | 1. | 0. | -0.423261 | -1.543103 | -0.889183 |
| 38. | 1. | 0. | 4.361287 | 2.026099 | 1.064626 |
| 39. | 1. | 0. | 4.473538 | 1.597570 | 2.780283 |
| 40. | 1. | 0. | 6.123386 | 0.272740 | 1.047061 |
| 41. | 1. | 0. | 5.203720 | -0.698922 | 2.213735 |
| 42. | 1. | 0. | 4.438994 | -1.742396 | 0.190625 |
| 43. | 1. | 0. | -4.721670 | -1.738007 | 2.085296 |
| 44. | 1. | 0. | -2.576090 | -3.700291 | 1.664979 |
| 45. | 1. | 0. | -1.320621 | -2.679896 | 0.942606 |
| 46. | 1. | 0. | -2.289426 | -2.062738 | 2.284239 |
| 47. | 1. | 0. | -6.746057 | -2.768478 | 1.273016 |
| 48. | 1. | 0. | -5.925054 | -3.248647 | -0.308443 |
| 49. | 1. | 0. | -4.093594 | -2.641781 | -1.633774 |
| 50. | 1. | 0. | -2.376383 | -2.954703 | -1.375616 |
| 51. | 1. | 0. | -3.582008 | -4.043950 | -0.670293 |
| 52. | 1. | 0. | 4.676004 | -0.371611 | -3.004346 |
| 53. | 1. | 0. | 4.050479 | -1.734832 | -2.067727 |
| 54. | 1. | 0. | 6.415193 | -2.269114 | -1.308994 |
| 55. | 1. | 0. | 6.312936 | -2.260391 | -3.074885 |
| 56. | 1. | 0. | 7.010693 | -0.868870 | -2.219541 |

**Table S4.** Energies of conformers of **4b** with Boltzmann distribution over 1% at MMFF.

| Label | E (kcal/mol) | rel. E (kcal/mol) | Boltzmann Dist |
|---|---|---|---|
| M0001 | 89.86 | 0.00 | 0.245 |
| M0002 | 90.21 | 0.35 | 0.136 |
| M0003 | 90.21 | 0.35 | 0.135 |
| M0004 | 90.25 | 0.39 | 0.127 |
| M0005 | 90.57 | 0.70 | 0.075 |
| M0006 | 90.61 | 0.75 | 0.069 |
| M0007 | 90.83 | 0.97 | 0.048 |
| M0008 | 90.90 | 1.04 | 0.042 |
| M0009 | 91.66 | 1.79 | 0.012 |
| M0010 | 91.68 | 1.82 | 0.011 |
| M0011 | 91.75 | 1.89 | 0.010 |

**Table S5.** Gibbs free energies[a] and equilibrium populations[b] of low-energy conformers of **4b**.

| Conformers | In MeOH | |
|---|---|---|
| | *G* | *P* (%) |

| | | |
|---|---|---|
| **4b-1** | -804640.44611847 | 24.39 |
| **4b-2** | -804639.2620071 | 3.30 |
| **4b-3** | -804640.39466265 | 22.36 |
| **4b-4** | -804639.30969786 | 3.58 |
| **4b-5** | -804638.16637464 | 0.52 |
| **4b-6** | -804638.93507439 | 1.90 |
| **4b-7** | -804639.94034541 | 10.38 |
| **4b-8** | -804639.26702718 | 3.33 |
| **4b-9** | -804638.92001415 | 1.85 |
| **4b-10** | -804639.35236854 | 3.84 |
| **4b-11** | -804640.44988353 | 24.55 |

[a]B3LYP/6-31G(d,p), in kcal/mol. [b]From *G* values at 298.15K.

**Table S6** Cartesian coordinates for the low-energy reoptimized MMFF conformers of **4b** at B3LYP/6-31+G(d,p) level of theory in gas.

| **4b-1** | | Standard Orientation (Ångstroms) | | | |
|---|---|---|---|---|---|
| Center number | Atomic number | Atomic Type | X | Y | Z |
| 1. | 6. | 0. | 3.391808 | 3.286226 | 0.208876 |
| 2. | 6. | 0. | 3.050587 | 1.935357 | 0.098611 |
| 3. | 6. | 0. | 1.725155 | 1.495758 | -0.154510 |
| 4. | 6. | 0. | 0.723707 | 2.465921 | -0.349546 |
| 5. | 6. | 0. | 1.056765 | 3.810687 | -0.241802 |
| 6. | 6. | 0. | 2.374858 | 4.218819 | 0.042581 |
| 7. | 7. | 0. | 3.842269 | 0.809781 | 0.171997 |
| 8. | 6. | 0. | 3.089363 | -0.328728 | -0.022081 |
| 9. | 6. | 0. | 1.760946 | 0.044715 | -0.221910 |
| 10. | 6. | 0. | 3.780648 | -1.692157 | -0.030678 |
| 11. | 7. | 0. | -1.030545 | 0.133642 | 0.940125 |
| 12. | 6. | 0. | -0.615578 | -0.776495 | -0.048232 |
| 13. | 6. | 0. | 0.649054 | -0.848001 | -0.518715 |
| 14. | 6. | 0. | -2.320059 | 0.350003 | 1.342105 |
| 15. | 6. | 0. | -3.369775 | -0.320914 | 0.450743 |
| 16. | 7. | 0. | -2.891543 | -1.586151 | -0.058903 |
| 17. | 6. | 0. | -1.612700 | -1.802434 | -0.492481 |
| 18. | 6. | 0. | -4.670572 | -0.725095 | 1.154748 |
| 19. | 6. | 0. | -5.229455 | -1.809232 | 0.213846 |
| 20. | 6. | 0. | -3.978012 | -2.541408 | -0.314519 |
| 21. | 8. | 0. | -1.300157 | -2.795729 | -1.144250 |
| 22. | 8. | 0. | -2.616327 | 1.078557 | 2.279977 |
| 23. | 6. | 0. | 5.104946 | -1.574114 | 0.715651 |

| | | | | | |
|---|---|---|---|---|---|
| 24. | 6. | 0. | 3.976974 | -2.142480 | -1.496249 |
| 25. | 6. | 0. | 6.335060 | -1.654775 | 0.205853 |
| 26. | 6. | 0. | 2.943767 | -2.752638 | 0.730418 |
| 27. | 8. | 0. | -3.591119 | 0.519951 | -0.697576 |
| 28. | 6. | 0. | -4.130967 | 1.829748 | -0.476521 |
| 29. | 6. | 0. | -4.112304 | 2.552432 | -1.813379 |
| 30. | 1. | 0. | 4.414124 | 3.595178 | 0.407070 |
| 31. | 1. | 0. | -0.291135 | 2.169861 | -0.592381 |
| 32. | 1. | 0. | 0.287800 | 4.563873 | -0.384953 |
| 33. | 1. | 0. | 2.602093 | 5.277523 | 0.122053 |
| 34. | 1. | 0. | 4.837506 | 0.789032 | 0.335636 |
| 35. | 1. | 0. | -0.320337 | 0.659046 | 1.437218 |
| 36. | 1. | 0. | 0.814232 | -1.690173 | -1.183370 |
| 37. | 1. | 0. | -4.423416 | -1.136169 | 2.138291 |
| 38. | 1. | 0. | -5.345836 | 0.116356 | 1.311571 |
| 39. | 1. | 0. | -5.921863 | -2.487197 | 0.718166 |
| 40. | 1. | 0. | -5.763974 | -1.338273 | -0.615536 |
| 41. | 1. | 0. | -3.772477 | -3.472825 | 0.224411 |
| 42. | 1. | 0. | -4.035587 | -2.784474 | -1.379066 |
| 43. | 1. | 0. | 4.997272 | -1.415556 | 1.789735 |
| 44. | 1. | 0. | 3.009885 | -2.247398 | -1.994054 |
| 45. | 1. | 0. | 4.564348 | -1.414844 | -2.063789 |
| 46. | 1. | 0. | 4.487476 | -3.110008 | -1.537441 |
| 47. | 1. | 0. | 6.521903 | -1.817274 | -0.851016 |
| 48. | 1. | 0. | 7.210168 | -1.578247 | 0.844462 |
| 49. | 1. | 0. | 2.757452 | -2.437823 | 1.761790 |
| 50. | 1. | 0. | 1.978211 | -2.930319 | 0.254614 |
| 51. | 1. | 0. | 3.491448 | -3.699913 | 0.755605 |
| 52. | 1. | 0. | -5.159530 | 1.761401 | -0.094783 |
| 53. | 1. | 0. | -3.541017 | 2.371900 | 0.272566 |
| 54. | 1. | 0. | -3.089284 | 2.643765 | -2.189252 |
| 55. | 1. | 0. | -4.534648 | 3.556514 | -1.706173 |
| 56. | 1. | 0. | -4.700533 | 2.005752 | -2.555974 |

| **4b-2** | | Standard Orientation (Ångstroms) | | | |
|---|---|---|---|---|---|
| Center number | Atom number | Type | X | Y | Z |
| 1. | 6. | 0. | -4.105989 | 2.778042 | 0.599653 |
| 2. | 6. | 0. | -3.449362 | 1.584867 | 0.286658 |
| 3. | 6. | 0. | -2.147325 | 1.550444 | -0.278641 |
| 4. | 6. | 0. | -1.516838 | 2.773273 | -0.580158 |

| | | | | | |
|---|---|---|---|---|---|
| 5. | 6. | 0. | -2.164369 | 3.963388 | -0.269576 |
| 6. | 6. | 0. | -3.442341 | 3.967625 | 0.322399 |
| 7. | 7. | 0. | -3.869764 | 0.279222 | 0.408474 |
| 8. | 6. | 0. | -2.899232 | -0.589090 | -0.048256 |
| 9. | 6. | 0. | -1.802697 | 0.154172 | -0.477729 |
| 10. | 6. | 0. | -3.177408 | -2.091698 | -0.046574 |
| 11. | 7. | 0. | 1.038804 | 0.941452 | 0.191577 |
| 12. | 6. | 0. | 0.693051 | 0.003105 | -0.796619 |
| 13. | 6. | 0. | -0.576528 | -0.363769 | -1.078291 |
| 14. | 6. | 0. | 2.303087 | 1.220453 | 0.633752 |
| 15. | 6. | 0. | 3.385599 | 0.270835 | 0.116734 |
| 16. | 7. | 0. | 3.067238 | -0.242650 | -1.194550 |
| 17. | 6. | 0. | 1.809033 | -0.582223 | -1.608625 |
| 18. | 6. | 0. | 4.766549 | 0.896593 | -0.109547 |
| 19. | 6. | 0. | 5.427136 | -0.112460 | -1.069051 |
| 20. | 6. | 0. | 4.267214 | -0.624420 | -1.951536 |
| 21. | 8. | 0. | 1.612481 | -1.262729 | -2.611806 |
| 22. | 8. | 0. | 2.541861 | 2.100349 | 1.451689 |
| 23. | 6. | 0. | -4.280205 | -2.385059 | 0.964357 |
| 24. | 6. | 0. | -1.932836 | -2.889294 | 0.419257 |
| 25. | 6. | 0. | -5.522144 | -2.803045 | 0.714429 |
| 26. | 6. | 0. | -3.566663 | -2.530379 | -1.476575 |
| 27. | 8. | 0. | 3.424030 | -0.870330 | 0.996360 |
| 28. | 6. | 0. | 3.877875 | -0.668210 | 2.342505 |
| 29. | 6. | 0. | 3.519553 | -1.918418 | 3.129171 |
| 30. | 1. | 0. | -5.101476 | 2.776076 | 1.034296 |
| 31. | 1. | 0. | -0.547536 | 2.792279 | -1.068183 |
| 32. | 1. | 0. | -1.680007 | 4.908592 | -0.494973 |
| 33. | 1. | 0. | -3.921880 | 4.913706 | 0.554030 |
| 34. | 1. | 0. | -4.758318 | -0.031190 | 0.771301 |
| 35. | 1. | 0. | 0.298651 | 1.479672 | 0.627858 |
| 36. | 1. | 0. | -0.647776 | -1.113871 | -1.860664 |
| 37. | 1. | 0. | 4.634291 | 1.873302 | -0.585220 |
| 38. | 1. | 0. | 5.317942 | 1.055363 | 0.817444 |
| 39. | 1. | 0. | 6.228390 | 0.335485 | -1.661217 |
| 40. | 1. | 0. | 5.854864 | -0.940620 | -0.497822 |
| 41. | 1. | 0. | 4.235890 | -0.144770 | -2.935530 |
| 42. | 1. | 0. | 4.295809 | -1.705350 | -2.114741 |
| 43. | 1. | 0. | -3.982199 | -2.234198 | 2.003007 |
| 44. | 1. | 0. | -1.086065 | -2.747706 | -0.253869 |
| 45. | 1. | 0. | -1.619505 | -2.578419 | 1.420535 |
| 46. | 1. | 0. | -2.172557 | -3.956746 | 0.450547 |

| | | | | | |
|---|---|---|---|---|---|
| 47. | 1. | 0. | -5.889751 | -2.980155 | -0.291437 |
| 48. | 1. | 0. | -6.217702 | -3.002215 | 1.524375 |
| 49. | 1. | 0. | -4.441526 | -1.983285 | -1.839605 |
| 50. | 1. | 0. | -2.744822 | -2.337754 | -2.170799 |
| 51. | 1. | 0. | -3.790218 | -3.601655 | -1.503321 |
| 52. | 1. | 0. | 4.966016 | -0.510165 | 2.355387 |
| 53. | 1. | 0. | 3.408399 | 0.220638 | 2.779190 |
| 54. | 1. | 0. | 3.876304 | -1.831567 | 4.160357 |
| 55. | 1. | 0. | 3.976939 | -2.803470 | 2.677438 |
| 56. | 1. | 0. | 2.435961 | -2.064850 | 3.146764 |

| **4b-3** | | Standard Orientation (Ångstroms) | | | |
|---|---|---|---|---|---|
| Center number | Atom number | Type | X | Y | Z |
| 1. | 6. | 0. | 3.308144 | 3.291775 | 0.200467 |
| 2. | 6. | 0. | 3.000406 | 1.936475 | 0.048572 |
| 3. | 6. | 0. | 1.698270 | 1.479003 | -0.277967 |
| 4. | 6. | 0. | 0.682620 | 2.430538 | -0.488044 |
| 5. | 6. | 0. | 0.981254 | 3.778704 | -0.334114 |
| 6. | 6. | 0. | 2.278577 | 4.206169 | 0.012361 |
| 7. | 7. | 0. | 3.806775 | 0.823693 | 0.136267 |
| 8. | 6. | 0. | 3.088555 | -0.324538 | -0.138187 |
| 9. | 6. | 0. | 1.763272 | 0.030833 | -0.383163 |
| 10. | 6. | 0. | 3.806721 | -1.664563 | 0.013708 |
| 11. | 7. | 0. | -0.966444 | 0.062426 | 0.875564 |
| 12. | 6. | 0. | -0.599719 | -0.796903 | -0.174777 |
| 13. | 6. | 0. | 0.645041 | -0.852619 | -0.697419 |
| 14. | 6. | 0. | -2.234550 | 0.262957 | 1.346676 |
| 15. | 6. | 0. | -3.330648 | -0.358046 | 0.476555 |
| 16. | 7. | 0. | -2.883575 | -1.586450 | -0.139538 |
| 17. | 6. | 0. | -1.625992 | -1.785479 | -0.638197 |
| 18. | 6. | 0. | -4.589368 | -0.811920 | 1.225196 |
| 19. | 6. | 0. | -5.204343 | -1.828627 | 0.245073 |
| 20. | 6. | 0. | -3.986746 | -2.520042 | -0.404253 |
| 21. | 8. | 0. | -1.351340 | -2.739689 | -1.361075 |
| 22. | 8. | 0. | -2.482466 | 0.948650 | 2.330201 |
| 23. | 6. | 0. | 5.239157 | -1.483622 | -0.477719 |
| 24. | 6. | 0. | 3.173594 | -2.778738 | -0.851752 |
| 25. | 6. | 0. | 6.358923 | -1.554222 | 0.244352 |
| 26. | 6. | 0. | 3.746226 | -2.089555 | 1.499495 |
| 27. | 8. | 0. | -3.613586 | 0.556454 | -0.600664 |

| | | | | | |
|---|---|---|---|---|---|
| 28. | 6. | 0. | -4.193013 | 1.825796 | -0.267749 |
| 29. | 6. | 0. | -4.170982 | 2.673852 | -1.528852 |
| 30. | 1. | 0. | 4.313512 | 3.617569 | 0.451236 |
| 31. | 1. | 0. | -0.315755 | 2.115964 | -0.773657 |
| 32. | 1. | 0. | 0.202524 | 4.519553 | -0.488238 |
| 33. | 1. | 0. | 2.479335 | 5.267128 | 0.126234 |
| 34. | 1. | 0. | 4.800448 | 0.819589 | 0.311249 |
| 35. | 1. | 0. | -0.233420 | 0.572958 | 1.354722 |
| 36. | 1. | 0. | 0.778950 | -1.650999 | -1.417719 |
| 37. | 1. | 0. | -4.284914 | -1.291315 | 2.160679 |
| 38. | 1. | 0. | -5.252319 | 0.014342 | 1.482684 |
| 39. | 1. | 0. | -5.867511 | -2.541558 | 0.740381 |
| 40. | 1. | 0. | -5.785646 | -1.302377 | -0.516921 |
| 41. | 1. | 0. | -3.755132 | -3.488247 | 0.052717 |
| 42. | 1. | 0. | -4.104650 | -2.684411 | -1.478857 |
| 43. | 1. | 0. | 5.318070 | -1.277273 | -1.545765 |
| 44. | 1. | 0. | 2.170137 | -3.036243 | -0.507561 |
| 45. | 1. | 0. | 3.112942 | -2.484537 | -1.903940 |
| 46. | 1. | 0. | 3.791726 | -3.679086 | -0.786848 |
| 47. | 1. | 0. | 6.356917 | -1.761161 | 1.309992 |
| 48. | 1. | 0. | 7.331857 | -1.424827 | -0.220373 |
| 49. | 1. | 0. | 4.194689 | -1.337134 | 2.154749 |
| 50. | 1. | 0. | 2.704979 | -2.220420 | 1.805250 |
| 51. | 1. | 0. | 4.270608 | -3.038493 | 1.651597 |
| 52. | 1. | 0. | -5.226903 | 1.693072 | 0.081695 |
| 53. | 1. | 0. | -3.632159 | 2.307739 | 0.541856 |
| 54. | 1. | 0. | -3.144253 | 2.835397 | -1.869427 |
| 55. | 1. | 0. | -4.630937 | 3.648279 | -1.336543 |
| 56. | 1. | 0. | -4.723650 | 2.182094 | -2.334520 |

| **4b-4** | | Standard Orientation (Ångstroms) | | | |
|---|---|---|---|---|---|
| Center number | Atom number | Type | X | Y | Z |
| 1. | 6. | 0. | -4.093065 | 2.688700 | 0.747880 |
| 2. | 6. | 0. | -3.429973 | 1.534025 | 0.322639 |
| 3. | 6. | 0. | -2.154945 | 1.569401 | -0.299679 |
| 4. | 6. | 0. | -1.550135 | 2.820946 | -0.524099 |
| 5. | 6. | 0. | -2.201623 | 3.971921 | -0.096861 |
| 6. | 6. | 0. | -3.457624 | 3.907220 | 0.537897 |
| 7. | 7. | 0. | -3.818431 | 0.214391 | 0.364792 |
| 8. | 6. | 0. | -2.860069 | -0.595859 | -0.215933 |

| | | | | | |
|---|---|---|---|---|---|
| 9. | 6. | 0. | -1.795443 | 0.201256 | -0.626395 |
| 10. | 6. | 0. | -3.076726 | -2.106914 | -0.166873 |
| 11. | 7. | 0. | 0.991660 | 0.942692 | 0.227134 |
| 12. | 6. | 0. | 0.704085 | 0.123341 | -0.879033 |
| 13. | 6. | 0. | -0.545491 | -0.224699 | -1.256362 |
| 14. | 6. | 0. | 2.229518 | 1.163000 | 0.766210 |
| 15. | 6. | 0. | 3.336099 | 0.269086 | 0.202122 |
| 16. | 7. | 0. | 3.096954 | -0.065884 | -1.181937 |
| 17. | 6. | 0. | 1.864779 | -0.347481 | -1.704095 |
| 18. | 6. | 0. | 4.734313 | 0.894710 | 0.144904 |
| 19. | 6. | 0. | 5.444533 | 0.005239 | -0.893504 |
| 20. | 6. | 0. | 4.339656 | -0.359553 | -1.909422 |
| 21. | 8. | 0. | 1.725151 | -0.892313 | -2.795371 |
| 22. | 8. | 0. | 2.424730 | 1.944794 | 1.688441 |
| 23. | 6. | 0. | -4.567560 | -2.372487 | -0.347117 |
| 24. | 6. | 0. | -2.544708 | -2.636948 | 1.184770 |
| 25. | 6. | 0. | -5.412006 | -2.892243 | 0.545613 |
| 26. | 6. | 0. | -2.347712 | -2.841003 | -1.315863 |
| 27. | 8. | 0. | 3.307463 | -0.975865 | 0.928147 |
| 28. | 6. | 0. | 3.667675 | -0.952752 | 2.316729 |
| 29. | 6. | 0. | 3.226392 | -2.277231 | 2.917831 |
| 30. | 1. | 0. | -5.069505 | 2.636421 | 1.220798 |
| 31. | 1. | 0. | -0.597274 | 2.889943 | -1.039709 |
| 32. | 1. | 0. | -1.738994 | 4.940201 | -0.261805 |
| 33. | 1. | 0. | -3.941555 | 4.824127 | 0.860153 |
| 34. | 1. | 0. | -4.697727 | -0.138397 | 0.711467 |
| 35. | 1. | 0. | 0.227965 | 1.439611 | 0.672132 |
| 36. | 1. | 0. | -0.575902 | -0.862853 | -2.132663 |
| 37. | 1. | 0. | 4.641471 | 1.926268 | -0.208534 |
| 38. | 1. | 0. | 5.226899 | 0.923442 | 1.117146 |
| 39. | 1. | 0. | 6.291042 | 0.506983 | -1.367869 |
| 40. | 1. | 0. | 5.818907 | -0.900646 | -0.409375 |
| 41. | 1. | 0. | 4.376440 | 0.253086 | -2.816537 |
| 42. | 1. | 0. | 4.368446 | -1.407633 | -2.219955 |
| 43. | 1. | 0. | -4.950999 | -2.097064 | -1.330337 |
| 44. | 1. | 0. | -1.472920 | -2.437353 | 1.263578 |
| 45. | 1. | 0. | -3.038200 | -2.152352 | 2.032289 |
| 46. | 1. | 0. | -2.700380 | -3.717744 | 1.264620 |
| 47. | 1. | 0. | -5.100009 | -3.194213 | 1.540578 |
| 48. | 1. | 0. | -6.456950 | -3.053419 | 0.297690 |
| 49. | 1. | 0. | -2.635999 | -2.441685 | -2.293023 |
| 50. | 1. | 0. | -1.263539 | -2.761645 | -1.217510 |

| | | | | | |
|---|---|---|---|---|---|
| 51. | 1. | 0. | -2.611383 | -3.902562 | -1.291094 |
| 52. | 1. | 0. | 4.755312 | -0.831306 | 2.422917 |
| 53. | 1. | 0. | 3.188254 | -0.109439 | 2.826936 |
| 54. | 1. | 0. | 3.513289 | -2.328391 | 3.972930 |
| 55. | 1. | 0. | 3.692558 | -3.114780 | 2.390782 |
| 56. | 1. | 0. | 2.140913 | -2.389955 | 2.846574 |

| **4b-5** | | Standard Orientation (Ångstroms) | | | |
|---|---|---|---|---|---|
| Center number | Atom number | Type | X | Y | Z |
| 1. | 6. | 0. | -4.387187 | 2.587074 | 0.574551 |
| 2. | 6. | 0. | -3.668100 | 1.420344 | 0.303546 |
| 3. | 6. | 0. | -2.385633 | 1.431435 | -0.304826 |
| 4. | 6. | 0. | -1.845037 | 2.671035 | -0.699030 |
| 5. | 6. | 0. | -2.554822 | 3.835882 | -0.431281 |
| 6. | 6. | 0. | -3.809091 | 3.797101 | 0.207802 |
| 7. | 7. | 0. | -4.007790 | 0.099976 | 0.508242 |
| 8. | 6. | 0. | -2.999837 | -0.734115 | 0.063380 |
| 9. | 6. | 0. | -1.962606 | 0.049546 | -0.435099 |
| 10. | 6. | 0. | -3.141339 | -2.251527 | 0.138558 |
| 11. | 7. | 0. | 0.845282 | 1.074872 | 0.083104 |
| 12. | 6. | 0. | 0.533913 | 0.018658 | -0.792874 |
| 13. | 6. | 0. | -0.715875 | -0.447919 | -1.006819 |
| 14. | 6. | 0. | 2.098434 | 1.455004 | 0.477413 |
| 15. | 6. | 0. | 3.218514 | 0.504574 | 0.049810 |
| 16. | 7. | 0. | 2.913479 | -0.153887 | -1.197865 |
| 17. | 6. | 0. | 1.668202 | -0.592162 | -1.557896 |
| 18. | 6. | 0. | 4.569711 | 1.162198 | -0.254534 |
| 19. | 6. | 0. | 5.265767 | 0.089572 | -1.114800 |
| 20. | 6. | 0. | 4.122611 | -0.554596 | -1.929567 |
| 21. | 8. | 0. | 1.497345 | -1.378136 | -2.485808 |
| 22. | 8. | 0. | 2.303691 | 2.426804 | 1.194836 |
| 23. | 6. | 0. | -1.861106 | -2.844725 | 0.714478 |
| 24. | 6. | 0. | -3.472697 | -2.798510 | -1.269063 |
| 25. | 6. | 0. | -1.136728 | -3.841086 | 0.208025 |
| 26. | 6. | 0. | -4.290148 | -2.651686 | 1.100802 |
| 27. | 8. | 0. | 3.314437 | -0.536975 | 1.042128 |
| 28. | 6. | 0. | 3.746770 | -0.174355 | 2.360711 |
| 29. | 6. | 0. | 3.482987 | -1.368792 | 3.262990 |
| 30. | 1. | 0. | -5.365799 | 2.550168 | 1.044799 |
| 31. | 1. | 0. | -0.898063 | 2.720931 | -1.227009 |

| | | | | | |
|---|---|---|---|---|---|
| 32. | 1. | 0. | -2.139268 | 4.793750 | -0.728820 |
| 33. | 1. | 0. | -4.338950 | 4.724010 | 0.404512 |
| 34. | 1. | 0. | -4.852077 | -0.214016 | 0.956663 |
| 35. | 1. | 0. | 0.086057 | 1.618600 | 0.477315 |
| 36. | 1. | 0. | -0.751372 | -1.293398 | -1.685042 |
| 37. | 1. | 0. | 4.391974 | 2.078876 | -0.825345 |
| 38. | 1. | 0. | 5.120741 | 1.437086 | 0.645122 |
| 39. | 1. | 0. | 6.044810 | 0.507452 | -1.756595 |
| 40. | 1. | 0. | 5.730139 | -0.659100 | -0.467428 |
| 41. | 1. | 0. | 4.062792 | -0.173750 | -2.954728 |
| 42. | 1. | 0. | 4.197468 | -1.643986 | -1.988928 |
| 43. | 1. | 0. | -1.556292 | -2.401398 | 1.662231 |
| 44. | 1. | 0. | -4.391068 | -2.337936 | -1.644810 |
| 45. | 1. | 0. | -2.674112 | -2.587148 | -1.983926 |
| 46. | 1. | 0. | -3.625921 | -3.881961 | -1.237103 |
| 47. | 1. | 0. | -1.372237 | -4.319320 | -0.737184 |
| 48. | 1. | 0. | -0.253220 | -4.203550 | 0.723802 |
| 49. | 1. | 0. | -4.128935 | -2.262284 | 2.111418 |
| 50. | 1. | 0. | -5.264692 | -2.302760 | 0.738174 |
| 51. | 1. | 0. | -4.342057 | -3.741012 | 1.168364 |
| 52. | 1. | 0. | 4.819279 | 0.068110 | 2.355616 |
| 53. | 1. | 0. | 3.209379 | 0.713029 | 2.714479 |
| 54. | 1. | 0. | 2.413914 | -1.596632 | 3.297000 |
| 55. | 1. | 0. | 3.826796 | -1.157501 | 4.280499 |
| 56. | 1. | 0. | 4.009922 | -2.254242 | 2.895658 |

| **4b-6** | | Standard Orientation (Ångstroms) | | | |
|---|---|---|---|---|---|
| Center number | Atom number | Type | X | Y | Z |
| 1. | 6. | 0. | 3.547886 | 3.334506 | 0.268222 |
| 2. | 6. | 0. | 3.195144 | 1.989576 | 0.129369 |
| 3. | 6. | 0. | 1.867050 | 1.564595 | -0.131999 |
| 4. | 6. | 0. | 0.874874 | 2.547795 | -0.310401 |
| 5. | 6. | 0. | 1.218711 | 3.887401 | -0.175096 |
| 6. | 6. | 0. | 2.539035 | 4.279079 | 0.120695 |
| 7. | 7. | 0. | 3.982448 | 0.857776 | 0.174493 |
| 8. | 6. | 0. | 3.220561 | -0.272383 | -0.044259 |
| 9. | 6. | 0. | 1.893478 | 0.114930 | -0.224873 |
| 10. | 6. | 0. | 3.860690 | -1.657458 | -0.085120 |
| 11. | 7. | 0. | -0.907824 | 0.240757 | 0.924586 |
| 12. | 6. | 0. | -0.487952 | -0.693263 | -0.040990 |

| | | | | | |
|---|---|---|---|---|---|
| 13. | 6. | 0. | 0.779908 | -0.781092 | -0.500908 |
| 14. | 6. | 0. | -2.197729 | 0.466943 | 1.318018 |
| 15. | 6. | 0. | -3.244629 | -0.220561 | 0.436875 |
| 16. | 7. | 0. | -2.766211 | -1.497667 | -0.039952 |
| 17. | 6. | 0. | -1.486239 | -1.723621 | -0.470242 |
| 18. | 6. | 0. | -4.550622 | -0.605247 | 1.141831 |
| 19. | 6. | 0. | -5.107216 | -1.707294 | 0.220378 |
| 20. | 6. | 0. | -3.854763 | -2.452149 | -0.288290 |
| 21. | 8. | 0. | -1.179747 | -2.725741 | -1.110628 |
| 22. | 8. | 0. | -2.495842 | 1.213001 | 2.242007 |
| 23. | 6. | 0. | 3.041988 | -2.613828 | 0.774206 |
| 24. | 6. | 0. | 5.287164 | -1.623891 | 0.522979 |
| 25. | 6. | 0. | 2.558384 | -3.802557 | 0.417948 |
| 26. | 6. | 0. | 3.971104 | -2.120668 | -1.556093 |
| 27. | 8. | 0. | -3.458113 | 0.594846 | -0.731960 |
| 28. | 6. | 0. | -3.998052 | 1.909213 | -0.542570 |
| 29. | 6. | 0. | -3.957003 | 2.608842 | -1.891191 |
| 30. | 1. | 0. | 4.572636 | 3.630981 | 0.473501 |
| 31. | 1. | 0. | -0.141587 | 2.266105 | -0.562324 |
| 32. | 1. | 0. | 0.456212 | 4.649364 | -0.305515 |
| 33. | 1. | 0. | 2.774893 | 5.333919 | 0.222779 |
| 34. | 1. | 0. | 4.968436 | 0.851261 | 0.375236 |
| 35. | 1. | 0. | -0.199420 | 0.766582 | 1.423389 |
| 36. | 1. | 0. | 0.953370 | -1.654145 | -1.119563 |
| 37. | 1. | 0. | -4.310276 | -0.996149 | 2.135187 |
| 38. | 1. | 0. | -5.223993 | 0.241514 | 1.276824 |
| 39. | 1. | 0. | -5.803143 | -2.373755 | 0.735174 |
| 40. | 1. | 0. | -5.637438 | -1.252083 | -0.620479 |
| 41. | 1. | 0. | -3.656776 | -3.376357 | 0.265686 |
| 42. | 1. | 0. | -3.906638 | -2.711184 | -1.349429 |
| 43. | 1. | 0. | 2.884962 | -2.265842 | 1.795151 |
| 44. | 1. | 0. | 5.964901 | -0.991671 | -0.063471 |
| 45. | 1. | 0. | 5.279769 | -1.268734 | 1.558732 |
| 46. | 1. | 0. | 5.704563 | -2.633687 | 0.520621 |
| 47. | 1. | 0. | 2.663223 | -4.204913 | -0.584342 |
| 48. | 1. | 0. | 2.010307 | -4.415228 | 1.126544 |
| 49. | 1. | 0. | 2.990596 | -2.204422 | -2.030151 |
| 50. | 1. | 0. | 4.562584 | -1.403112 | -2.132231 |
| 51. | 1. | 0. | 4.466167 | -3.095041 | -1.617759 |
| 52. | 1. | 0. | -5.032743 | 1.849768 | -0.176052 |
| 53. | 1. | 0. | -3.418853 | 2.463306 | 0.206261 |
| 54. | 1. | 0. | -4.379472 | 3.615197 | -1.808238 |

| | | | | | |
|---|---|---|---|---|---|
| 55. | 1. | 0. | -4.534200 | 2.050058 | -2.633451 |
| 56. | 1. | 0. | -2.927973 | 2.692146 | -2.252264 |

| **4b-7** | | Standard Orientation (Ångstroms) | | | |
|---|---|---|---|---|---|
| Center number | Atom number | Type | X | Y | Z |
| 1. | 6. | 0. | -3.552046 | 3.339143 | -0.187012 |
| 2. | 6. | 0. | -3.192222 | 1.990525 | -0.121661 |
| 3. | 6. | 0. | -1.867018 | 1.559223 | 0.144612 |
| 4. | 6. | 0. | -0.885691 | 2.536221 | 0.400561 |
| 5. | 6. | 0. | -1.236481 | 3.879268 | 0.337208 |
| 6. | 6. | 0. | -2.553603 | 4.279475 | 0.038171 |
| 7. | 7. | 0. | -3.969563 | 0.858399 | -0.253835 |
| 8. | 6. | 0. | -3.204994 | -0.277614 | -0.082258 |
| 9. | 6. | 0. | -1.884967 | 0.107291 | 0.153753 |
| 10. | 6. | 0. | -3.819670 | -1.672572 | -0.173324 |
| 11. | 7. | 0. | 0.933238 | 0.301621 | -0.941505 |
| 12. | 6. | 0. | 0.497207 | -0.694467 | -0.047440 |
| 13. | 6. | 0. | -0.776244 | -0.803967 | 0.393454 |
| 14. | 6. | 0. | 2.229454 | 0.548112 | -1.301623 |
| 15. | 6. | 0. | 3.261838 | -0.203265 | -0.455359 |
| 16. | 7. | 0. | 2.772040 | -1.509083 | -0.076762 |
| 17. | 6. | 0. | 1.484750 | -1.761234 | 0.313948 |
| 18. | 6. | 0. | 4.576777 | -0.542857 | -1.167588 |
| 19. | 6. | 0. | 5.113955 | -1.713081 | -0.321951 |
| 20. | 6. | 0. | 3.850765 | -2.489462 | 0.104024 |
| 21. | 8. | 0. | 1.162582 | -2.811510 | 0.863107 |
| 22. | 8. | 0. | 2.542955 | 1.353993 | -2.168103 |
| 23. | 6. | 0. | -3.676970 | -2.335657 | 1.193604 |
| 24. | 6. | 0. | -3.136353 | -2.474584 | -1.301616 |
| 25. | 6. | 0. | -3.064871 | -3.485808 | 1.474855 |
| 26. | 6. | 0. | -5.334770 | -1.593148 | -0.484675 |
| 27. | 8. | 0. | 3.464308 | 0.525699 | 0.770690 |
| 28. | 6. | 0. | 3.973834 | 1.862139 | 0.679296 |
| 29. | 6. | 0. | 3.996456 | 2.429763 | 2.088965 |
| 30. | 1. | 0. | -4.574339 | 3.641065 | -0.396521 |
| 31. | 1. | 0. | 0.127611 | 2.246107 | 0.655961 |
| 32. | 1. | 0. | -0.482365 | 4.636972 | 0.527458 |
| 33. | 1. | 0. | -2.795239 | 5.337020 | -0.005825 |
| 34. | 1. | 0. | -4.954097 | 0.857755 | -0.461881 |
| 35. | 1. | 0. | 0.233642 | 0.866080 | -1.409730 |

| | | | | | |
|---|---|---|---|---|---|
| 36. | 1. | 0. | -0.971423 | -1.706304 | 0.964371 |
| 37. | 1. | 0. | 4.350535 | -0.857195 | -2.191042 |
| 38. | 1. | 0. | 5.256127 | 0.307723 | -1.228890 |
| 39. | 1. | 0. | 5.818961 | -2.341063 | -0.871595 |
| 40. | 1. | 0. | 5.627371 | -1.323516 | 0.561173 |
| 41. | 1. | 0. | 3.655736 | -3.361345 | -0.530186 |
| 42. | 1. | 0. | 3.885405 | -2.838729 | 1.139606 |
| 43. | 1. | 0. | -4.152378 | -1.780947 | 2.003102 |
| 44. | 1. | 0. | -3.277008 | -1.966615 | -2.260424 |
| 45. | 1. | 0. | -2.063124 | -2.580641 | -1.132035 |
| 46. | 1. | 0. | -3.575482 | -3.474231 | -1.379097 |
| 47. | 1. | 0. | -2.558211 | -4.082396 | 0.723411 |
| 48. | 1. | 0. | -3.038497 | -3.867558 | 2.490764 |
| 49. | 1. | 0. | -5.888239 | -1.061409 | 0.297475 |
| 50. | 1. | 0. | -5.522120 | -1.103917 | -1.447652 |
| 51. | 1. | 0. | -5.746375 | -2.603805 | -0.541980 |
| 52. | 1. | 0. | 4.986548 | 1.860046 | 0.252512 |
| 53. | 1. | 0. | 3.345552 | 2.473461 | 0.019532 |
| 54. | 1. | 0. | 2.988424 | 2.452040 | 2.512704 |
| 55. | 1. | 0. | 4.392551 | 3.450069 | 2.079027 |
| 56. | 1. | 0. | 4.626850 | 1.818671 | 2.741265 |

| **4b-8** | | Standard Orientation (Ångstroms) | | | |
|---|---|---|---|---|---|
| Center number | Atom number | Type | X | Y | Z |
| 1. | 6. | 0. | -4.323363 | 2.682265 | 0.435678 |
| 2. | 6. | 0. | -3.616388 | 1.489834 | 0.262062 |
| 3. | 6. | 0. | -2.347890 | 1.433904 | -0.372762 |
| 4. | 6. | 0. | -1.808167 | 2.626788 | -0.892471 |
| 5. | 6. | 0. | -2.505388 | 3.816999 | -0.720810 |
| 6. | 6. | 0. | -3.746467 | 3.847778 | -0.055857 |
| 7. | 7. | 0. | -3.957884 | 0.197323 | 0.599817 |
| 8. | 6. | 0. | -2.966758 | -0.682825 | 0.210277 |
| 9. | 6. | 0. | -1.936654 | 0.043106 | -0.383852 |
| 10. | 6. | 0. | -3.087226 | -2.183030 | 0.465669 |
| 11. | 7. | 0. | 0.884025 | 1.077393 | 0.008429 |
| 12. | 6. | 0. | 0.553710 | -0.051590 | -0.763851 |
| 13. | 6. | 0. | -0.704561 | -0.518763 | -0.925689 |
| 14. | 6. | 0. | 2.144443 | 1.470403 | 0.366323 |
| 15. | 6. | 0. | 3.244903 | 0.462053 | 0.030113 |
| 16. | 7. | 0. | 2.928920 | -0.294586 | -1.157705 |

| | | | | | |
|---|---|---|---|---|---|
| 17. | 6. | 0. | 1.676051 | -0.744146 | -1.476021 |
| 18. | 6. | 0. | 4.612081 | 1.059588 | -0.322587 |
| 19. | 6. | 0. | 5.285666 | -0.099242 | -1.082542 |
| 20. | 6. | 0. | 4.130918 | -0.783084 | -1.846863 |
| 21. | 8. | 0. | 1.490699 | -1.608011 | -2.328499 |
| 22. | 8. | 0. | 2.368511 | 2.501248 | 0.989017 |
| 23. | 6. | 0. | -3.093024 | -2.887801 | -0.887625 |
| 24. | 6. | 0. | -4.428225 | -2.523484 | 1.161262 |
| 25. | 6. | 0. | -2.250826 | -3.829571 | -1.312127 |
| 26. | 6. | 0. | -1.933903 | -2.650684 | 1.378539 |
| 27. | 8. | 0. | 3.312477 | -0.492009 | 1.109257 |
| 28. | 6. | 0. | 3.736603 | -0.024468 | 2.397133 |
| 29. | 6. | 0. | 3.467470 | -1.142990 | 3.390471 |
| 30. | 1. | 0. | -5.291768 | 2.697656 | 0.927761 |
| 31. | 1. | 0. | -0.872013 | 2.618776 | -1.441771 |
| 32. | 1. | 0. | -2.090623 | 4.739701 | -1.114923 |
| 33. | 1. | 0. | -4.266924 | 4.793073 | 0.063082 |
| 34. | 1. | 0. | -4.802025 | -0.068941 | 1.078371 |
| 35. | 1. | 0. | 0.134473 | 1.667863 | 0.350584 |
| 36. | 1. | 0. | -0.770055 | -1.421516 | -1.525378 |
| 37. | 1. | 0. | 4.457148 | 1.927204 | -0.971392 |
| 38. | 1. | 0. | 5.166314 | 1.399164 | 0.552663 |
| 39. | 1. | 0. | 6.077949 | 0.242407 | -1.752626 |
| 40. | 1. | 0. | 5.727777 | -0.800464 | -0.369752 |
| 41. | 1. | 0. | 4.084234 | -0.488040 | -2.900594 |
| 42. | 1. | 0. | 4.180054 | -1.875057 | -1.814359 |
| 43. | 1. | 0. | -3.898267 | -2.565565 | -1.548403 |
| 44. | 1. | 0. | -4.507637 | -2.036768 | 2.140337 |
| 45. | 1. | 0. | -5.291969 | -2.236939 | 0.550918 |
| 46. | 1. | 0. | -4.488926 | -3.602439 | 1.323313 |
| 47. | 1. | 0. | -1.419963 | -4.188351 | -0.713249 |
| 48. | 1. | 0. | -2.361084 | -4.271956 | -2.297290 |
| 49. | 1. | 0. | -0.958221 | -2.447137 | 0.932936 |
| 50. | 1. | 0. | -1.980307 | -2.127566 | 2.338339 |
| 51. | 1. | 0. | -2.010807 | -3.725210 | 1.572773 |
| 52. | 1. | 0. | 4.808882 | 0.218504 | 2.379731 |
| 53. | 1. | 0. | 3.196802 | 0.888008 | 2.675517 |
| 54. | 1. | 0. | 2.398534 | -1.369780 | 3.434481 |
| 55. | 1. | 0. | 3.802795 | -0.849612 | 4.390294 |
| 56. | 1. | 0. | 3.998679 | -2.053989 | 3.099823 |

| **4b-9** | Standard Orientation |
|---|---|

| | | (Ångstroms) | | | |
|---|---|---|---|---|---|
| Center number | Atom number | Type | X | Y | Z |
| 1. | 6. | 0. | 3.374659 | 3.271324 | 0.315809 |
| 2. | 6. | 0. | 3.030243 | 1.925023 | 0.166425 |
| 3. | 6. | 0. | 1.705431 | 1.496901 | -0.108147 |
| 4. | 6. | 0. | 0.708440 | 2.474856 | -0.285111 |
| 5. | 6. | 0. | 1.044730 | 3.815172 | -0.138940 |
| 6. | 6. | 0. | 2.361761 | 4.211233 | 0.166847 |
| 7. | 7. | 0. | 3.817971 | 0.795190 | 0.214793 |
| 8. | 6. | 0. | 3.063130 | -0.335015 | -0.016243 |
| 9. | 6. | 0. | 1.737254 | 0.048308 | -0.215058 |
| 10. | 6. | 0. | 3.749514 | -1.700389 | -0.056962 |
| 11. | 7. | 0. | -1.069406 | 0.114520 | 0.920791 |
| 12. | 6. | 0. | -0.644399 | -0.768557 | -0.088660 |
| 13. | 6. | 0. | 0.625764 | -0.832336 | -0.545146 |
| 14. | 6. | 0. | -2.365480 | 0.334626 | 1.300318 |
| 15. | 6. | 0. | -3.394053 | -0.280122 | 0.336467 |
| 16. | 7. | 0. | -2.920705 | -1.558741 | -0.147283 |
| 17. | 6. | 0. | -1.636689 | -1.785035 | -0.564219 |
| 18. | 6. | 0. | -4.771964 | -0.601457 | 0.966605 |
| 19. | 6. | 0. | -4.937306 | -2.132991 | 0.839545 |
| 20. | 6. | 0. | -4.013052 | -2.511930 | -0.326196 |
| 21. | 8. | 0. | -1.314539 | -2.777357 | -1.212040 |
| 22. | 8. | 0. | -2.672217 | 1.020790 | 2.265536 |
| 23. | 6. | 0. | 5.071812 | -1.606537 | 0.696339 |
| 24. | 6. | 0. | 3.949267 | -2.115003 | -1.532633 |
| 25. | 6. | 0. | 6.303170 | -1.678168 | 0.188226 |
| 26. | 6. | 0. | 2.906159 | -2.776376 | 0.674868 |
| 27. | 8. | 0. | -3.492293 | 0.582454 | -0.810573 |
| 28. | 6. | 0. | -3.917640 | 1.930549 | -0.579277 |
| 29. | 6. | 0. | -4.041592 | 2.601233 | -1.937105 |
| 30. | 1. | 0. | 4.396344 | 3.571640 | 0.529924 |
| 31. | 1. | 0. | -0.304715 | 2.187462 | -0.544525 |
| 32. | 1. | 0. | 0.279369 | 4.574545 | -0.268126 |
| 33. | 1. | 0. | 2.591555 | 5.266688 | 0.276480 |
| 34. | 1. | 0. | 4.812083 | 0.766892 | 0.384066 |
| 35. | 1. | 0. | -0.362838 | 0.603298 | 1.458513 |
| 36. | 1. | 0. | 0.797111 | -1.660275 | -1.225876 |
| 37. | 1. | 0. | -4.825026 | -0.241180 | 1.995133 |
| 38. | 1. | 0. | -5.539728 | -0.084223 | 0.385488 |
| 39. | 1. | 0. | -4.594486 | -2.630142 | 1.752594 |

| | | | | | |
|---|---|---|---|---|---|
| 40. | 1. | 0. | -5.975348 | -2.428649 | 0.666607 |
| 41. | 1. | 0. | -3.619866 | -3.528827 | -0.288291 |
| 42. | 1. | 0. | -4.500057 | -2.368172 | -1.299763 |
| 43. | 1. | 0. | 4.961223 | -1.475900 | 1.773873 |
| 44. | 1. | 0. | 2.983813 | -2.203711 | -2.036671 |
| 45. | 1. | 0. | 4.541725 | -1.375961 | -2.079752 |
| 46. | 1. | 0. | 4.456117 | -3.083243 | -1.595748 |
| 47. | 1. | 0. | 6.492508 | -1.813521 | -0.872003 |
| 48. | 1. | 0. | 7.176672 | -1.621710 | 0.831104 |
| 49. | 1. | 0. | 2.718780 | -2.487216 | 1.713553 |
| 50. | 1. | 0. | 1.940984 | -2.937912 | 0.192570 |
| 51. | 1. | 0. | 3.449939 | -3.726216 | 0.677292 |
| 52. | 1. | 0. | -4.881372 | 1.945993 | -0.051047 |
| 53. | 1. | 0. | -3.195046 | 2.461470 | 0.054595 |
| 54. | 1. | 0. | -4.356448 | 3.642628 | -1.817437 |
| 55. | 1. | 0. | -4.778418 | 2.083920 | -2.558127 |
| 56. | 1. | 0. | -3.082441 | 2.586582 | -2.462442 |

| **4b-10** | | Standard Orientation (Ångstroms) | | | |
|---|---|---|---|---|---|
| Center number | Atom number | Type | X | Y | Z |
| 1. | 6. | 0. | -3.572590 | 3.225146 | -0.269759 |
| 2. | 6. | 0. | -3.197472 | 1.881062 | -0.193709 |
| 3. | 6. | 0. | -1.869304 | 1.466793 | 0.084225 |
| 4. | 6. | 0. | -0.900951 | 2.456773 | 0.340596 |
| 5. | 6. | 0. | -1.266926 | 3.795110 | 0.266056 |
| 6. | 6. | 0. | -2.586666 | 4.178232 | -0.044253 |
| 7. | 7. | 0. | -3.960148 | 0.739560 | -0.323122 |
| 8. | 6. | 0. | -3.185391 | -0.387256 | -0.137396 |
| 9. | 6. | 0. | -1.870896 | 0.014622 | 0.102190 |
| 10. | 6. | 0. | -3.785283 | -1.780897 | -0.251674 |
| 11. | 7. | 0. | 0.954825 | 0.238814 | -0.964238 |
| 12. | 6. | 0. | 0.525606 | -0.751871 | -0.061818 |
| 13. | 6. | 0. | -0.751359 | -0.878466 | 0.364112 |
| 14. | 6. | 0. | 2.251226 | 0.510053 | -1.305456 |
| 15. | 6. | 0. | 3.287136 | -0.210490 | -0.438050 |
| 16. | 7. | 0. | 2.817536 | -1.517674 | -0.040657 |
| 17. | 6. | 0. | 1.529755 | -1.790855 | 0.333721 |
| 18. | 6. | 0. | 4.614000 | -0.539780 | -1.131936 |
| 19. | 6. | 0. | 5.166562 | -1.679639 | -0.255410 |
| 20. | 6. | 0. | 3.914365 | -2.469733 | 0.179621 |

| | | | | | |
|---|---|---|---|---|---|
| 21. | 8. | 0. | 1.221301 | -2.836940 | 0.897978 |
| 22. | 8. | 0. | 2.560323 | 1.316527 | -2.173492 |
| 23. | 6. | 0. | -3.741547 | -2.538731 | 1.073090 |
| 24. | 6. | 0. | -3.015748 | -2.595400 | -1.325653 |
| 25. | 6. | 0. | -3.485159 | -2.049513 | 2.285007 |
| 26. | 6. | 0. | -5.272684 | -1.719226 | -0.683940 |
| 27. | 8. | 0. | 3.458423 | 0.546652 | 0.776213 |
| 28. | 6. | 0. | 4.024082 | 1.860353 | 0.671092 |
| 29. | 6. | 0. | 3.862895 | 2.529051 | 2.026463 |
| 30. | 1. | 0. | -4.596959 | 3.513688 | -0.487750 |
| 31. | 1. | 0. | 0.114027 | 2.180295 | 0.604442 |
| 32. | 1. | 0. | -0.522912 | 4.562741 | 0.456269 |
| 33. | 1. | 0. | -2.840063 | 5.232634 | -0.096627 |
| 34. | 1. | 0. | -4.945024 | 0.726092 | -0.528879 |
| 35. | 1. | 0. | 0.251554 | 0.787173 | -1.445860 |
| 36. | 1. | 0. | -0.936604 | -1.774599 | 0.947987 |
| 37. | 1. | 0. | 4.400329 | -0.883742 | -2.148530 |
| 38. | 1. | 0. | 5.276688 | 0.322271 | -1.210637 |
| 39. | 1. | 0. | 5.886702 | -2.306902 | -0.785896 |
| 40. | 1. | 0. | 5.667282 | -1.261211 | 0.621935 |
| 41. | 1. | 0. | 3.743604 | -3.363310 | -0.430695 |
| 42. | 1. | 0. | 3.945252 | -2.788252 | 1.225228 |
| 43. | 1. | 0. | -3.991949 | -3.595775 | 0.973874 |
| 44. | 1. | 0. | -3.082099 | -2.102402 | -2.300657 |
| 45. | 1. | 0. | -1.960670 | -2.704683 | -1.069137 |
| 46. | 1. | 0. | -3.450112 | -3.596613 | -1.419480 |
| 47. | 1. | 0. | -3.222914 | -1.008158 | 2.445256 |
| 48. | 1. | 0. | -3.516508 | -2.688992 | 3.161701 |
| 49. | 1. | 0. | -5.886290 | -1.183382 | 0.047849 |
| 50. | 1. | 0. | -5.385358 | -1.249312 | -1.667821 |
| 51. | 1. | 0. | -5.675895 | -2.732963 | -0.758428 |
| 52. | 1. | 0. | 5.088491 | 1.796660 | 0.403832 |
| 53. | 1. | 0. | 3.522441 | 2.437189 | -0.115308 |
| 54. | 1. | 0. | 2.805218 | 2.621679 | 2.289050 |
| 55. | 1. | 0. | 4.306476 | 3.529570 | 2.009391 |
| 56. | 1. | 0. | 4.357532 | 1.943469 | 2.806720 |

| **4b-11** | | Standard Orientation (Ångstroms) | | | |
|---|---|---|---|---|---|
| Center number | Atom number | Type | X | Y | Z |
| 1. | 6. | 0. | 3.391682 | 3.286367 | 0.208648 |

| | | | | | |
|---|---|---|---|---|---|
| 2. | 6. | 0. | 3.050543 | 1.935472 | 0.098451 |
| 3. | 6. | 0. | 1.725175 | 1.495795 | -0.154828 |
| 4. | 6. | 0. | 0.723683 | 2.465876 | -0.350024 |
| 5. | 6. | 0. | 1.056657 | 3.810668 | -0.242337 |
| 6. | 6. | 0. | 2.374699 | 4.218892 | 0.042160 |
| 7. | 7. | 0. | 3.842270 | 0.809927 | 0.172031 |
| 8. | 6. | 0. | 3.089444 | -0.328620 | -0.022080 |
| 9. | 6. | 0. | 1.761043 | 0.044745 | -0.222124 |
| 10. | 6. | 0. | 3.780747 | -1.692043 | -0.030463 |
| 11. | 7. | 0. | -1.030376 | 0.133559 | 0.939731 |
| 12. | 6. | 0. | -0.615452 | -0.776502 | -0.048713 |
| 13. | 6. | 0. | 0.649198 | -0.847987 | -0.519152 |
| 14. | 6. | 0. | -2.319816 | 0.349551 | 1.342147 |
| 15. | 6. | 0. | -3.369691 | -0.321113 | 0.450775 |
| 16. | 7. | 0. | -2.891465 | -1.586070 | -0.059562 |
| 17. | 6. | 0. | -1.612614 | -1.802301 | -0.493151 |
| 18. | 6. | 0. | -4.670220 | -0.725768 | 1.155013 |
| 19. | 6. | 0. | -5.229256 | -1.809621 | 0.213870 |
| 20. | 6. | 0. | -3.977875 | -2.541365 | -0.315252 |
| 21. | 8. | 0. | -1.300119 | -2.795464 | -1.145142 |
| 22. | 8. | 0. | -2.615935 | 1.077687 | 2.280386 |
| 23. | 6. | 0. | 5.104946 | -1.573914 | 0.716032 |
| 24. | 6. | 0. | 3.977282 | -2.142531 | -1.495950 |
| 25. | 6. | 0. | 6.335139 | -1.654683 | 0.206441 |
| 26. | 6. | 0. | 2.943744 | -2.752430 | 0.730632 |
| 27. | 8. | 0. | -3.591475 | 0.520218 | -0.697113 |
| 28. | 6. | 0. | -4.131326 | 1.829896 | -0.475327 |
| 29. | 6. | 0. | -4.113561 | 2.553006 | -1.811963 |
| 30. | 1. | 0. | 4.413955 | 3.595392 | 0.406947 |
| 31. | 1. | 0. | -0.291119 | 2.169726 | -0.592914 |
| 32. | 1. | 0. | 0.287666 | 4.563805 | -0.385610 |
| 33. | 1. | 0. | 2.601870 | 5.277614 | 0.121567 |
| 34. | 1. | 0. | 4.837484 | 0.789249 | 0.335812 |
| 35. | 1. | 0. | -0.320100 | 0.658702 | 1.437009 |
| 36. | 1. | 0. | 0.814415 | -1.690046 | -1.183941 |
| 37. | 1. | 0. | -4.422693 | -1.137196 | 2.138315 |
| 38. | 1. | 0. | -5.345568 | 0.115510 | 1.312400 |
| 39. | 1. | 0. | -5.921351 | -2.487907 | 0.718186 |
| 40. | 1. | 0. | -5.764170 | -1.338424 | -0.615122 |
| 41. | 1. | 0. | -3.772006 | -3.473009 | 0.223155 |
| 42. | 1. | 0. | -4.035792 | -2.783935 | -1.379894 |
| 43. | 1. | 0. | 4.997122 | -1.415243 | 1.790085 |

| | | | | | |
|---|---|---|---|---|---|
| 44. | 1. | 0. | 3.010268 | -2.247569 | -1.993874 |
| 45. | 1. | 0. | 4.564692 | -1.414933 | -2.063501 |
| 46. | 1. | 0. | 4.487843 | -3.110036 | -1.536948 |
| 47. | 1. | 0. | 6.522155 | -1.817345 | -0.850372 |
| 48. | 1. | 0. | 7.210141 | -1.578111 | 0.845191 |
| 49. | 1. | 0. | 2.757385 | -2.437542 | 1.761972 |
| 50. | 1. | 0. | 1.978210 | -2.930035 | 0.254752 |
| 51. | 1. | 0. | 3.491352 | -3.699743 | 0.755900 |
| 52. | 1. | 0. | -5.159647 | 1.761337 | -0.092981 |
| 53. | 1. | 0. | -3.540968 | 2.371864 | 0.273575 |
| 54. | 1. | 0. | -3.090781 | 2.644687 | -2.188400 |
| 55. | 1. | 0. | -4.536067 | 3.556961 | -1.704200 |
| 56. | 1. | 0. | -4.702104 | 2.006425 | -2.554384 |
